# Supplementary material for: Bifunctionality of dirhodium tetracarboxylates in metallaphotocatalysis
Source: Nat Commun. 2023 Nov 10;14:7269. doi: 10.1038/s41467-023-43050-3 (PMC10638314; doi:10.1038/s41467-023-43050-3)
Supplement: Supplementary file 1 — Supplementary Information [file 41467_2023_43050_MOESM1_ESM.pdf]

Supplementary Information

Bifunctionality of Dirhodium Tetracarboxylates in Metallaphotocatalysis

Taoda Shi [a, ‡, *], Tianyuan Zhang [a, ‡], Jiying Yang [a], Yukai Li [a], Jirong Shu [a], Jingyu Zhao [a], Mengchu Zhang [a], Dan Zhang [a], Wenhao Hu [a, *].

[a] Guangdong Key Laboratory of Chiral Molecule and Drug Discovery, School of Pharmaceutical Sciences, Sun Yat-Sen University, Guangzhou 510006, China

‡Equal contribution.

*E-mail: shitd@mail.sysu.edu.cn; huwh9@mail.sysu.edu.cn.

Table of Content

1. General information & materials ········· 3

3. General procedures for the synthesis of 6 ········· 4

4.Gram scale synthesis of 6a ········· 4

5. Characterization data of product 6 ········· 5

6. Transformations of product 6 ········· 19

7. Data of product 10 ········· 24

8. Evaluation of the function of $Rh_2(OAc)_4$ and $Rh_2(esp)_2$ as photosensitizer for generation of singlet oxygen ········· 32

9. Supplementary optimization of reactions ········· 34

10. Control experiments and the inert substrates of the PCR ········· 35

11. NMR Spectra ········· 40

12. Single Crystal X-ray Diffraction Data ········· 88

13. Computational Studies ········· 90

14. Molecular docking ········· 91

15. Biological activity evaluation ········· 92

16. References ········· 94

1. General information & materials

All ^{1}H NMR, ^{13}C NMR and ^{19}F NMR spectra were recorded on Brucker Avance 400 MHz or Ascend 500 MHz spectrometers in deuterated solvent included $CDCl_3$ and DMSO-d_6. Tetramethylsilane (TMS) was used as an internal standard (δ = 0) for ^{1}H NMR, and $CDCl_3$ was used as internal standard (δ = 77.0) for ^{13}C NMR. Chemical shifts are reported in parts per million (ppm) as follows: chemical shift, multiplicity (s = singlet, d = doublet, t = triplet, q = quartet, m = multiplet, br =broad). NMR data were processed via MestRenova 10.0.0. High-resolution mass spectrometry (HRMS) was performed on Waters Micromass Q-TOF micro Synapt High Definition Mass Spectrometer. Single crystal X-ray diffraction data were recorded on Bruker-AXS SMART APEX II single crystal X-ray diffractometer. Yields for all compounds were isolated yields for all isomers.

2. General procedure for the synthesis of cycloprop-2-ene-1-ester 5.

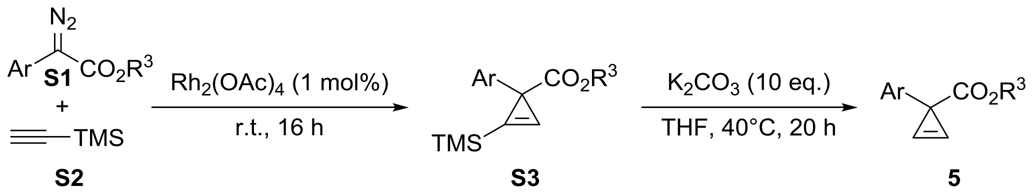

Supplementary Figure 1. Preparation of cycloprop-2-ene-1-esters

The procedure was generated by slightly revising the reported procedure of synthesizing cycloprop-2-ene-1-ester[1]. As shown in Supplementary Fig. 1, a solution of **S1** (24 mmol) in trimethylsilylacetylene **S2** (10 mL) was added to a stirred suspension of $Rh_2(OAc)_4$ (1 mol%) in trimethylsilylacetylene (20 mL) using syringe

pump over 16 h. After the addition was complete, the syringe was washed with anhydrous CH_2Cl_2 (4 mL). The mixture was stirred until the thin layer chromatography (TLC) shown the reaction was complete. After concentrating under reduced pressure, the crude residue was purified by silica gel column chromatography to afford **S3**.

To a clean round bottomed flask was added **S3** (20 mmol), H_2O (30 mL), THF (30 mL) and 10 eq. K_2CO_3 (200 mmol). The reaction was stirred at 40 ℃ for twenty hours. The THF was removed under reduced pressure, and the mixture was extracted three times with CH_2Cl_2. The combined organics were washed with brine, dried over Na_2SO_4, filtered and concentrated in vacuum. The residue was purified by silica gel column chromatography to afford **5**.

3. General procedures for the synthesis of 6

To a solution of isatin **4** (0.20 mmol, 1.0 equiv), $Rh_2(esp)_2$ (3.0 mg, 2 mol%) and acetic acid (0.30 mmol, 1.5 equiv.) in ethyl acetate (3 mL) was added Cycloprop-2-ene-1-ester **5** (0.30 mmol, 1.5 equiv.). The reaction was stirred at 35°C under nitrogen atmosphere for 16 h, then the reaction was stirred at 25°C under air atmosphere and visible light irradiation (12 W white LEDs or sunlight) for 8-12 h. After complete consumption of the reaction intermediate **6a-Int** monitored by TLC, the reaction mixture was filtered, concentrated under vacuum. The residue was purified by silica gel flash column chromatography (petroleum ether/EtOAc = 10/1~3/1) to afford **6**, *Z* : *E* > 20:1.

4. Gram scale synthesis of 6a

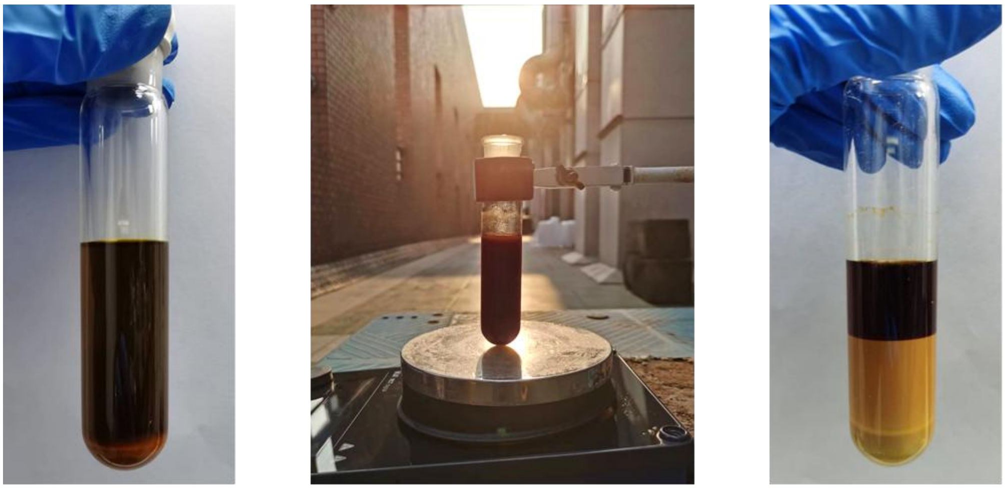

Before **Middle** **After**

Supplementary Figure 2. Reaction mixture when the reaction started (left) and the product **6a** was precipitated when the reaction was finished (right).

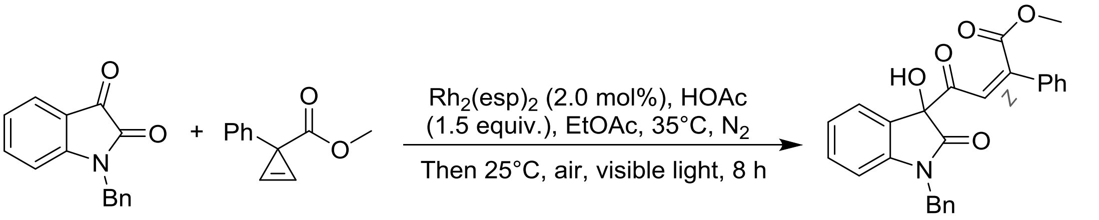

4a 0.95 g, 4.0 mmol **5a** 1.04 g, 6.0 mmol **6a**, 1.37 g 80% yield, *Z* : *E* > 20 : 1

Supplementary Figure 3. Gram-scale synthesis of PCR product **6a**.

To a solution of **4a** (0.95 g, 4.0 mmol), $Rh_2(esp)_2$ (60.7 mg, 0.08 mmol, 2 mol%) and acetic acid (0.36 g, 6.0 mmol) in EtOAc (20 mL) was added **5a** (1.04 g, 6.0 mmol). The reaction was stirred at 35°C under nitrogen atmosphere for 24 h, then the reaction was stirred at 25°C under air atmosphere and visible light irradiation by sunlight for 8 h (Supplementary Fig. 2). After complete consumption of the reaction intermediate **6a-Int** monitored by TLC, the reaction mixture was filtered, concentrated under vacuum. The residue was purified by silica gel flash column chromatography (petroleum ether/EtOAc = 10/1~4/1) to afford **6a** as a white solid (Supplementary Fig. 3, 1.37 g, 80%, *Z* : *E* > 20:1)

5. Characterization data of product 6

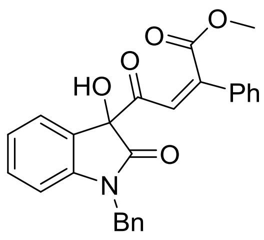

6a

methyl (*Z*)-4-(1-benzyl-3-hydroxy-2-oxoindolin-3-yl)-4-oxo-2-phenylbut-2-enoate (6a)

White solid; 81% yield.

^{1}H NMR (500 MHz, Chloroform-*d*) δ 7.43 – 7.38 (m, 3H), 7.35 – 7.27 (m, 6H), 7.22 (d, *J* = 7.5 Hz, 1H), 7.14 (d, *J* = 7.8 Hz, 2H), 7.09 (t, *J* = 7.6 Hz, 1H), 6.93 (d, *J* = 7.9 Hz, 1H), 6.09 (s, 1H), 5.27 (d, *J* = 15.3 Hz, 1H), 5.08 (s, 1H), 4.65 (d, *J* = 15.3 Hz, 1H), 3.98 (s, 3H).

^{13}C NMR (126 MHz, Chloroform-*d*) δ 189.96, 172.60, 167.99, 150.94, 143.88, 135.46, 132.38, 131.61, 131.14, 129.11, 129.08, 128.06, 127.75, 127.21, 125.63, 124.91, 124.08, 115.86, 109.90, 83.17, 53.05, 44.55.

HRMS (ESI) calcd for $C_{26}H_{21}NO_5Na$ $[M+Na]^+$: 450.1312, found 450.1313.

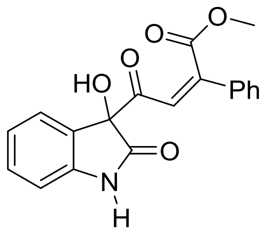

6b

methyl (*Z*)-4-(3-hydroxy-2-oxoindolin-3-yl)-4-oxo-2-phenylbut-2-enoate (6b)

White solid; 76% yield.

^{1}H NMR (500 MHz, Chloroform-*d*) δ 8.65 (s, 1H), 7.44 – 7.30 (m, 6H), 7.22 (d, *J* = 7.5 Hz, 1H), 7.11 (t, *J* = 7.6 Hz, 1H), 7.02 (d, *J* = 7.9 Hz, 1H), 6.32 (s, 1H), 5.10 (s, 1H), 3.98 (s, 3H).

^{13}C NMR (126 MHz, Chloroform-*d*) δ 189.85, 174.75, 168.07, 151.27, 141.78, 132.47, 131.70, 131.29, 129.14, 127.31, 126.12, 125.16, 124.13, 115.75, 111.02, 83.46, 53.06.

HRMS (ESI) calcd for $C_{19}H_{15}NO_5Na$ $[M+Na]^+$: 360.0842, found 360.0844.

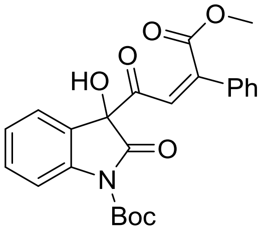

6c

tert-butyl (*Z*)-3-hydroxy-3-(4-methoxy-4-oxo-3-phenylbut-2-enoyl)-2-oxoindoline-1-carboxylate (6c)

Yellow oil; 71% yield.

^{1}H NMR (500 MHz, Chloroform-*d*) δ 8.00 (d, *J* = 8.2 Hz, 1H), 7.48 (td, *J* = 8.6, 7.9, 2.0 Hz, 1H), 7.44 (t, *J* = 7.2 Hz, 1H), 7.36 (t, *J* = 7.7 Hz, 2H), 7.31 (d, *J* = 7.6 Hz, 2H), 7.26 (d, *J* = 5.7 Hz, 1H), 7.24 (t, *J* = 7.3 Hz, 1H), 6.21 (s, 1H), 5.10 (s, 1H), 3.97 (s, 3H), 1.65 (s, 9H).

^{13}C NMR (126 MHz, Chloroform-*d*) δ 189.41, 170.49, 167.92, 151.50, 148.76, 141.05, 132.50, 131.80, 131.46, 129.18, 127.37, 125.72, 124.72, 115.93, 115.91, 85.45, 82.67, 53.08, 28.04.

HRMS (ESI) calcd for $C_{24}H_{23}NO_7Na$ $[M+Na]^+$: 460.1367, found 460.1367.

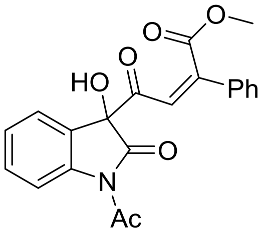

6d

methyl (*Z*)-4-(1-acetyl-3-hydroxy-2-oxoindolin-3-yl)-4-oxo-2-phenylbut-2-enoate (6d)

Light yellow solid; 70% yield.

^{1}H NMR (400 MHz, Chloroform-*d*) δ 8.35 (d, *J* = 8.3 Hz, 1H), 7.54 – 7.39 (m, 3H), 7.36 (t, *J* = 7.6 Hz, 2H), 7.33 – 7.27 (m, 3H), 6.20 (s, 1H), 5.15 (s, 1H), 3.98 (s, 3H), 2.70 (s,

3H).

^{13}C NMR (126 MHz, Chloroform-*d*) δ 189.23, 173.10, 170.49, 167.82, 151.99, 141.28, 132.27, 132.00, 131.60, 129.26, 127.35, 126.48, 125.03, 124.50, 117.37, 115.37, 82.87, 53.12, 26.58.

HRMS (ESI) calcd for $C_{21}H_{17}NO_6Na$ [M+Na]$^+$: 402.0948, found 402.0948.

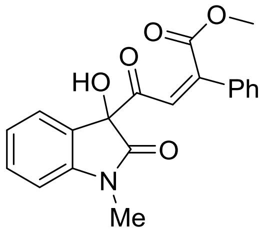

6e

methyl (*Z*)-4-(3-hydroxy-1-methyl-2-oxoindolin-3-yl)-4-oxo-2-phenylbut-2-enoate (6e)

Light yellow oil; 68% yield.

^{1}H NMR (400 MHz, Chloroform-*d*) δ 7.43 (q, *J* = 7.4 Hz, 2H), 7.34 (t, *J* = 7.6 Hz, 2H), 7.32 – 7.27 (m, 2H), 7.25 (d, *J* = 7.4 Hz, 1H), 7.13 (t, *J* = 7.5 Hz, 1H), 6.97 (d, *J* = 7.8 Hz, 1H), 6.19 (s, 1H), 5.03 (s, 1H), 3.96 (s, 3H), 3.31 (s, 3H).

^{13}C NMR (101 MHz, Chloroform-*d*) δ 190.35, 172.69, 168.04, 150.95, 144.86, 132.73, 131.56, 131.27, 129.12, 127.24, 125.71, 124.86, 124.08, 116.23, 109.09, 83.07, 53.04, 27.06.

HRMS (ESI) calcd for $C_{20}H_{17}NO_5Na$ [M+Na]$^+$: 374.0999, found 374.0999.

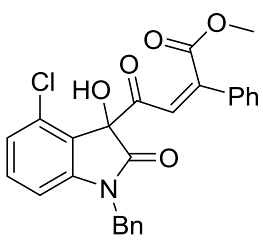

6f

methyl (*Z*)-4-(1-benzyl-4-chloro-3-hydroxy-2-oxoindolin-3-yl)-4-oxo-2-phenylbut-2-enoate (6f)

White solid; 78% yield.

^{1}H NMR (500 MHz, Chloroform-*d*) δ 7.43 (t, *J* = 7.4 Hz, 1H), 7.37 (dd, *J* = 7.3, 2.1 Hz, 2H), 7.33 – 7.26 (m, 6H), 7.18 (d, *J* = 7.7 Hz, 2H), 7.02 (d, *J* = 8.2 Hz, 1H), 6.82 (d, *J* = 7.9 Hz, 1H), 6.08 (s, 1H), 5.23 (d, *J* = 15.4 Hz, 1H), 5.13 (s, 1H), 4.67 (d, *J* = 15.3 Hz, 1H), 3.99 (s, 3H).

^{13}C NMR (126 MHz, Chloroform-*d*) δ 188.48, 171.44, 167.76, 151.42, 145.49, 135.09, 132.38, 132.36, 132.25, 131.73, 129.18, 129.10, 128.22, 127.67, 127.30, 124.68, 122.95, 115.43, 108.25, 82.89, 53.09, 44.73.

HRMS (ESI) calcd for $C_{26}H_{20}NO_5ClNa$ $[M+Na]^+$: 484.0922, found 484.0922.

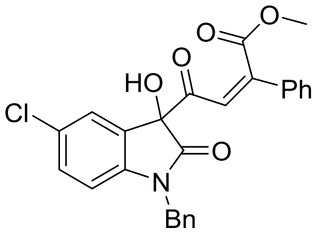

6g

methyl (*Z*)-4-(1-benzyl-5-chloro-3-hydroxy-2-oxoindolin-3-yl)-4-oxo-2-phenylbut-2-enoate (6g)

White solid; 68% yield.

^{1}H NMR (400 MHz, Chloroform-*d*) δ 7.43 (t, *J* = 7.4 Hz, 1H), 7.37 (dd, *J* = 7.3, 2.1 Hz, 2H), 7.34 – 7.26 (m, 6H), 7.21 (d, *J* = 2.1 Hz, 1H), 7.17 (d, *J* = 7.7 Hz, 2H), 6.84 (d, *J* = 8.4 Hz, 1H), 6.08 (s, 1H), 5.26 (d, *J* = 15.3 Hz, 1H), 5.08 (s, 1H), 4.62 (d, *J* = 15.3 Hz, 1H), 3.98 (s, 3H).

^{13}C NMR (126 MHz, Chloroform-*d*) δ 189.19, 172.18, 167.81, 151.62, 142.37, 135.04, 132.26, 131.84, 131.09, 129.55, 129.21, 129.14, 128.25, 127.72, 127.32, 127.25, 125.35, 115.36, 110.91, 82.92, 53.12, 44.69.

HRMS (ESI) calcd for $C_{26}H_{20}NO_5ClNa$ $[M+Na]^+$: 484.0922, found 484.0922.

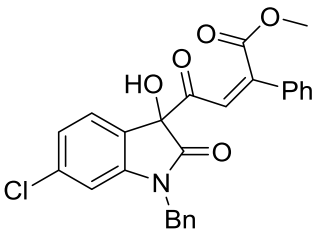

6h

methyl (Z)-4-(1-benzyl-6-chloro-3-hydroxy-2-oxoindolin-3-yl)-4-oxo-2-phenylbut-2-enoate (6h)

White solid; 73% yield.

^{1}H NMR (400 MHz, Chloroform-*d*) δ 7.43 (t, *J* = 7.4 Hz, 1H), 7.38 (dd, *J* = 7.2, 2.3 Hz, 2H), 7.36 – 7.27 (m, 5H), 7.20 – 7.11 (m, 3H), 7.07 (d, *J* = 8.0 Hz, 1H), 6.92 (s, 1H), 6.07 (s, 1H), 5.25 (d, *J* = 15.4 Hz, 1H), 5.06 (s, 1H), 4.60 (d, *J* = 15.4 Hz, 1H), 3.97 (s, 3H).

^{13}C NMR (126 MHz, Chloroform-*d*) δ 189.37, 172.56, 167.88, 151.49, 145.06, 137.03, 134.95, 132.23, 131.82, 129.27, 129.15, 128.30, 127.71, 127.29, 125.88, 124.08, 124.00, 115.42, 110.59, 82.61, 53.10, 44.67.

HRMS (ESI) calcd for $C_{26}H_{20}NO_5ClNa$ $[M+Na]^+$: 484.0922, found 484.0922.

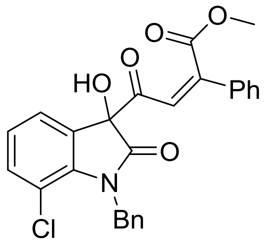

6i

methyl (*Z*)-4-(1-benzyl-7-chloro-3-hydroxy-2-oxoindolin-3-yl)-4-oxo-2-phenylbut-2-enoate (6i)

White solid; 70% yield.

^{1}H NMR (400 MHz, Chloroform-*d*) δ 7.44 (t, *J* = 7.4 Hz, 1H), 7.41 – 7.28 (m, 6H), 7.27 – 7.25 (m, 2H), 7.19 (d, *J* = 7.6 Hz, 2H), 7.14 (d, *J* = 7.3 Hz, 1H), 7.04 (t, *J* = 8.0 Hz,

1H), 6.12 (s, 1H), 5.45 (d, *J* = 15.8 Hz, 1H), 5.38 (d, *J* = 15.8 Hz, 1H), 5.09 (s, 1H), 3.97 (s, 3H).

^{13}C NMR (126 MHz, Chloroform-*d*) δ 189.30, 173.23, 167.89, 151.62, 140.00, 136.87, 133.77, 132.32, 131.80, 129.16, 128.85, 128.51, 127.65, 127.36, 127.17, 124.96, 123.63, 116.18, 115.65, 82.16, 53.08, 45.49.

HRMS (ESI) calcd for $C_{26}H_{20}NO_5ClNa$ $[M+Na]^+$: 484.0922, found 484.0922.

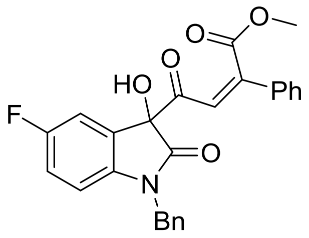

6j

methyl (*Z*)-4-(1-benzyl-5-fluoro-3-hydroxy-2-oxoindolin-3-yl)-4-oxo-2-phenylbut-2-enoate (6j)

White solid; 82% yield.

^{1}H NMR (400 MHz, Chloroform-*d*) δ 7.43 (t, *J* = 7.4 Hz, 1H), 7.37 (dd, *J* = 7.3, 2.2 Hz, 2H), 7.35 – 7.26 (m, 5H), 7.16 (d, *J* = 7.5 Hz, 2H), 7.03 (td, *J* = 8.7, 2.6 Hz, 1H), 6.98 (dd, *J* = 7.2, 2.6 Hz, 1H), 6.85 (dd, *J* = 8.6, 3.9 Hz, 1H), 6.09 (s, 1H), 5.26 (d, *J* = 15.3 Hz, 1H), 5.07 (s, 1H), 4.62 (d, *J* = 15.4 Hz, 1H), 3.98 (s, 3H).

^{13}C NMR (126 MHz, Chloroform-*d*) δ 189.31, 172.35, 167.83, 160.66, 158.72, 151.52, 139.79, 135.15, 132.26, 131.81, 129.20, 129.14, 128.21, 127.72, 127.28, 117.59 (d, *J* = 23.6 Hz), 115.41, 113.03 (d, *J* = 25.4 Hz), 110.70 (d, *J* = 7.9 Hz), 83.15, 53.12, 44.72.

^{19}F NMR (376 MHz, Chloroform-*d*) δ -117.80.

HRMS (ESI) calcd for $C_{26}H_{20}NO_5FNa$ $[M+Na]^+$: 468.1218, found 468.1218.

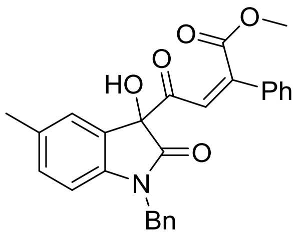

6k

methyl (*Z*)-4-(1-benzyl-3-hydroxy-5-methyl-2-oxoindolin-3-yl)-4-oxo-2-phenylbut-2-enoate (6k)

White solid; 80% yield.

^{1}H NMR (400 MHz, Chloroform-*d*) δ 7.45 – 7.35 (m, 3H), 7.32 – 7.24 (m, 5H), 7.14 (d, *J* = 7.8 Hz, 2H), 7.11 (d, *J* = 7.8 Hz, 1H), 7.03 (s, 1H), 6.81 (d, *J* = 8.0 Hz, 1H), 6.09 (s, 1H), 5.25 (d, *J* = 15.3 Hz, 1H), 5.03 (s, 1H), 4.62 (d, *J* = 15.3 Hz, 1H), 3.98 (s, 3H), 2.27 (s, 3H).

^{13}C NMR (101 MHz, Chloroform-*d*) δ 190.03, 172.50, 168.05, 150.86, 141.46, 135.61, 133.91, 132.40, 131.59, 131.43, 129.08, 128.01, 127.78, 127.23, 125.57, 115.85, 109.67, 83.29, 53.05, 44.58, 20.97.

HRMS (ESI) calcd for $C_{27}H_{23}NO_5Na$ $[M+Na]^+$: 464.1468, found 464.1468.

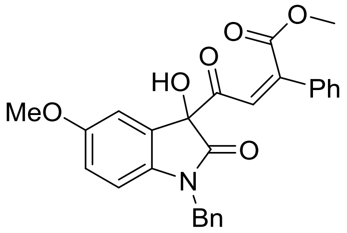

6l

methyl (*Z*)-4-(1-benzyl-3-hydroxy-5-methoxy-2-oxoindolin-3-yl)-4-oxo-2-phenylbut-2-enoate (6l)

Yellow solid; 78% yield.

^{1}H NMR (500 MHz, Chloroform-*d*) δ 7.42 (t, *J* = 7.4 Hz, 1H), 7.39 (dd, *J* = 7.4, 2.1 Hz, 2H), 7.32 – 7.26 (m, 5H), 7.15 (dd, *J* = 8.4, 1.3 Hz, 2H), 6.89 – 6.78 (m, 3H), 6.10 (s, 1H), 5.26 (d, *J* = 15.3 Hz, 1H), 5.08 (s, 1H), 4.61 (d, *J* = 15.3 Hz, 1H), 3.98 (s, 3H),

3.73 (s, 3H).

^{13}C NMR (126 MHz, Chloroform-*d*) δ 189.93, 172.27, 168.02, 156.86, 151.03, 137.05, 135.55, 132.36, 131.65, 129.10, 128.04, 127.76, 127.25, 126.70, 116.32, 115.74, 111.09, 110.62, 83.52, 55.79, 53.07, 44.66.

HRMS (ESI) calcd for $C_{27}H_{23}NO_6Na$ $[M+Na]^+$: 480.1418, found 480.1418.

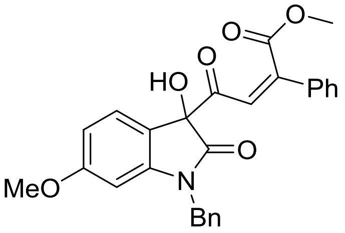

6m

methyl (*Z*)-4-(1-benzyl-3-hydroxy-6-methoxy-2-oxoindolin-3-yl)-4-oxo-2-phenylbut-2-enoate (6m)

Yellow solid; 77% yield.

^{1}H NMR (500 MHz, Chloroform-*d*) δ 7.44 – 7.37 (m, 3H), 7.35 – 7.26 (m, 5H), 7.15 (d, *J* = 7.3 Hz, 2H), 7.12 (d, *J* = 8.3 Hz, 1H), 6.55 (dd, *J* = 8.3, 2.3 Hz, 1H), 6.49 (d, *J* = 2.3 Hz, 1H), 6.08 (s, 1H), 5.24 (d, *J* = 15.3 Hz, 1H), 5.00 (s, 1H), 4.60 (d, *J* = 15.3 Hz, 1H), 3.97 (s, 3H), 3.78 (s, 3H).

^{13}C NMR (126 MHz, Chloroform-*d*) δ 190.20, 173.04, 168.07, 162.22, 150.72, 145.31, 135.49, 132.44, 131.56, 129.13, 129.07, 128.07, 127.77, 127.21, 125.92, 117.34, 116.01, 107.35, 98.32, 82.76, 55.58, 53.03, 44.55.

HRMS (ESI) calcd for $C_{27}H_{23}NO_6Na$ $[M+Na]^+$: 480.1418, found 480.1417.

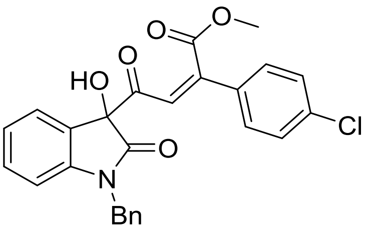

6n

methyl

(*Z*)-4-(1-benzyl-3-hydroxy-2-oxoindolin-3-yl)-2-(4-chlorophenyl)-4-oxobut-2-enoate (6n)

White solid; 79% yield.

^{1}H NMR (500 MHz, Chloroform-*d*) δ 7.41 (d, *J* = 7.2 Hz, 2H), 7.38 – 7.26 (m, 5H), 7.25 – 7.20 (m, 2H), 7.09 (t, *J* = 7.6 Hz, 1H), 7.03 (d, *J* = 8.6 Hz, 2H), 6.95 (d, *J* = 7.9 Hz, 1H), 6.02 (s, 1H), 5.27 (d, *J* = 15.2 Hz, 1H), 5.02 (s, 1H), 4.64 (d, *J* = 15.2 Hz, 1H), 3.97 (s, 3H).

^{13}C NMR (126 MHz, Chloroform-*d*) δ 189.89, 172.45, 167.64, 149.56, 143.84, 137.88, 135.51, 131.22, 130.85, 129.36, 129.13, 128.45, 128.15, 127.88, 125.49, 124.95, 124.14, 116.27, 109.88, 83.20, 53.18, 44.55.

HRMS (ESI) calcd for $C_{26}H_{20}NO_5ClNa$ $[M+Na]^+$: 484.0922, found 484.0920.

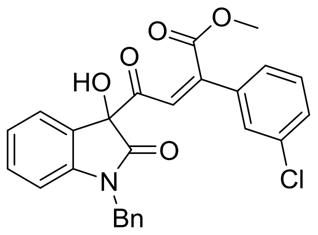

6o

methyl (*Z*)-4-(1-benzyl-3-hydroxy-2-oxoindolin-3-yl)-2-(3-chlorophenyl)-4-oxobut-2-enoate (6o)

White solid; 80% yield.

^{1}H NMR (500 MHz, Chloroform-*d*) δ 7.40 – 7.27 (m, 7H), 7.25 – 7.20 (m, 2H), 7.16 (s, 1H), 7.10 (t, *J* = 7.5 Hz, 1H), 7.01 (d, *J* = 7.9 Hz, 1H), 6.93 (d, *J* = 7.9 Hz, 1H), 6.06 (s, 1H), 5.26 (d, *J* = 15.3 Hz, 1H), 4.99 (s, 1H), 4.65 (d, *J* = 15.3 Hz, 1H), 3.97 (s, 3H).

^{13}C NMR (126 MHz, Chloroform-*d*) δ 190.08, 172.38, 167.45, 149.40, 143.89, 135.34, 135.19, 134.30, 131.46, 131.30, 130.30, 129.14, 128.21, 127.62, 127.07, 125.37, 125.33, 124.94, 124.15, 117.36, 110.03, 83.21, 53.24, 44.60.

HRMS (ESI) calcd for $C_{26}H_{20}NO_5ClNa$ $[M+Na]^+$: 484.0922, found 484.0920.

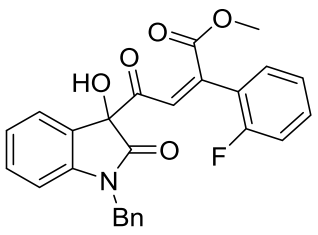

6p

methyl (*Z*)-4-(1-benzyl-3-hydroxy-2-oxoindolin-3-yl)-2-(2-fluorophenyl)-4-oxobut-2-enoate (6p)

White solid; 82% yield.

^{1}H NMR (500 MHz, Chloroform-*d*) δ 7.38 (tdd, *J* = 6.9, 5.0, 1.9 Hz, 1H), 7.36 – 7.29 (m, 3H), 7.27 – 7.22 (m, 4H), 7.15 (td, *J* = 7.7, 1.9 Hz, 1H), 7.10 (q, *J* = 7.2 Hz, 2H), 7.03 (dd, *J* = 11.6, 8.4 Hz, 1H), 6.86 (d, *J* = 7.9 Hz, 1H), 6.34 (s, 1H), 5.17 (d, *J* = 15.2 Hz, 1H), 5.01 (s, 1H), 4.73 (d, *J* = 15.5 Hz, 1H), 3.95 (s, 3H).

^{13}C NMR (126 MHz, Chloroform-*d*) δ 190.73, 172.45, 167.41, 160.82 (d, *J* = 255.6 Hz), 144.64, 143.94, 135.16, 132.83 (d, *J* = 9.3 Hz), 131.15, 130.10, 128.93, 127.95, 127.51, 125.45, 124.89, 124.76 (d, *J* = 3.6 Hz), 124.03, 121.24 (d, *J* = 10.7 Hz), 120.90 (d, *J* = 9.7 Hz), 116.72 (d, *J* = 22.7 Hz), 110.03, 83.37, 53.17, 44.57.

^{19}F NMR (376 MHz, Chloroform-*d*) δ -110.05 (dt, *J* = 12.2, 6.3 Hz).

HRMS (ESI) calcd for $C_{26}H_{20}NO_5FNa$ $[M+Na]^+$: 468.1218, found 468.1216.

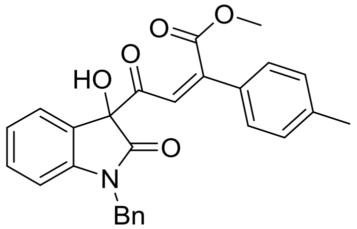

6q

methyl (*Z*)-4-(1-benzyl-3-hydroxy-2-oxoindolin-3-yl)-4-oxo-2-(p-tolyl)but-2-enoate (6q)

White solid; 81% yield.

^{1}H NMR (500 MHz, Chloroform-*d*) δ 7.43 – 7.37 (m, 2H), 7.36 – 7.27 (m, 4H), 7.21 (d, *J* = 7.4 Hz, 1H), 7.15 – 6.98 (m, 5H), 6.92 (d, *J* = 7.9 Hz, 1H), 6.05 (s, 1H), 5.27 (d, *J* =

15.2 Hz, 1H), 5.08 (s, 1H), 4.64 (d, *J* = 15.3 Hz, 1H), 3.97 (s, 3H), 2.35 (s, 3H).

^{13}C NMR (126 MHz, Chloroform-*d*) δ 189.81, 172.65, 168.19, 151.07, 143.90, 142.57, 135.53, 131.06, 129.81, 129.52, 129.12, 128.04, 127.76, 127.25, 125.78, 124.90, 124.06, 114.53, 109.85, 83.06, 53.00, 44.55, 21.52.

HRMS (ESI) calcd for $C_{27}H_{23}NO_5Na$ $[M+Na]^+$: 464.1468, found 464.1464.

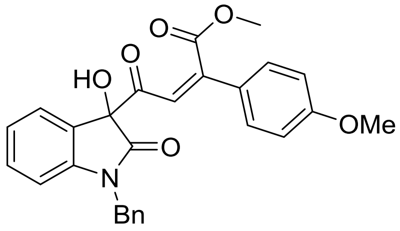

6r

methyl (*Z*)-4-(1-benzyl-3-hydroxy-2-oxoindolin-3-yl)-2-(4-methoxyphenyl)-4-oxobut-2-enoate (6r)

White solid; 80% yield.

^{1}H NMR (500 MHz, Chloroform-*d*) δ 7.41 (dd, *J* = 6.9, 2.8 Hz, 2H), 7.35 – 7.27 (m, 4H), 7.21 (d, *J* = 7.4 Hz, 1H), 7.12 – 7.02 (m, 3H), 6.93 (d, *J* = 7.9 Hz, 1H), 6.78 (d, *J* = 8.8 Hz, 2H), 5.98 (s, 1H), 5.28 (d, *J* = 15.3 Hz, 1H), 5.11 (s, 1H), 4.63 (d, *J* = 15.3 Hz, 1H), 3.98 (s, 3H), 3.82 (s, 3H).

^{13}C NMR (126 MHz, Chloroform-*d*) δ 189.54, 172.75, 168.37, 162.59, 150.73, 143.88, 135.61, 131.00, 129.25, 129.13, 128.04, 127.81, 125.96, 124.87, 124.57, 124.04, 114.55, 112.81, 109.80, 82.97, 55.53, 53.03, 44.52.

HRMS (ESI) calcd for $C_{27}H_{23}NO_6Na$ $[M+Na]^+$: 480.1418, found 480.1420.

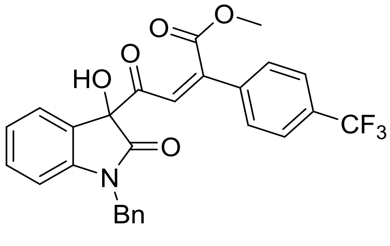

6s

methyl

(*Z*)-4-(1-benzyl-3-hydroxy-2-oxoindolin-3-yl)-4-oxo-2-(4-(trifluoromethyl)phenyl) but-2-enoate (6s)

White solid; 77% yield.

^{1}H NMR (400 MHz, Chloroform-*d*) δ 7.66 (d, *J* = 7.8 Hz, 1H), 7.45 – 7.40 (m, 2H), 7.39 – 7.33 (m, 3H), 7.27 (d, *J* = 6.3 Hz, 1H), 7.26 – 7.20 (m, 4H), 7.11 (td, *J* = 7.6, 1.0 Hz, 1H), 6.93 (d, *J* = 7.9 Hz, 1H), 6.10 (s, 1H), 5.22 (d, *J* = 15.3 Hz, 1H), 4.96 (s, 1H), 4.68 (d, *J* = 15.3 Hz, 1H), 3.98 (s, 3H).

^{13}C NMR (126 MHz, Chloroform-*d*) δ 190.21, 172.34, 167.28, 149.04, 143.87, 135.27, 133.57, 131.67 (q, *J* = 32.8 Hz), 131.32, 130.17, 129.68, 129.03, 128.13, 127.85 (q, *J* = 3.6 Hz), 127.64, 125.32, 124.96, 124.17, 123.95 (q, *J* = 3.9 Hz), 123.35 (q, *J* = 272.7 Hz), 118.32, 110.02, 83.29, 53.29, 44.60.

^{19}F NMR (376 MHz, Chloroform-*d*) δ -62.91.

HRMS (ESI) calcd for $C_{27}H_{20}NO_5F_3Na$ $[M+Na]^+$: 518.1186, found 518.1184.

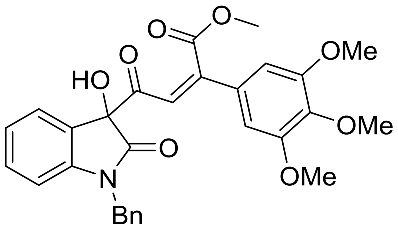

6t

methyl (*Z*)-4-(1-benzyl-3-hydroxy-2-oxoindolin-3-yl)-4-oxo-2-(3,4,5-trimethoxyphenyl)but-2-enoate (6t)

White solid; 49% yield; 86:14 dr.

^{1}H NMR (500 MHz, Chloroform-*d*) δ 7.34 – 7.29 (m, 3H), 7.25 (d, *J* = 7.5 Hz, 1H), 7.23 – 7.14 (m, 3H), 7.10 (t, *J* = 7.6 Hz, 1H), 6.87 (d, *J* = 7.9 Hz, 1H), 6.35 (s, 2H), 6.04 (s, 1H), 5.26 (d, *J* = 15.4 Hz, 1H), 5.04 (s, 1H), 4.64 (d, *J* = 15.4 Hz, 1H), 3.97 (s, 3H), 3.86 (s, 3H), 3.64 (s, 6H).

^{13}C NMR (126 MHz, Chloroform-*d*) δ 189.78, 172.62, 167.85, 153.38, 150.64, 143.93, 141.38, 135.27, 130.94, 129.02, 128.04, 127.94, 127.45, 125.87, 124.94, 124.11, 116.09, 109.80, 104.72, 83.28, 60.99, 56.14, 53.15, 44.60.

HRMS (ESI) calcd for $C_{29}H_{27}NO_8Na$ $[M+Na]^+$: 540.1629, found 540.1630.

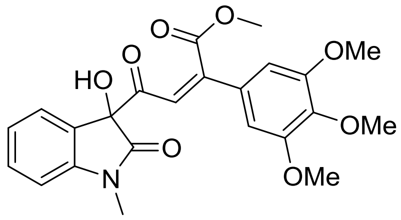

6u

methyl

(*Z*)-4-(3-hydroxy-1-methyl-2-oxoindolin-3-yl)-4-oxo-2-(3,4,5-trimethoxyphenyl)but-2-enoate (6u)

White solid; 75% yield.

^{1}H NMR (500 MHz, Chloroform-*d*) δ 7.43 (td, *J* = 7.8, 1.3 Hz, 1H), 7.25 (d, *J* = 6.5 Hz, 1H), 7.14 (t, *J* = 7.6 Hz, 1H), 6.95 (d, *J* = 7.8 Hz, 1H), 6.48 (s, 2H), 6.06 (s, 1H), 4.95 (br.s, 1H), 3.95 (s, 3H), 3.84 (s, 3H), 3.78 (s, 6H), 3.30 (s, 3H).

^{13}C NMR (126 MHz, Chloroform-*d*) δ 190.10, 172.64, 167.91, 153.45, 150.85, 144.84, 141.34, 131.19, 128.27, 125.85, 124.90, 124.08, 116.23, 108.94, 104.80, 83.07, 60.99, 56.25, 53.11, 27.03.

HRMS (ESI) calcd for $C_{23}H_{23}NO_8Na$ $[M+Na]^+$: 464.1316, found 464.1317.

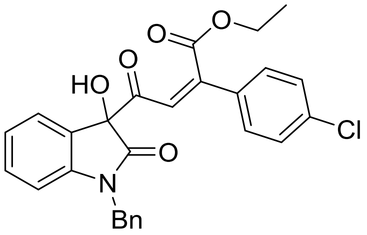

6v

ethyl

(*Z*)-4-(1-benzyl-3-hydroxy-2-oxoindolin-3-yl)-2-(4-chlorophenyl)-4-oxobut-2-enoate (6v)

White solid; 79% yield.

^{1}H NMR (400 MHz, Chloroform-*d*) δ 7.40 (dd, *J* = 7.6, 1.9 Hz, 2H), 7.36 – 7.27 (m, 4H), 7.24 (d, *J* = 8.6 Hz, 2H), 7.21 (d, *J* = 7.4 Hz, 1H), 7.11 – 7.03 (m, 3H), 6.94 (d, *J* = 7.9

Hz, 1H), 6.03 (s, 1H), 5.25 (d, J = 15.3 Hz, 1H), 5.07 (s, 1H), 4.63 (d, J = 15.3 Hz, 1H), 4.44 (qq, J = 6.9, 3.6 Hz, 2H), 1.39 (t, J = 7.2 Hz, 3H).

^{13}C NMR (126 MHz, Chloroform-*d*) δ 189.86, 172.55, 167.13, 149.81, 143.82, 137.76, 135.52, 131.18, 131.04, 129.33, 129.11, 128.44, 128.12, 127.86, 125.58, 124.90, 124.10, 116.13, 109.88, 83.18, 62.41, 44.52, 13.91.

HRMS (ESI) calcd for $C_{27}H_{22}NO_5ClNa$ $[M+Na]^+$: 498.1079, found 498.1079.

6. Transformations of product 6

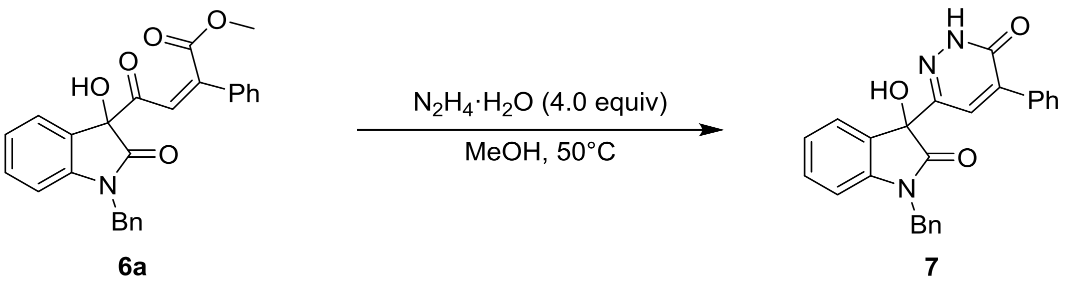

Supplementary Figure 4. Conversion of PCR product **6a** to **7**.

To a solution of **6a** (85.4 mg, 0.20 mmol) in MeOH (2.0 mL) was added NH_2NH_2•H_2O (40.0 mg, 0.80 mmol), and the reaction was stirred at 50°C under nitrogen atmosphere for 1 h (Supplementary Fig. 5) . After completion, the reaction mixture was extracted three times with ethyl acetate, washed with brine, dried over Na_2SO_4, filtered and concentrated in vacuum. The crude residue was purified by silica gel flash column chromatography (petroleum ether/ethyl acetate=10/1~2/1) to afford the desired product **7** as a white sold (80.1 mg, 98%).

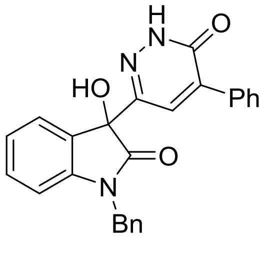

7

1-benzyl-3-hydroxy-3-(6-oxo-5-phenyl-1,6-dihydropyridazin-3-yl)indolin-2-one (7)

White solid; 98% yield.

^1H NMR (400 MHz, DMSO-d_6) δ 13.10 (s, 1H), 8.06 (s, 1H), 7.95 – 7.86 (m, 2H), 7.55 – 7.48 (m, 3H), 7.44 (d, J = 7.5 Hz, 1H), 7.41 – 7.33 (m, 4H), 7.32 (s, 1H), 7.30 – 7.22 (m, 2H), 7.03 (t, J = 7.5 Hz, 1H), 6.88 (d, J = 7.8 Hz, 1H), 4.98 (d, J = 15.9 Hz, 1H), 4.90 (d, J = 15.9 Hz, 1H).

^{13}C NMR (101 MHz, DMSO-d_6) δ 175.95, 160.26, 148.72, 142.84, 139.38, 136.41, 134.16, 131.42, 130.03, 129.12, 128.96, 128.84, 127.85, 127.66, 127.31, 125.21, 123.24, 110.05, 77.02, 43.16.

HRMS (ESI) calcd for $C_{25}H_{19}N_3O_3Na$ $[M+Na]^+$: 410.1499, found 410.1499.

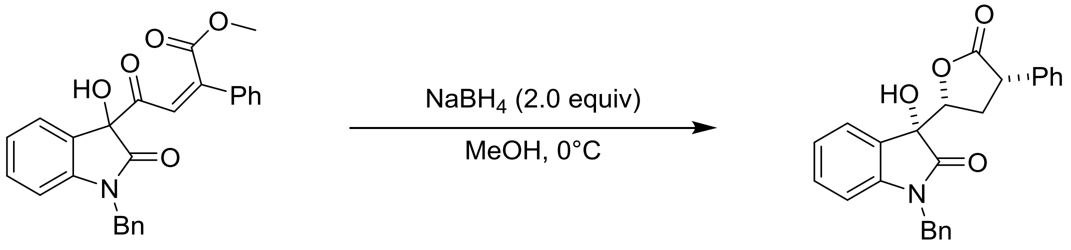

6a **8**

Supplementary Figure 5. Conversion of PCR product **6a** to **8**.

To a solution of **6a** (85.4 mg, 0.20 mmol) in MeOH (2.0 mL) was added $NaBH_4$ (15.1mg, 0.40 mmol), and the reaction was stirred at 0°C under nitrogen atmosphere for 0.5 h. After completion, the system was quenched by saturated aqueous solution of NH_4Cl. Then the reaction mixture was extracted three times with ethyl acetate, washed with brine, dried over Na_2SO_4, filtered and concentrated in vacuum. The crude residue was purified by silica gel flash column chromatography (petroleum ether/ethyl acetate = 10/1~4/1) to afford the desired product **8** as a white sold (Supplementary Fig. 5, 76.0 mg, 95%, > 95:5 dr.)

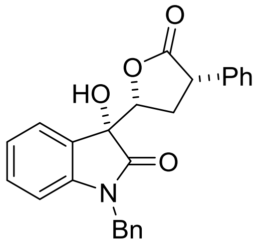

8

(*R)-1-benzyl-3-hydroxy-3-((2*R**,4*S**)-5-oxo-4-phenyltetrahydrofuran-2-yl)indolin**

-2-one (8)

White solid; 95% yield.

^{1}H NMR (500 MHz, DMSO-d_6) δ 7.52 (d, J = 7.4 Hz, 1H), 7.39 – 7.35 (m, 4H), 7.34 – 7.26 (m, 5H), 7.23 (d, J = 7.5 Hz, 2H), 7.09 (t, J = 7.5 Hz, 1H), 6.91 (d, J = 7.8 Hz, 1H), 6.81 (s, 1H), 4.96 (d, J = 15.9 Hz, 1H), 4.88 (d, J = 15.9 Hz, 1H), 4.79 (dd, J = 9.9, 6.3 Hz, 1H), 4.15 (dd, J = 12.1, 9.5 Hz, 1H), 2.74 (ddd, J = 12.8, 9.5, 6.3 Hz, 1H), 2.65 (td, J = 12.5, 10.0 Hz, 1H).

^{13}C NMR (126 MHz, DMSO-d_6) δ 176.61, 175.80, 143.07, 138.04, 136.46, 130.25, 129.08, 129.05, 128.83, 128.75, 127.88, 127.72, 127.68, 126.03, 122.94, 109.97, 79.58, 75.38, 45.90, 43.11, 31.48.

HRMS (ESI) calcd for $C_{25}H_{21}NO_4Na$ $[M+Na]^+$: 422.1363, found 422.1363.

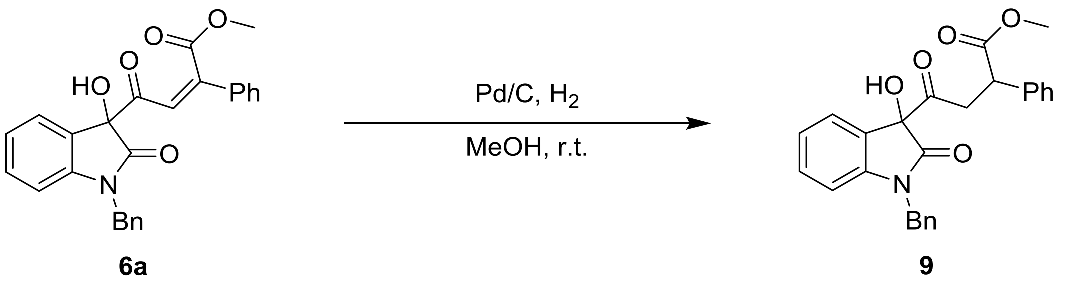

Supplementary Figure 6. Conversion of PCR product **6a** to **9**

To a solution of **6a** (85.4 mg, 0.20 mmol) in MeOH (4.0 mL) was added 10% Pd/C (8.5mg), and the reaction was stirred at room temperature under hydrogen atmosphere overnight. After completion, the reaction mixture was filtered, concentrated under vacuum and the residue was purified by silica gel flash column chromatography (petroleum ether/ethyl acetate = 10/1~4/1) to afford the desired product **9** as a white sold (Supplementary Fig. 6, 84.2 mg, 98%, 64:36 dr.)

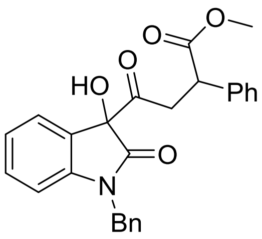

9

methyl 4-(1-benzyl-3-hydroxy-2-oxoindolin-3-yl)-4-oxo-2-phenylbutanoate (9)

White solid; 98% yield.

Data for major isomer:

^{1}H NMR (500 MHz, Chloroform-*d*) δ 7.34 (d, *J* = 8.7 Hz, 2H), 7.30 – 7.17 (m, 4H), 7.16 – 7.13 (m, 3H), 6.94 (dd, *J* = 6.6, 2.9 Hz, 2H), 6.87 (t, *J* = 7.5 Hz, 1H), 6.81 (d, *J* = 7.4 Hz, 1H), 6.74 (d, *J* = 7.8 Hz, 1H), 5.08 (d, *J* = 15.5 Hz, 1H), 4.79 (s, 1H), 4.70 (d, *J* = 15.5 Hz, 1H), 4.05 (dd, *J* = 8.5, 6.1 Hz, 1H), 3.56 (s, 3H), 3.07 (dd, *J* = 18.2, 8.7 Hz, 1H), 2.49 (dd, *J* = 18.2, 6.0 Hz, 1H).

^{13}C NMR (126 MHz, Chloroform-*d*) δ 201.45, 172.75, 172.55, 143.88, 137.10, 135.19, 130.88, 129.04, 128.86, 128.01, 127.70, 127.67, 127.57, 125.94, 124.16, 123.78, 110.07, 83.47, 52.52, 45.82, 44.49, 40.08.

HRMS (ESI) calcd for $C_{26}H_{23}NO_5Na$ $[M+Na]^+$: 452.1468, found 452.1466.

Data for minor isomer:

^{1}H NMR (500 MHz, Chloroform-*d*) δ 7.31 – 7.16 (m, 7H), 7.14 – 7.09 (m, 3H), 7.06 (t, *J* = 7.5 Hz, 1H), 6.98 (dd, *J* = 6.6, 2.9 Hz, 2H), 6.76 (d, *J* = 7.9 Hz, 1H), 4.93 (s, 1H), 4.93 (d, *J* = 15.5 Hz, 1H), 4.70 (d, *J* = 15.5 Hz, 1H), 4.05 (dd, *J* = 10.9, 3.8 Hz, 1H), 3.55 (s, 3H), 3.03 (dd, *J* = 18.4, 10.8 Hz, 1H), 2.46 (dd, *J* = 18.3, 3.8 Hz, 1H).

^{13}C NMR (126 MHz, Chloroform-*d*) δ 201.70, 173.15, 172.93, 143.70, 137.14, 134.97, 131.01, 128.95, 128.90, 127.98, 127.77, 127.57, 127.37, 126.33, 124.73, 123.96, 110.10, 83.24, 52.52, 45.61, 44.33, 39.89.

HRMS (ESI) calcd for $C_{26}H_{23}NO_5Na$ $[M+Na]^+$: 452.1468, found 452.1466.

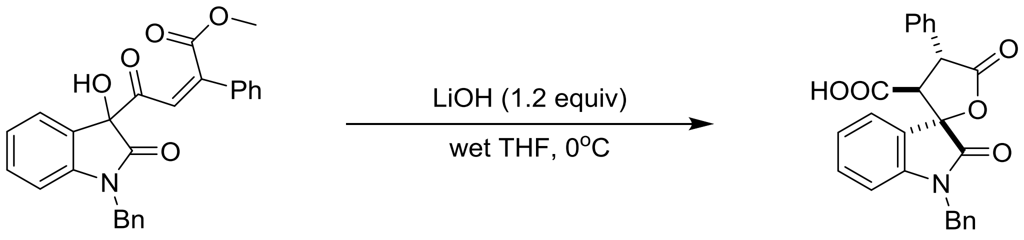

6a **10a**

Supplementary Figure 7. Conversion of PCR product **6a** to **10a**

To a clean round bottomed flask was added **6a** (85.4 mg, 0.20 mmol) and THF (2.0

mL). The mixture was cooled in an ice bath and then a solution of 1.2 M aqueous LiOH (0.2 mL) was added slowly. The reaction system stirred at 0°C for 30 min. After completion, 1M HCl was added to render the solution acidic (pH =1~2), and the mixture was extracted three times with ethyl acetate, washed with brine, dried over Na_2SO_4, filtered and concentrated in vacuum. The crude residue was purified by silica gel flash column chromatography (CH_2Cl_2/MeOH=50/1~10/1) to afford the desired product **10a** as a white sold (Supplementary Fig. 8, 58.0 mg, 70%, > 95:5 dr)

General procedures for the synthesis of 10 in a sequential fashion and a one-pot fashion

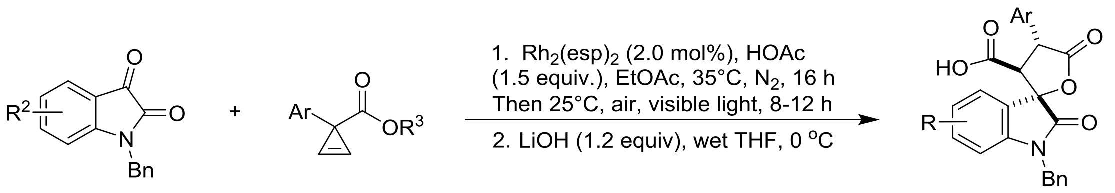

4 **5** **10**

Supplementary Figure 8. Synthesis of **10** in a sequential fashion.

A sequential fashion procedure: As shown in Supplementary Fig. 8, To a solution of isatin **4** (0.20 mmol, 1.0 equiv.), $Rh_2(esp)_2$ (3.0 mg, 2 mol%) and acetic acid (0.30 mmol, 1.5 equiv.) in ethyl acetate (3 mL) was added Cycloprop-2-ene-1-ester **5** (0.30 mmol, 1.5 equiv.). The reaction was stirred at 35°C under nitrogen atmosphere for 16 h, then the reaction was stirred at 25°C under air atmosphere and visible light irradiation (12 W white LEDs or sunlight) for 8-12 h. The reaction mixture was filtered, concentrated under vacuum. To a clean round bottomed flask was added the residue and THF (2.0 mL). The mixture was cooled in an ice bath and then a solution of 1.2 M aqueous LiOH (0.2 mL) was added slowly. The reaction system stirred at 0°C for 15~40 min. After completion, 1M HCl was added to render the solution acidic (pH =1~2), and the mixture was extracted three times with ethyl acetate, washed with brine, dried over Na_2SO_4, filtered and concentrated in vacuum. The crude residue was purified by silica gel flash column chromatography (CH_2Cl_2/MeOH=50/1~10/1) to

afford the desired product **10** as a white solid.

A one-pot fashion: As shown in Supplementary Fig. 9., to a clean round bottomed flask was added **6a** (85.4 mg, 0.20 mmol) and EtOAc (2.0 mL). The mixture was cooled in an ice bath and then a solution of 1.2 M aqueous LiOH (0.2 mL) was added slowly. The reaction system stirred at 0°C for 30 min. After completion, 1M HCl was added to render the solution acidic (pH =1~2), and the mixture was extracted three times with ethyl acetate, washed with brine, dried over Na_2SO_4, filtered and concentrated in vacuum. The crude residue was purified by silica gel flash column chromatography (petroleum ether/ethyl acetate=10/1~4/1) to afford the desired product **12** as a white sold (81.2 mg, 95%).

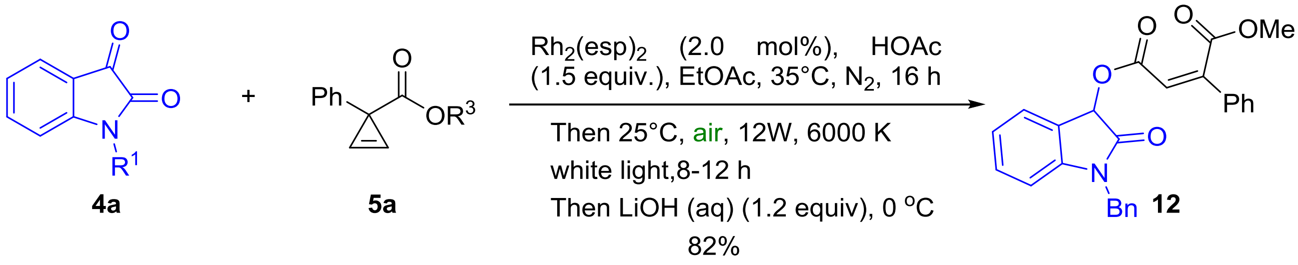

Supplementary Figure 9. Synthesis of **12** in a one-pot fashion

7. Data of product 10

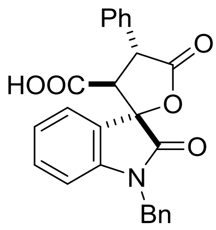

10a

(2*S,3*S**,4*R**)-1'-benzyl-2',5-dioxo-4-phenyl-4,5-dihydro-3H-spiro[furan-2,3'-indoline]-3-carboxylic acid (10a)**

White solid; 56% yield; > 95:5 dr.

^{1}H NMR (500 MHz, DMSO-d_6) δ 13.33 (br.s, 1H), 7.96 (d, J = 7.4 Hz, 1H), 7.56 (d, J = 7.5 Hz, 2H), 7.43 (t, J = 7.5 Hz, 2H), 7.41 – 7.32 (m, 6H), 7.28 (t, J = 7.1 Hz, 1H), 7.18 (t, J = 7.5 Hz, 1H), 6.91 (d, J = 7.9 Hz, 1H), 5.05 (d, J = 16.0 Hz, 1H), 4.84 (d, J = 16.0

Hz, 1H), 4.80 (d, *J* = 12.3 Hz, 1H), 4.41 (d, *J* = 12.2 Hz, 1H).

^{13}C NMR (126 MHz, DMSO-*d*$_6$) δ 174.94, 173.40, 169.78, 144.40, 136.41, 135.98, 131.92, 129.73, 129.17, 128.98, 128.22, 127.97, 127.58, 126.09, 125.06, 123.72, 110.52, 81.28, 54.64, 48.70, 43.48.

HRMS (ESI) calcd for $C_{25}H_{19}NO_5Na$ [M+Na]$^+$: 436.1155, found 436.1155.

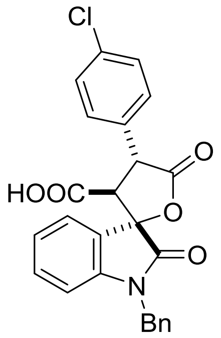

10b

(2*S,3*S**,4*R**)-1'-benzyl-4-(4-chlorophenyl)-2',5-dioxo-4,5-dihydro-3H-spiro[furan-2,3'-indoline]-3-carboxylic acid (10b)**

White solid; 57% yield; > 95:5 dr.

^{1}H NMR (400 MHz, DMSO-*d*$_6$) δ 13.35 (br.s, 1H), 7.97 (d, *J* = 7.4 Hz, 1H), 7.63 (d, *J* = 8.2 Hz, 2H), 7.49 (d, *J* = 8.2 Hz, 2H), 7.45 – 7.22 (m, 6H), 7.17 (t, *J* = 7.5 Hz, 1H), 6.91 (d, *J* = 7.8 Hz, 1H), 5.04 (d, *J* = 16.0 Hz, 1H), 4.94 – 4.75 (m, 2H), 4.44 (d, *J* = 12.4 Hz, 1H).

^{13}C NMR (126 MHz, DMSO-*d*$_6$) δ 174.59, 173.35, 169.72, 144.41, 135.97, 135.29, 133.02, 131.95, 131.74, 129.16, 128.89, 127.97, 127.58, 126.19, 124.96, 123.68, 110.52, 81.30, 54.29, 48.04, 43.47.

HRMS (ESI) calcd for $C_{25}H_{18}ClNO_5Na$ [M+Na]$^+$: 470.0766, found 470.0766.

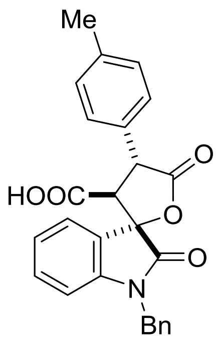

10c

(2*S,3*S**,4*R**)-1'-benzyl-2',5-dioxo-4-(p-tolyl)-4,5-dihydro-3H-spiro[furan-2,3'-indoline]-3-carboxylic acid (10c)**

White solid; 56% yield; > 95:5 dr.

^{1}H NMR (400 MHz, DMSO-d_6) δ 13.29 (br.s, 1H), 7.94 (d, *J* = 7.4 Hz, 1H), 7.43 (d, *J* = 7.6 Hz, 2H), 7.41 – 7.24 (m, 6H), 7.24 – 7.02 (m, 3H), 6.90 (d, *J* = 7.9 Hz, 1H), 5.04 (d, *J* = 16.0 Hz, 1H), 4.83 (d, *J* = 16.0 Hz, 1H), 4.74 (d, *J* = 12.1 Hz, 1H), 4.34 (d, *J* = 12.1 Hz, 1H), 2.33 (s, 3H).

^{13}C NMR (126 MHz, DMSO-d_6) δ 175.06, 173.41, 169.79, 144.38, 137.47, 135.99, 133.43, 131.90, 129.55, 129.16, 127.96, 127.57, 126.05, 125.09, 123.70, 110.50, 81.22, 54.76, 48.37, 43.46, 21.18.

HRMS (ESI) calcd for $C_{26}H_{21}NO_5Na$ $[M+Na]^+$: 450.1312, found 450.1312.

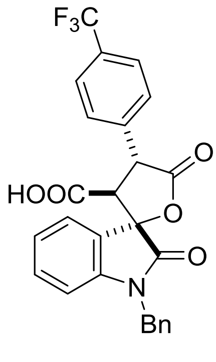

10d

(2*S,3*S**,4*R**)-1'-benzyl-2',5-dioxo-4-(4-(trifluoromethyl)phenyl)-4,5-dihydro-3H-spiro[furan-2,3'-indoline]-3-carboxylic acid (10d)**

White solid; 52% yield; > 95:5 dr.

^{1}H NMR (400 MHz, DMSO-d_6) δ 13.40 (br.s, 1H), 8.02 (d, J = 6.8 Hz, 2H), 7.92 (d, J = 7.7 Hz, 1H), 7.74 (d, J = 7.8 Hz, 1H), 7.67 (t, J = 7.7 Hz, 1H), 7.55 – 7.23 (m, 6H), 7.18 (t, J = 7.5 Hz, 1H), 6.91 (d, J = 7.9 Hz, 1H), 5.15 – 4.95 (m, 2H), 4.85 (d, J = 16.0 Hz, 1H), 4.57 (d, J = 12.4 Hz, 1H).

^{13}C NMR (101 MHz, DMSO-d_6) δ 174.46, 173.37, 169.71, 144.45, 137.47, 136.00, 134.20, 131.96, 129.91, 129.68 (q, J = 31.6 Hz), 129.17, 127.97, 127.60, 126.60 (d, J = 4.1 Hz), 126.38, 125.04 (d, J = 3.4 Hz), 124.92, 124.70 (q, J = 272.3 Hz), 123.62, 110.49, 81.40, 54.00, 48.31, 43.49.

^{19}F NMR (376 MHz, DMSO-d_6) δ -60.74.

HRMS (ESI) calcd for $C_{26}H_{18}F_3NO_5Na$ $[M+Na]^+$: 504.1029, found 504.1028.

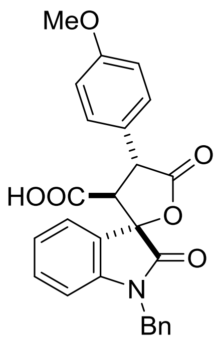

10e

(2*S,3*S**,4*R**)-1'-benzyl-4-(4-methoxyphenyl)-2',5-dioxo-4,5-dihydro-3H-spiro[furan-2,3'-indoline]-3-carboxylic acid (10e)**

White solid; 55% yield; > 95:5 dr.

^{1}H NMR (400 MHz, DMSO-d_6) δ 13.26 (br.s, 1H), 7.94 (d, J = 7.4 Hz, 1H), 7.47 (d, J = 8.2 Hz, 2H), 7.43 – 7.24 (m, 6H), 7.17 (t, J = 7.6 Hz, 1H), 6.97 (d, J = 8.2 Hz, 2H), 6.90 (d, J = 7.9 Hz, 1H), 5.04 (d, J = 15.7 Hz, 1H), 4.83 (d, J = 16.1 Hz, 1H), 4.73 (d, J = 12.0 Hz, 1H), 4.31 (d, J = 12.1 Hz, 1H), 3.78 (s, 3H).

^{13}C NMR (126 MHz, DMSO-d_6) δ 175.24, 173.43, 169.83, 159.28, 144.38, 136.00, 131.88, 130.86, 129.16, 128.35, 127.96, 127.58, 126.05, 125.13, 123.69, 114.37, 110.49, 81.18, 55.61, 54.89, 48.00, 43.46.

HRMS (ESI) calcd for $C_{26}H_{21}NO_6Na$ $[M+Na]^+$: 466.1261, found 466.1262.

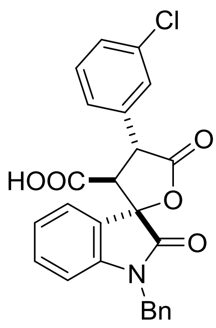

10f

(2*S,3*S**,4*R**)-1'-benzyl-4-(3-chlorophenyl)-2',5-dioxo-4,5-dihydro-3H-spiro[furan-2,3'-indoline]-3-carboxylic acid (10f)**

White solid; 57% yield; > 95:5 dr.

^{1}H NMR (400 MHz, DMSO-d_6) δ 13.37 (br.s, 1H), 8.01 (d, *J* = 7.5 Hz, 1H), 7.75 (s, 1H), 7.55 (d, *J* = 6.7 Hz, 1H), 7.53 – 7.22 (m, 8H), 7.17 (t, *J* = 7.6 Hz, 1H), 6.91 (d, *J* = 7.9 Hz, 1H), 5.04 (d, *J* = 16.1 Hz, 1H), 4.89 (d, *J* = 12.5 Hz, 1H), 4.84 (d, *J* = 16.0 Hz, 1H), 4.49 (d, *J* = 12.5 Hz, 1H).

^{13}C NMR (126 MHz, DMSO-d_6) δ 174.44, 173.36, 169.69, 144.42, 138.54, 135.98, 133.59, 131.93, 130.70, 129.50, 129.16, 128.84, 128.28, 127.96, 127.58, 126.32, 124.93, 123.64, 110.48, 81.35, 54.10, 48.26, 43.48.

HRMS (ESI) calcd for $C_{25}H_{18}ClNO_5Na$ $[M+Na]^+$: 470.0766, found 470.0766.

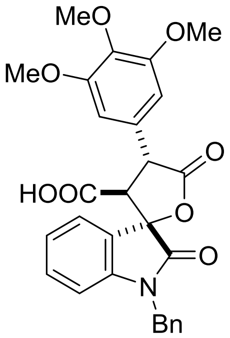

10g

(2*S,3*S**,4*R**)-1'-benzyl-2',5-dioxo-4-(3,4,5-trimethoxyphenyl)-4,5-dihydro-3H-spiro[furan-2,3'-indoline]-3-carboxylic acid (10g)**

White solid; 49% yield; 86:14 dr.

Data for major isomer:

^{1}H NMR (400 MHz, DMSO-d_6) δ 13.79 (br.s, 1H), 7.91 (d, *J* = 7.4 Hz, 1H), 7.48 – 7.22 (m, 6H), 7.16 (t, *J* = 7.6 Hz, 1H), 6.86 (d, *J* = 8.1 Hz, 1H), 6.82 – 6.60 (m, 2H), 5.01 (d, *J* = 15.7 Hz, 1H), 4.82 (d, *J* = 16.0 Hz, 1H), 4.74 (d, *J* = 12.3 Hz, 1H), 4.28 (d, *J* = 12.4 Hz, 1H), 3.82 (s, 6H), 3.68 (s, 3H).

^{13}C NMR (126 MHz, DMSO-d_6) δ 175.36, 173.74, 169.81, 153.28, 144.50, 137.39, 136.10, 131.64, 129.12, 127.87, 127.61, 127.47, 126.01, 123.40, 110.30, 107.13, 106.66, 81.64, 60.40, 56.53, 56.30, 49.25, 43.42.

HRMS (ESI) calcd for $C_{28}H_{25}NO_8Na$ $[M+Na]^+$: 526.1472, found 526.1472.

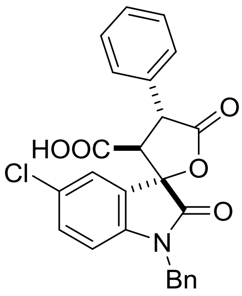

10h

(2*S,3*S**,4*R**)-1'-benzyl-5'-chloro-2',5-dioxo-4-phenyl-4,5-dihydro-3H-spiro[furan-2,3'-indoline]-3-carboxylic acid (10h)**

White solid; 37% yield; > 95:5 dr.

^{1}H NMR (400 MHz, DMSO-d_6) δ 13.18 (br.s, 1H), 8.22 (d, *J* = 2.2 Hz, 1H), 7.59 (d, *J* = 7.5 Hz, 2H), 7.50 – 7.26 (m, 9H), 6.92 (d, *J* = 8.4 Hz, 1H), 5.05 (d, *J* = 16.1 Hz, 1H), 4.84 (d, *J* = 16.1 Hz, 1H), 4.78 (d, *J* = 12.4 Hz, 1H), 4.47 (d, *J* = 12.4 Hz, 1H).

^{13}C NMR (101 MHz, DMSO-d_6) δ 174.76, 173.26, 169.75, 143.30, 136.19, 135.66, 131.69, 129.88, 129.21, 128.87, 128.21, 128.05, 127.56, 127.00, 126.59, 112.01, 80.94, 54.50, 48.57, 43.57.

HRMS (ESI) calcd for $C_{25}H_{18}ClNO_5Na$ $[M+Na]^+$: 470.0766, found 470.0765.

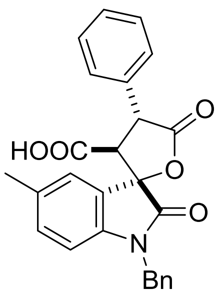

10i

(2*S,3*S**,4*R**)-1'-benzyl-5'-methyl-2',5-dioxo-4-phenyl-4,5-dihydro-3H-spiro[furan-2,3'-indoline]-3-carboxylic acid (10i)**

White solid; 60% yield; > 95:5 dr.

^{1}H NMR (400 MHz, DMSO-d_6) δ 13.33 (br.s, 1H), 7.80 (s, 1H), 7.56 (d, *J* = 7.5 Hz, 2H), 7.43 (t, *J* = 7.4 Hz, 2H), 7.40 – 7.23 (m, 6H), 7.17 (d, *J* = 8.0 Hz, 1H), 6.78 (d, *J* = 8.0 Hz, 1H), 5.02 (d, *J* = 16.0 Hz, 1H), 4.87 – 4.73 (m, 2H), 4.36 (d, *J* = 12.3 Hz, 1H), 2.31 (s, 3H).

^{13}C NMR (126 MHz, DMSO-d_6) δ 174.97, 173.31, 169.77, 141.98, 136.49, 136.05, 133.05, 131.97, 129.71, 129.13, 128.98, 128.21, 127.91, 127.54, 126.64, 125.04, 110.26, 81.44, 54.70, 48.76, 43.46, 21.01.

HRMS (ESI) calcd for $C_{26}H_{21}NO_5Na$ $[M+Na]^+$: 450.1312, found 450.1312.

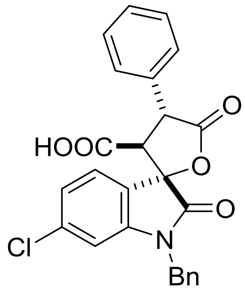

10j

(2*S,3*S**,4*R**)-1'-benzyl-6'-chloro-2',5-dioxo-4-phenyl-4,5-dihydro-3H-spiro[furan-2,3'-indoline]-3-carboxylic acid (10j)**

White solid; 50% yield; > 95:5 dr.

^{1}H NMR (400 MHz, DMSO-d_6) δ 13.37 (br.s, 1H), 8.02 (d, J = 8.0 Hz, 1H), 7.56 (d, J = 7.4 Hz, 2H), 7.45 – 7.23 (m, 9H), 7.06 (s, 1H), 5.06 (d, J = 16.1 Hz, 1H), 4.87 (d, J = 16.2 Hz, 1H), 4.78 (d, J = 12.3 Hz, 1H), 4.43 (d, J = 12.5 Hz, 1H).

^{13}C NMR (126 MHz, DMSO-d_6) δ 174.79, 173.57, 169.76, 145.91, 136.39, 136.26, 135.67, 129.78, 129.23, 128.93, 128.22, 128.08, 127.80, 127.60, 123.94, 123.47, 110.87, 80.71, 54.54, 48.56, 43.49.

HRMS (ESI) calcd for $C_{25}H_{18}ClNO_5Na$ $[M+Na]^+$: 470.0766, found 470.0766.

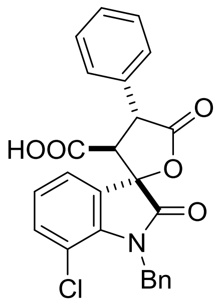

10k

(2*S,3*S**,4*R**)-1'-benzyl-7'-chloro-2',5-dioxo-4-phenyl-4,5-dihydro-3H-spiro[furan-2,3'-indoline]-3-carboxylic acid (10k)**

White solid; 35% yield; > 95:5 dr.

^{1}H NMR (400 MHz, DMSO-d_6) δ 13.39 (br.s, 1H), 8.02 (d, J = 7.4 Hz, 1H), 7.57 (d, J = 7.4 Hz, 2H), 7.53 – 7.00 (m, 10H), 5.28 (d, J = 17.3 Hz, 1H), 5.22 (d, J = 16.6 Hz, 1H), 4.78 (d, J = 12.3 Hz, 1H), 4.44 (d, J = 12.6 Hz, 1H).

^{13}C NMR (101 MHz, DMSO-d_6) δ 174.73, 174.38, 169.71, 140.35, 137.58, 136.26, 134.13, 129.78, 129.07, 128.96, 128.25, 127.52, 126.30, 125.43, 125.30, 115.22, 80.43, 54.97, 48.60, 45.18.

HRMS (ESI) calcd for $C_{25}H_{18}ClNO_5Na$ $[M+Na]^+$: 470.0766, found 470.0768.

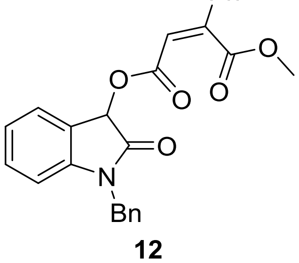

12

4-(1-benzyl-2-oxoindolin-3-yl) 1-methyl 2-phenylmaleate (12)

White solid; 82% yield.

^{1}H NMR (500 MHz, Chloroform-*d*) δ 7.51 – 7.48 (m, 2H), 7.45 – 7.38 (m, 4H), 7.36 – 7.31 (m, 4H), 7.28 (p, *J* = 4.1 Hz, 1H), 7.23 (t, *J* = 7.8 Hz, 1H), 7.03 (t, *J* = 7.6 Hz, 1H), 6.72 (d, *J* = 7.8 Hz, 1H), 6.44 (s, 1H), 6.18 (s, 1H), 4.97 (d, *J* = 15.7 Hz, 1H), 4.88 (d, *J* = 15.6 Hz, 1H), 3.91 (s, 3H).

^{13}C NMR (126 MHz, Chloroform-*d*) δ 171.95, 167.99, 164.27, 150.21, 143.67, 135.18, 133.02, 130.95, 130.40, 129.12, 128.88, 127.83, 127.36, 126.92, 126.15, 124.12, 123.24, 116.10, 109.59, 70.13, 52.81, 44.00.

HRMS (ESI) calcd for $C_{26}H_{21}NO_5Na$ $[M+Na]^+$: 450.1312, found 450.1311.

8. Evaluation of the function of $Rh_2(OAc)_4$ and $Rh_2(esp)_2$ as photosensitizer for generation of singlet oxygen

The substrate used was 1,3-diphenylisobenzofuran (DPBF), which is known to have a high detection sensitivity for singlet oxygen detection. Firstly, DPBF1 could be oxidized smoothly under red light or blue light, implicating dirhodium catalysts can produce ^{1}O2 via LMCT and MMCT modes (Supplementary Fig. 10). Additionally, DPBF **1** could be oxidized to 1,2-dibenzoylbenzene **3** under the condition of using dirhodium catalysts as photosenstizers, 12 W LED as light source, air as oxidant and $CDCl_3$ as solvent (Supplementary Fig. 11, condition a and b). Lack of dirhodium catalyst (Supplementary Fig. 11, condition c), white light (Supplementary Fig. 11, condition d), or oxygen (Supplementary Fig. 11, condition e) leads to the inhibition of the oxidation. To figure out whether the MMCT transition contributes to the photocatalytic activity, 5W, 640-650 nm red light was used and the DPBF could be smoothly oxidized. Meanwhile, we tested 5W, 440-450 nm blue light and found it could finish the oxidation of DPBF as well (Supplementary Fig.11). These data demonstrate the $[Rh_2]$ catalyst adopts both MMCT and MLCT transition to perform the photocatalytic oxidation. Considering the harmlessness of white light, the white LED will be used in the following experiments.

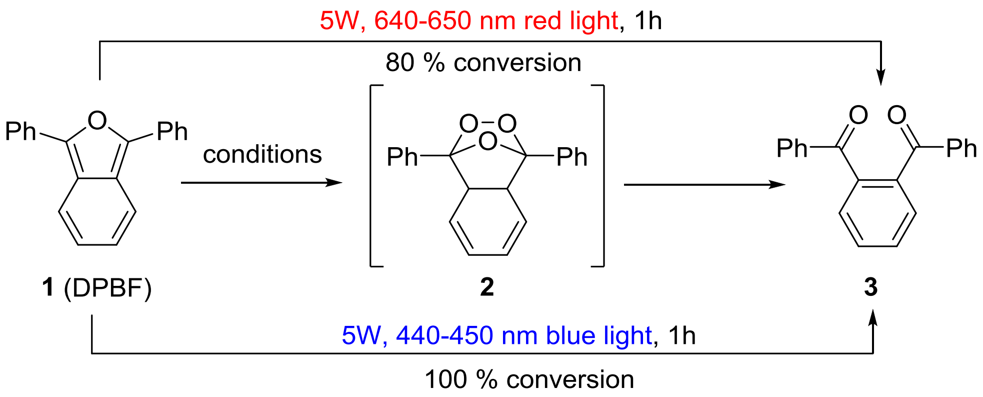

Supplementary Figure 10. Photooxidation of DPBF **1** under red light or blue light

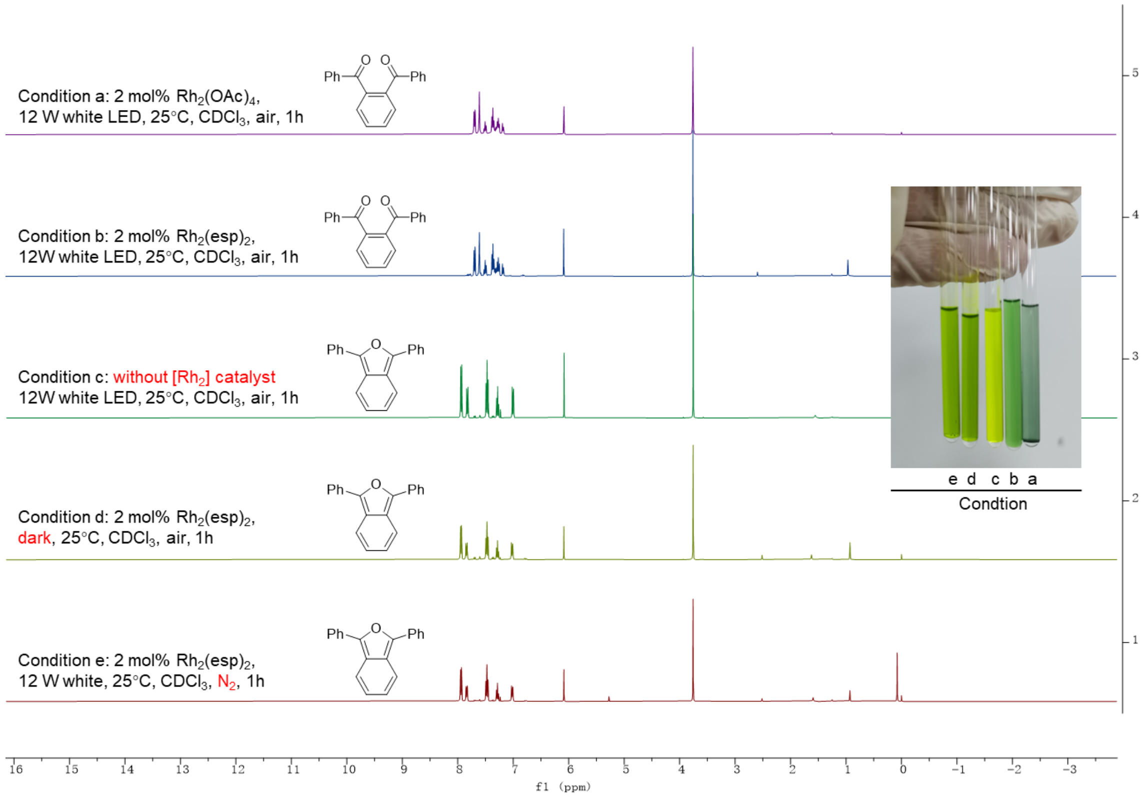

Supplementary Figure 11. The detection of singlet oxygen by 1,3-Diphenylisobenzofuran (DPBF) 1.

Supplementary Table 1. The detection of singlet oxygen by 1,3-Diphenylisobenzofuran (DPBF)[a].

Condition	$[Rh_2]$ catalyst	Conversion yield/%[b]
A	$Rh_2(OAc)_4$	99 (68[c])

B	$Rh_2(esp)_2$	99 (61[c])
C	None	Trace
D	$Rh_2(esp)_2$ (dark reaction)	Trace
E	$Rh_2(esp)_2$[c] (N_2)	not detected

[a]Reaction conditions: **1** (0.1 mmol), [Rh_2] catalyst, $CDCl_3$ (1 mL), 12W white LED.
[b]The conversion yields of **1** were determined by crude ^1H NMR using 1,3,5-trimethoxylbenzene as the internal standard.
[c]Yields of **3**.

1,2-phenylenebis(phenylmethanone) (3)

^1H NMR (400 MHz, Chloroform-*d*) δ 7.88 (d, J = 7.4 Hz, 4H), 7.79 (s, 4H), 7.69 (t, J = 7.1 Hz, 2H), 7.55 (t, J = 7.4 Hz, 4H).

9. Supplementary optimization of reactions

In the study of optimization of reactions, we found a new by-product **S4**, which was the hydrolyzed product of the intermediate **6a-Int**. By using $Rh_2(esp)_2$ as a catalyst, these strong acids such as *rac*-BNDHP and *p*-TSA caused the formation of by-product **S4**.

Using $Rh_2(TFA)_4$ instead of $Rh_2(esp)_2$ as a catalyst and HOAc as Brønsted acid also led to the formation of **S4** (Supplementary Table 2).

Supplementary Table 2. Screening of metal catalysts and Acid.

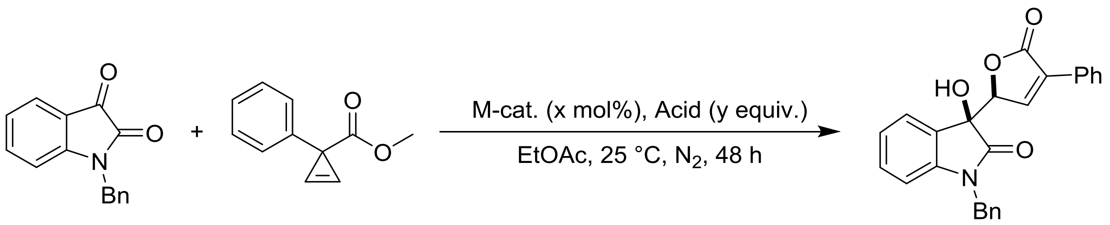

4a **5a** **S4**

Entry	M-cat. (x mol%)	Acid (y equiv.)	Yield/%	dr[a]
1	$Rh_2(esp)_2$ (2)	(±)-BNDHP (1.5)	70	80:20
2	$Rh_2(esp)_2$ (2)	*p*-TSA (1.5)	68	75:25

3	$Rh_2(TFA)_4$ (2)	HOAc (1.5)	53	78:22

[a]The dr values were determined by ^1H NMR spectroscopy of the crude reaction mixture.

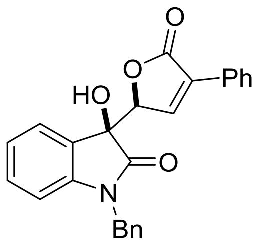

S4

(*S)-1-benzyl-3-hydroxy-3-((*S**)-5-oxo-4-phenyl-2,5-dihydrofuran-2-yl)indolin-2-one (S4)**

White solid.

^1H NMR (500 MHz, DMSO-d_6) δ 8.36 (d, *J* = 1.8 Hz, 1H), 7.91 (d, *J* = 6.6 Hz, 2H), 7.48 – 7.40 (m, 3H), 7.37 (d, *J* = 7.4 Hz, 2H), 7.31 (t, *J* = 7.4 Hz, 2H), 7.27 (d, *J* = 7.0 Hz, 1H), 7.22 (t, *J* = 7.7 Hz, 1H), 7.15 (d, *J* = 7.3 Hz, 1H), 7.07 (s, 1H), 6.92 (t, *J* = 7.5 Hz, 1H), 6.85 (d, *J* = 7.8 Hz, 1H), 5.52 (d, *J* = 1.9 Hz, 1H), 5.00 (d, *J* = 15.9 Hz, 1H), 4.89 (d, *J* = 15.9 Hz, 1H).

^{13}C NMR (126 MHz, DMSO-d_6) δ 175.46, 170.66, 147.31, 143.52, 136.33, 132.25, 130.62, 129.82, 129.71, 129.15, 129.02, 127.84, 127.56, 127.33, 126.38, 125.87, 122.51, 110.00, 82.02, 76.73, 43.13.

HRMS (ESI) calcd for $C_{25}H_{19}NO_4Na$ $[M+Na]^+$: 420.1206, found: 420.1208.

10. Control experiments and the inert substrates of the PCR

First, we used pure **7a** to perform oxidation under standard conditions and provided **6a** in 83% yield, implicating **6a** is oxidized from **7a** (Supplementary Fig.12, eq.1 condition A). Eliminating one of the conditions including visible light, $Rh_2(esp)_2$ and oxygen caused significant reduction in the yield (Supplementary Fig.12, eq. 1, condition B, C, and D). Secondly, we generated furan **11** from **5a** *in situ* and added **4a** subsequently. The reaction gave no intermediate **6a-Int** (Supplementary Fig.12, eq. 2). Lastly, we tested a group of four chiral dirhodium catalysts including Rh_2(S-PTTL)$_4$,

Rh_2(S-PTPA)$_4$, Rh_2(R-BPTTL)$_4$ and Rh_2(R-BTPCB)$_4$, and only detected racemic product **6a** (Supplementary Fig.12, Eq. 3), indicating [Rh_2] catalyst is not binding with the zwitterion when reacting with **4a**.

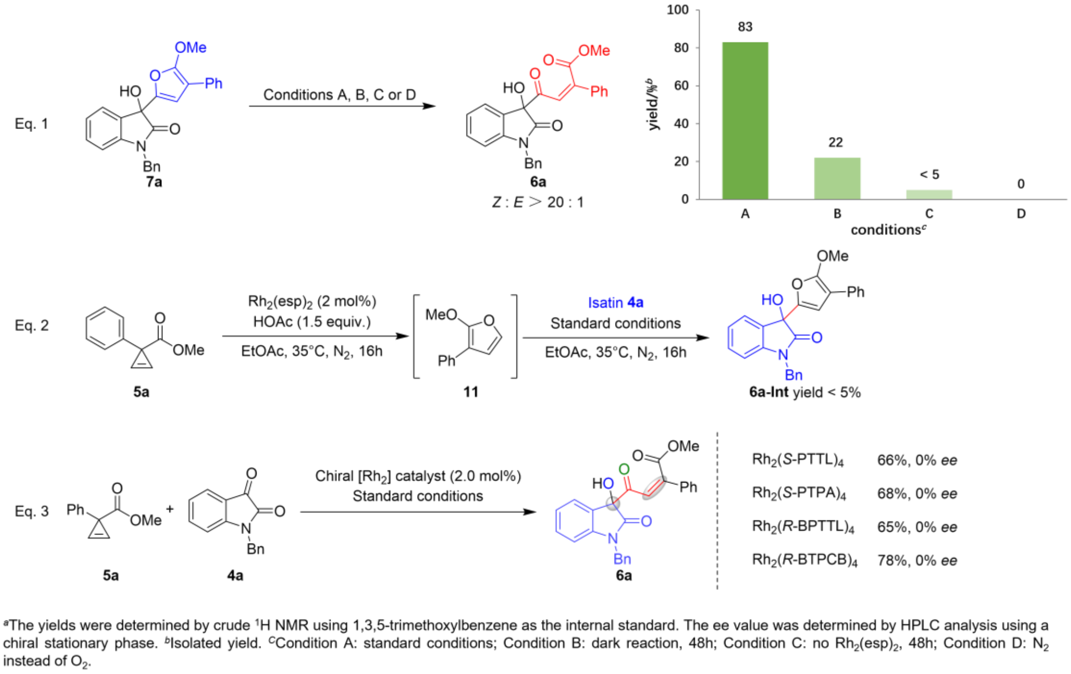

Supplementary Figure 12. Control experiments and asymmetric catalysis temptation of the PCR of **4a** and **5a**[a].

[a]The yields were determined by crude ^{1}H NMR using 1,3,5-trimethoxylbenzene as the internal standard. The ee value was determined by HPLC analysis using a chiral stationary phase. [b]Isolated yield. [c]Condition A: standard conditions; Condition B: dark reaction, 48h; Condition C: no $Rh_2(esp)_2$, 48h; Condition D: N_2 instead of O_2.

We generated furan **11** from **5a** *in situ* and added isatin **4a** subsequently to figure out the generation of intermediate **6a-Int** is through Friedel-Crafts reaction of furan **11** with istain **4a** or through the capture of transient zwitterion by **4a**. The reaction gave no intermediate **6a-Int** (Supplementary Fig.13, Eq. 2b), while the yield was up to 98% using the one-pot method (Supplementary Fig.13, Eq. 2a), excluding the pathway of the Friedel-Crafts reaction.

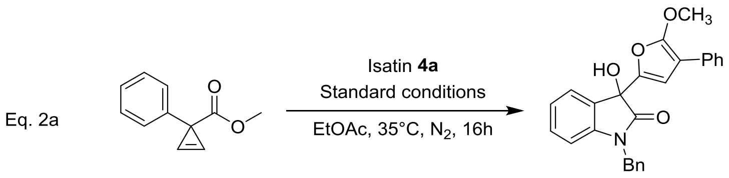

5a **6a-Int** 98%

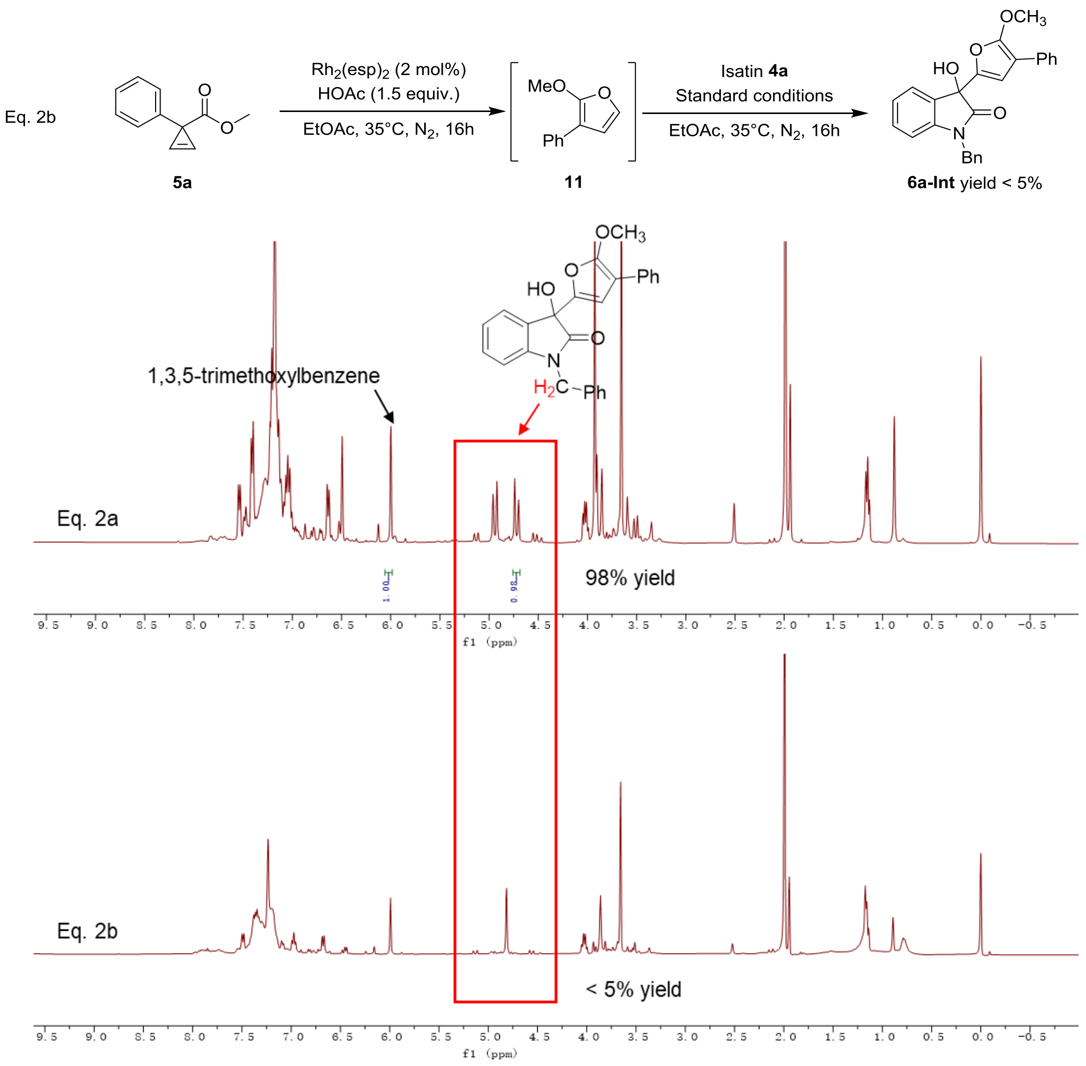

Supplementary Figure 13. Control experiment eq.2.

Inert substrates of the PCR：As shown in Supplementary Fig. 14, the reaction with ethyl dicarboxylate cyclopropene could afford the furan intermediate, but it failed to be oxidized to the corresponding 1,4-dicarbonyl Z-alkene. We speculate the electron-deficient carboxyl group lowers the electron density of the furan ring and makes it harder to oxidize. Upon alkyl-carboxylate cyclopropene, we didn't detect the desired benzyl-carboxylate cyclopropene according to the reported procedure but observed the furan product. With the information in mind, we tried to generate the unstable benzyl-carboxylate cyclopropene *in situ* by running a three-component reaction of benzyldiazoacetate, alkyne, and isatin. However, what we isolated is the product of the three-component reaction of benzyldiazoacetate, water, and isatin.

Compared to its aryl-conterparters, we assume alkyl-carboxylate cyclopropenes losing the stablization of carbene or zewitterion intermediates by the resonance with aryl groups become less stable and cann’t be captured by the isatin.

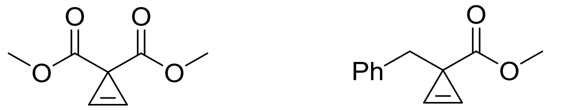

5w **5x (generated *in situ*)**

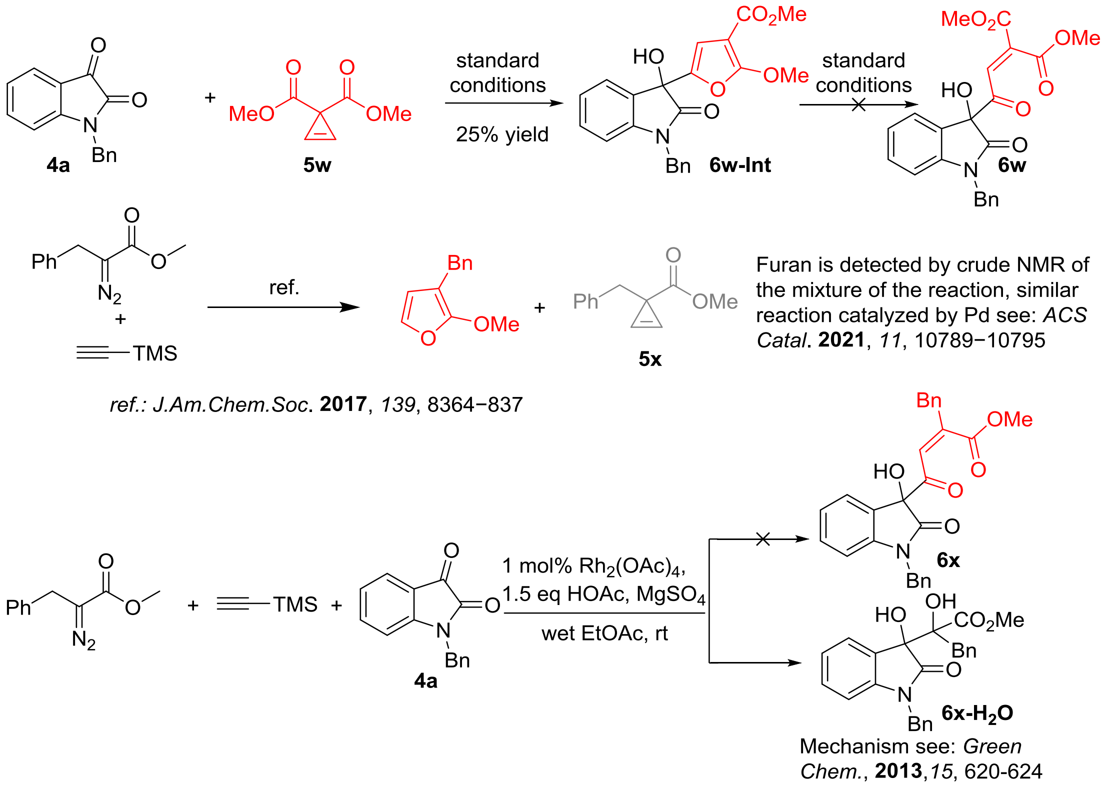

Supplementary Figure 14. Investigation of alkyldiazo-derived cyclopropene and carboxylatediazo-derived cyclopropane in the PCR reaction.

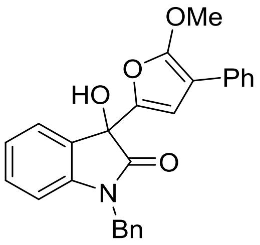

6a-Int

1-benzyl-3-hydroxy-3-(5-methoxy-4-phenylfuran-2-yl)indolin-2-one (6a-Int)

White solid; **6a-Int** underwent dehydration easily at room temperature, then turned into red solid.

^{1}H NMR (400 MHz, DMSO-d_6) δ 7.52 (dd, J = 10.0, 7.3 Hz, 3H), 7.38 – 7.31 (m, 6H), 7.28 (t, J = 7.7 Hz, 2H), 7.17 (t, J = 7.4 Hz, 1H), 7.09 (t, J = 7.5 Hz, 1H), 7.01 (s, 1H), 6.95 (d, J = 7.8 Hz, 1H), 6.65 (s, 1H), 4.99 (d, J = 15.8 Hz, 1H), 4.91 (d, J = 15.8 Hz, 1H), 3.96 (s, 3H).

^{13}C NMR (101 MHz, DMSO-d_6) δ 174.75, 155.99, 142.99, 142.41, 136.62, 131.98, 130.12, 130.09, 129.12, 127.95, 127.64, 126.13, 125.70, 125.33, 123.31, 110.02, 108.95, 99.62, 73.39, 59.46, 43.13.

HRMS (ESI) calcd for $C_{26}H_{21}NO_4Na$ $[M+Na]^+$: 434.1363, found 434.1363.

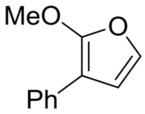

11

2-methoxy-3-phenylfuran (11)

Light yellow oil.

^{1}H NMR (500 MHz, Chloroform-d) δ 7.50 (d, J = 7.7 Hz, 2H), 7.27 (t, J = 7.7 Hz, 2H), 7.09 (t, J = 7.5 Hz, 1H), 6.89 (d, J = 2.5 Hz, 1H), 6.54 (d, J = 2.5 Hz, 1H), 3.94 (s, 3H).

^{13}C NMR (126 MHz, Chloroform-d) δ 155.03, 131.92, 131.43, 127.48, 124.54, 124.40, 109.48, 97.93, 57.49.

LRMS (ESI) calcd for $C_{11}H_{11}O_2$ $[M+H]^+$: 175.08, found: 175.35.

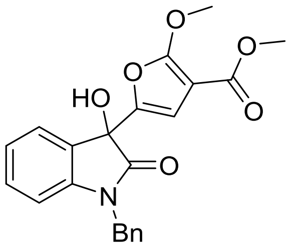

6w-Int

methyl 5-(1-benzyl-3-hydroxy-2-oxoindolin-3-yl)-2-methoxyfuran-3-carboxylate (6w-Int)

The reaction was refluxed at 80°C under nitrogen atmosphere for 48 h.

White solid; 31% yield.

^{1}H NMR (400 MHz, Chloroform-d) δ 7.56 (d, J = 7.4 Hz, 1H), 7.40 – 7.26 (m, 5H), 7.26

– 7.23 (m, 1H), 7.12 (t, *J* = 7.6 Hz, 1H), 6.74 (d, *J* = 7.9 Hz, 1H), 6.54 (s, 1H), 5.00 (d, *J* = 15.8 Hz, 1H), 4.82 (d, *J* = 15.8 Hz, 1H), 4.15 (s, 3H), 3.76 (s, 3H), 3.61 (s, 1H).

^{13}C NMR (126 MHz, Chloroform-*d*) δ 174.47, 163.08, 162.62, 142.54, 140.50, 135.00, 130.47, 128.93, 127.86, 127.29, 127.07, 125.25, 123.53, 111.56, 109.95, 91.89, 72.96, 58.26, 51.34, 43.97.

HRMS (ESI) calcd for $C_{22}H_{19}NO_6Na$ $[M+Na]^+$: 416.1105, found 416.1105.

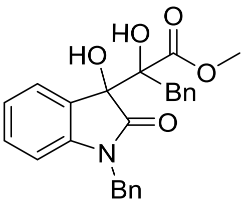

6x-H_2O

methyl 2-(1-benzyl-3-hydroxy-2-oxoindolin-3-yl)-2-hydroxy-3-phenylpropanoate (6x-H_2O)

Pale yellow solid; 99% yield; 1:1 dr.

Data for one of the isomers:

^{1}H NMR (400 MHz, Chloroform-*d*) δ 7.34 – 7.26 (m, 6H), 7.25 – 7.17 (m, 6H), 7.05 (t, *J* = 7.6 Hz, 1H), 6.71 (d, *J* = 7.8 Hz, 1H), 5.11 (d, *J* = 15.6 Hz, 1H), 4.62 (d, *J* = 15.6 Hz, 1H), 4.04 (s, 1H), 3.66 (dd, *J* = 7.7, 5.9 Hz, 2H), 3.52 – 3.45 (m, 4H).

^{13}C NMR (126 MHz, Chloroform-*d*) δ 175.59, 172.44, 143.70, 135.27, 135.15, 130.50, 130.22, 128.84, 128.14, 127.77, 127.49, 127.39, 126.96, 124.58, 123.04, 109.47, 81.60, 78.15, 52.56, 44.00, 37.24.

HRMS (ESI) calcd for $C_{25}H_{23}NO_5Na$ $[M+Na]^+$: 440.1468, found 440.1466.

11. NMR Spectra

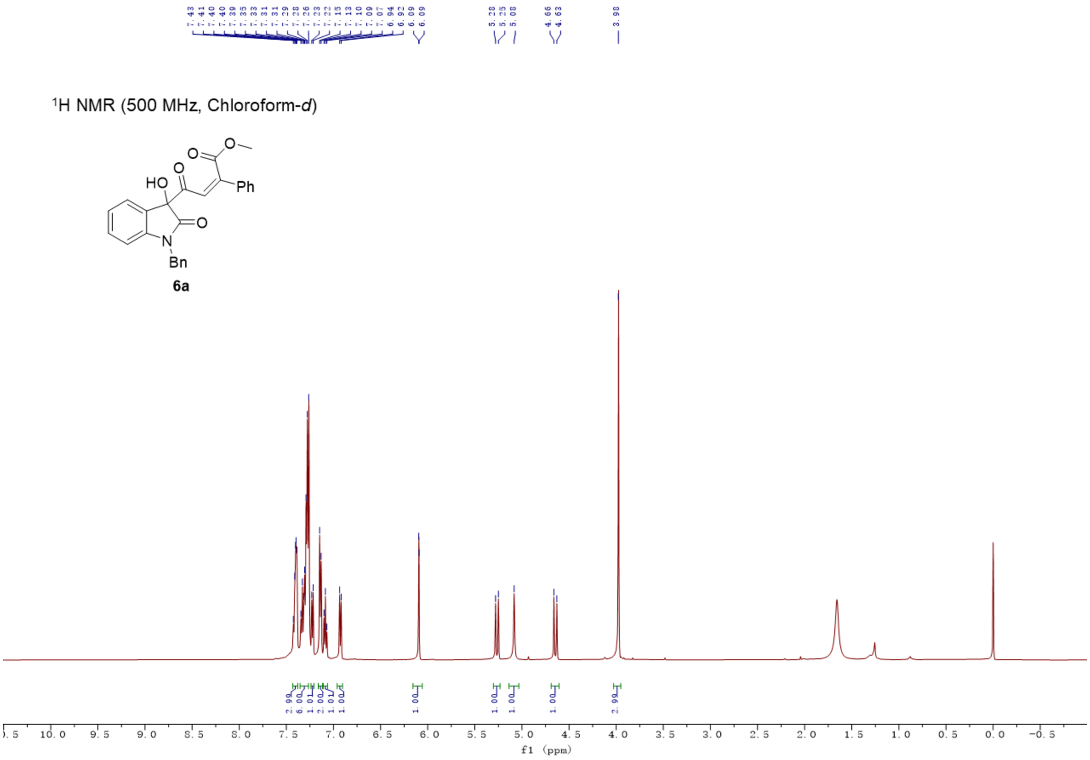
1H NMR (500 MHz, Chloroform-d)
HO
O
Ph
N
Bn
6a
f1 (ppm)

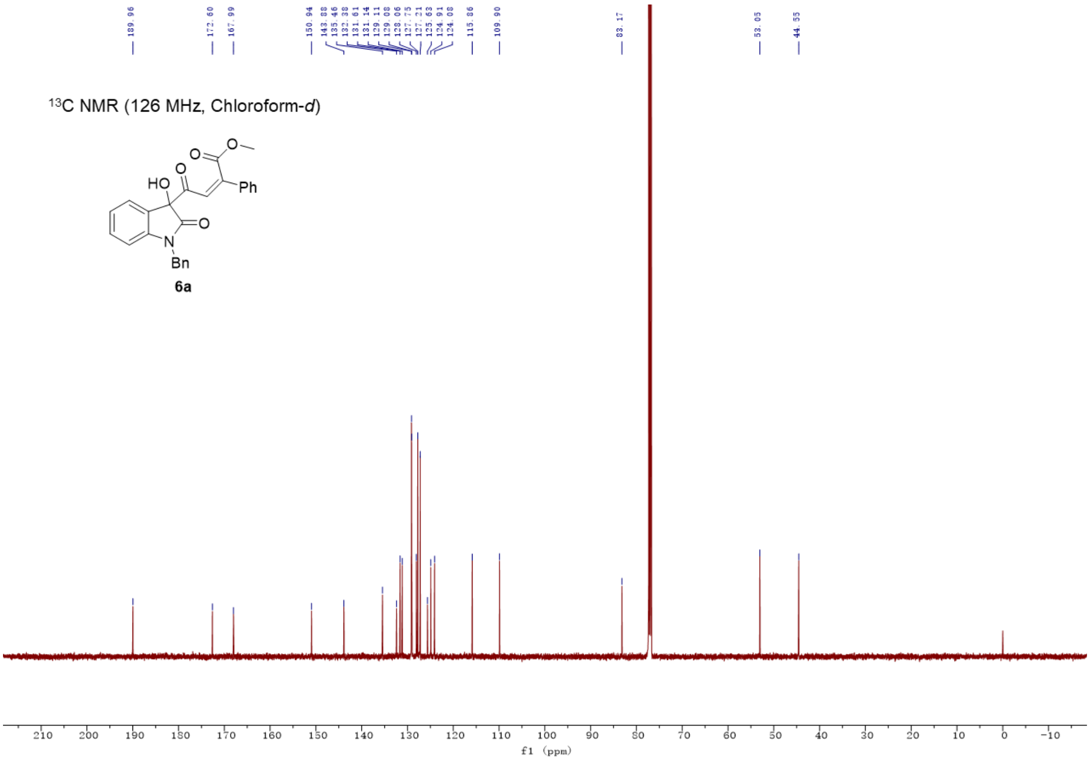
13C NMR (126 MHz, Chloroform-d)
HO
O
Ph
N
Bn
6a
f1 (ppm)

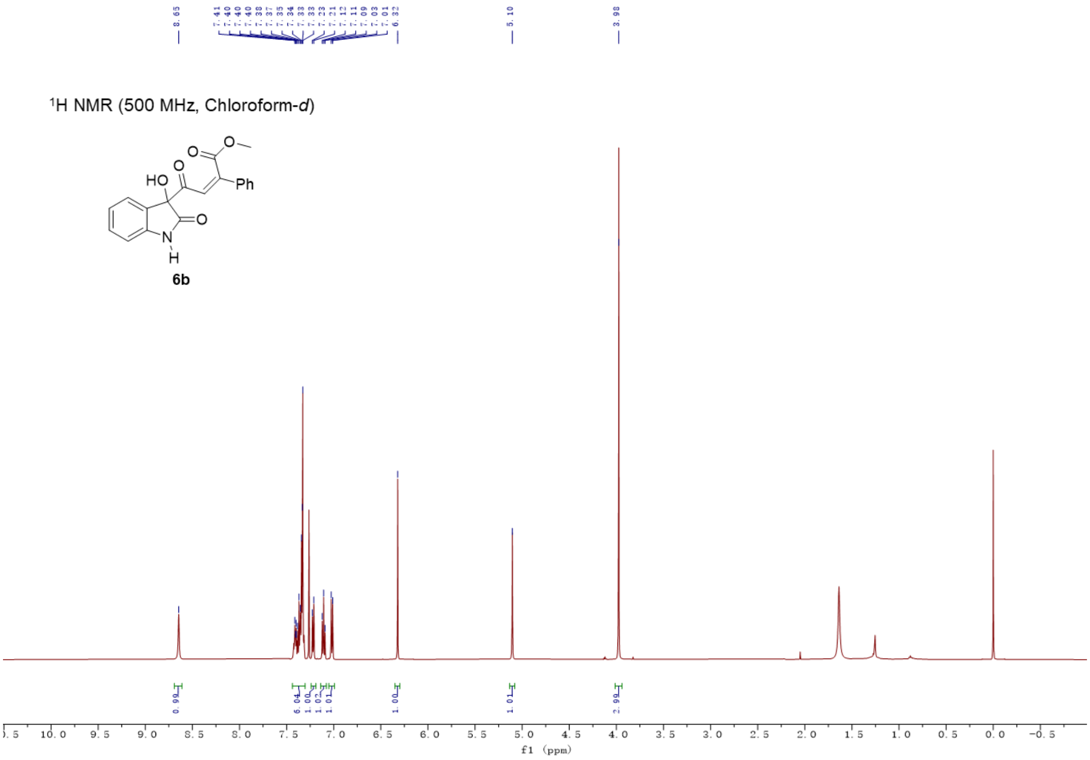

1H NMR (500 MHz, Chloroform-d)
HO
O
O
O
Ph
N
H
6b
f1 (ppm)

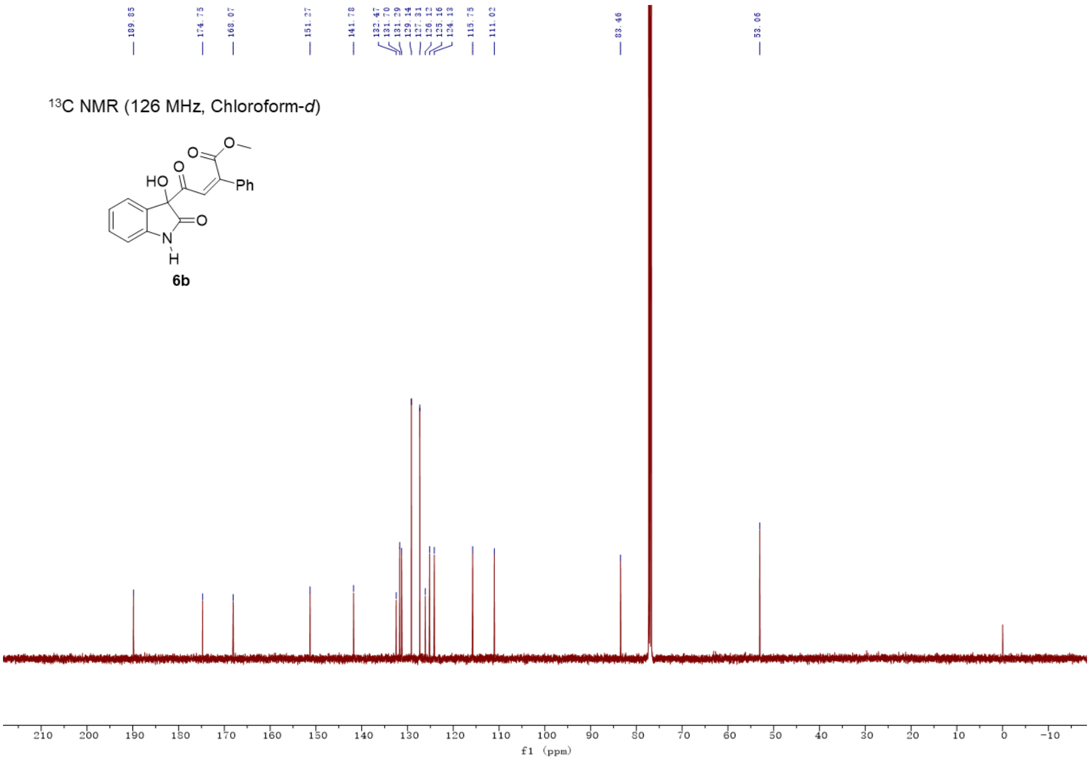

13C NMR (126 MHz, Chloroform-d)
HO
O
O
O
Ph
N
H
6b
f1 (ppm)

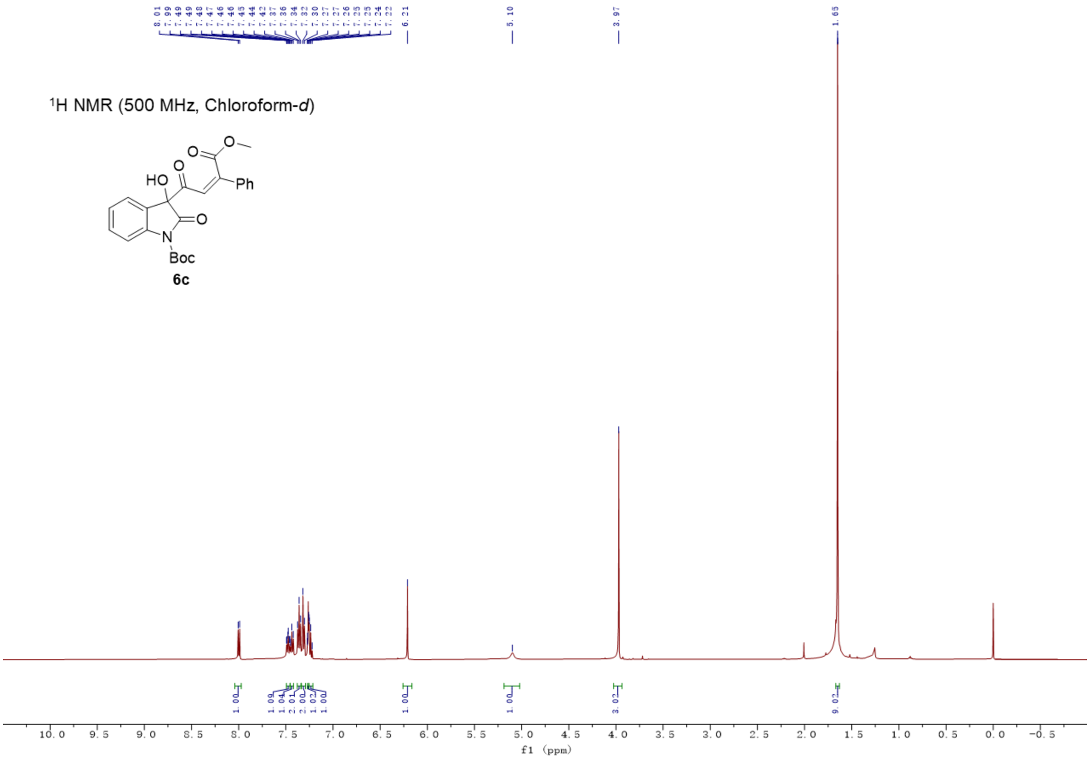
6.21
5.10
3.97
1.65
^{1}H NMR (500 MHz, Chloroform-*d*)
HO
Ph
N
Boc
6c
f1 (ppm)

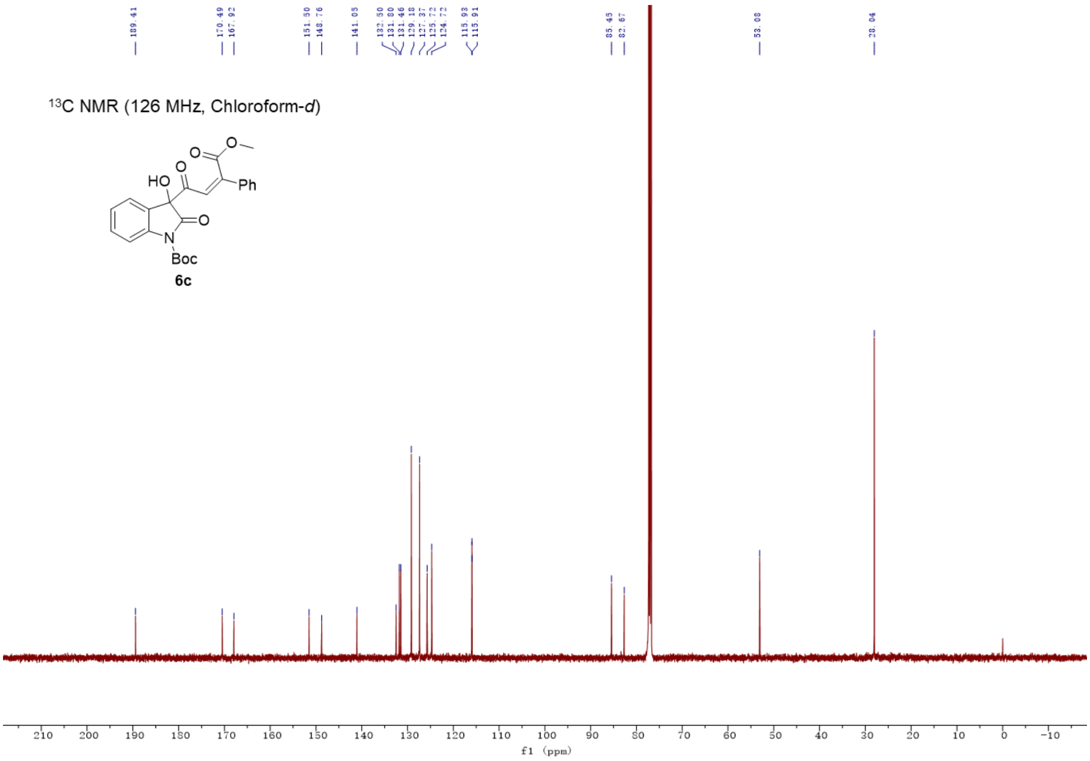
189.41
170.49
167.92
151.50
148.76
141.05
85.45
82.67
53.08
28.04
^{13}C NMR (126 MHz, Chloroform-*d*)
HO
Ph
N
Boc
6c
f1 (ppm)

^{1}H NMR (400 MHz, Chloroform-*d*)

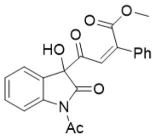

6d

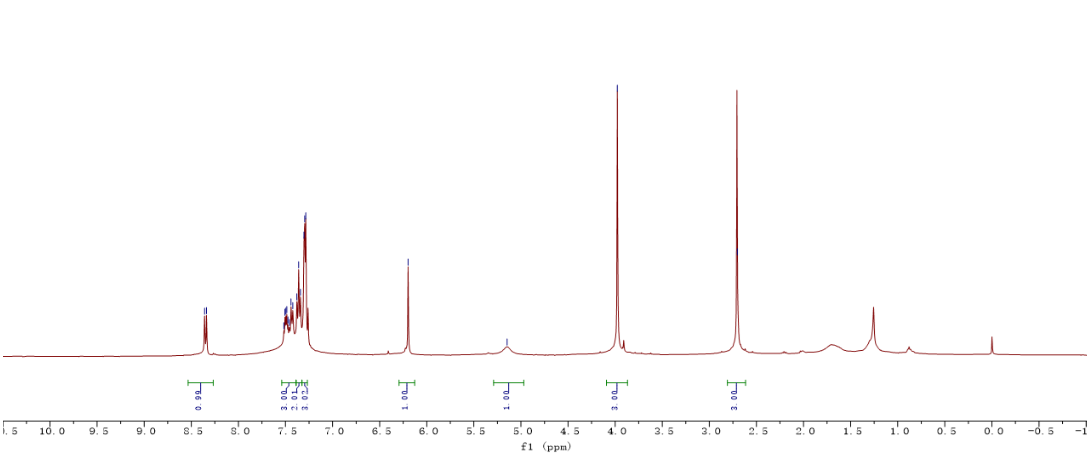

^{13}C NMR (126 MHz, Chloroform-*d*)

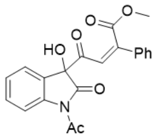

6d

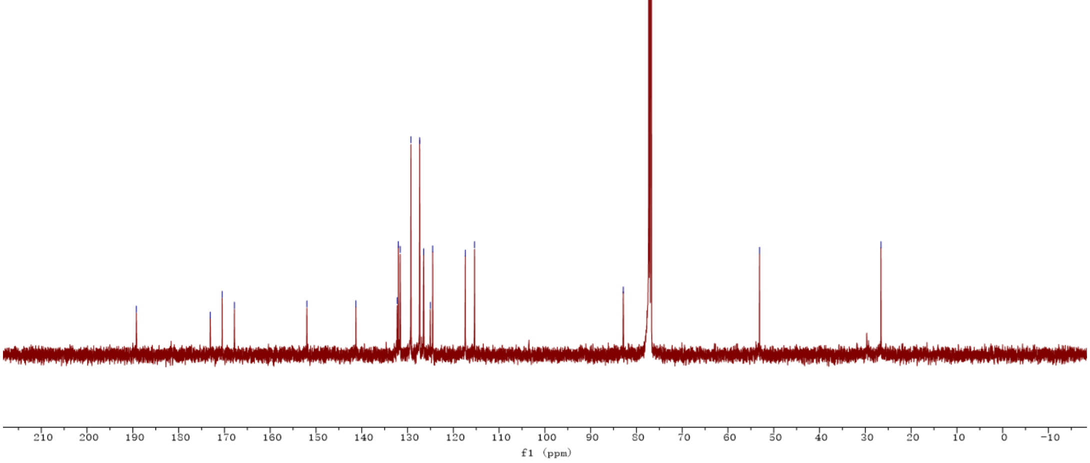

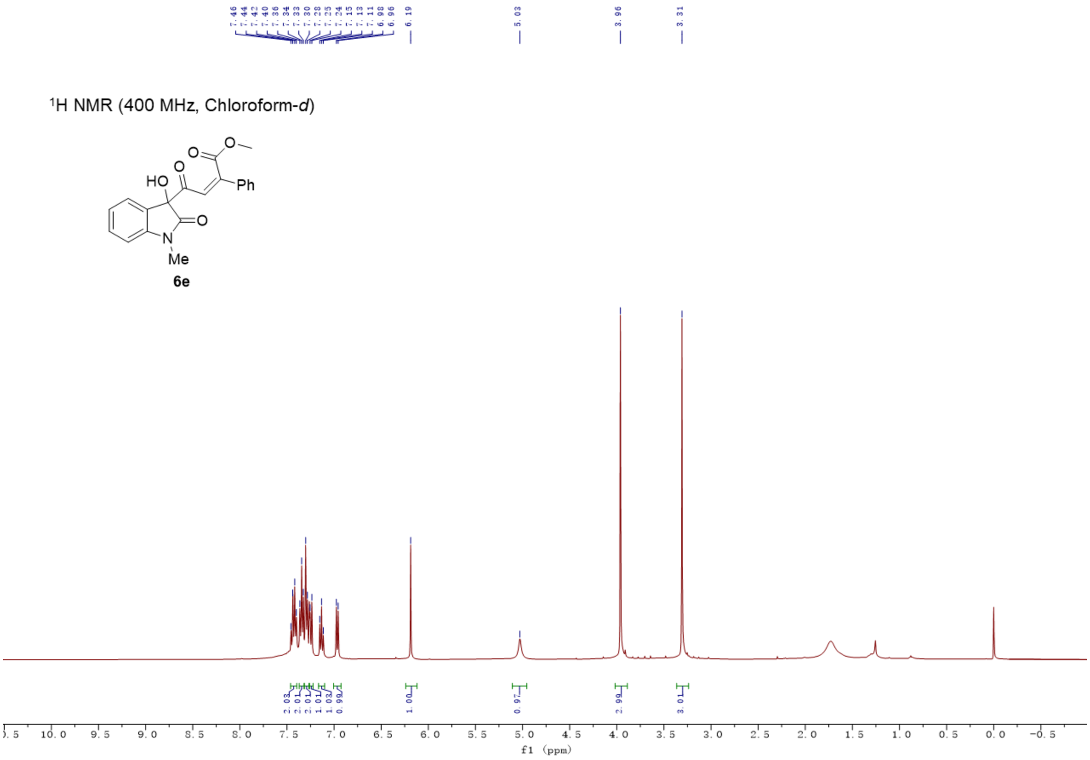

7.46
7.44
7.42
7.40
7.36
7.34
7.33
7.30
7.28
7.25
7.24
7.15
7.13
7.11
6.98
6.96
6.19
5.03
3.96
3.31
1H NMR (400 MHz, Chloroform-d)
6e
2.03
2.01
2.01
1.01
1.03
0.98
1.00
0.97
2.99
3.01
f1 (ppm)

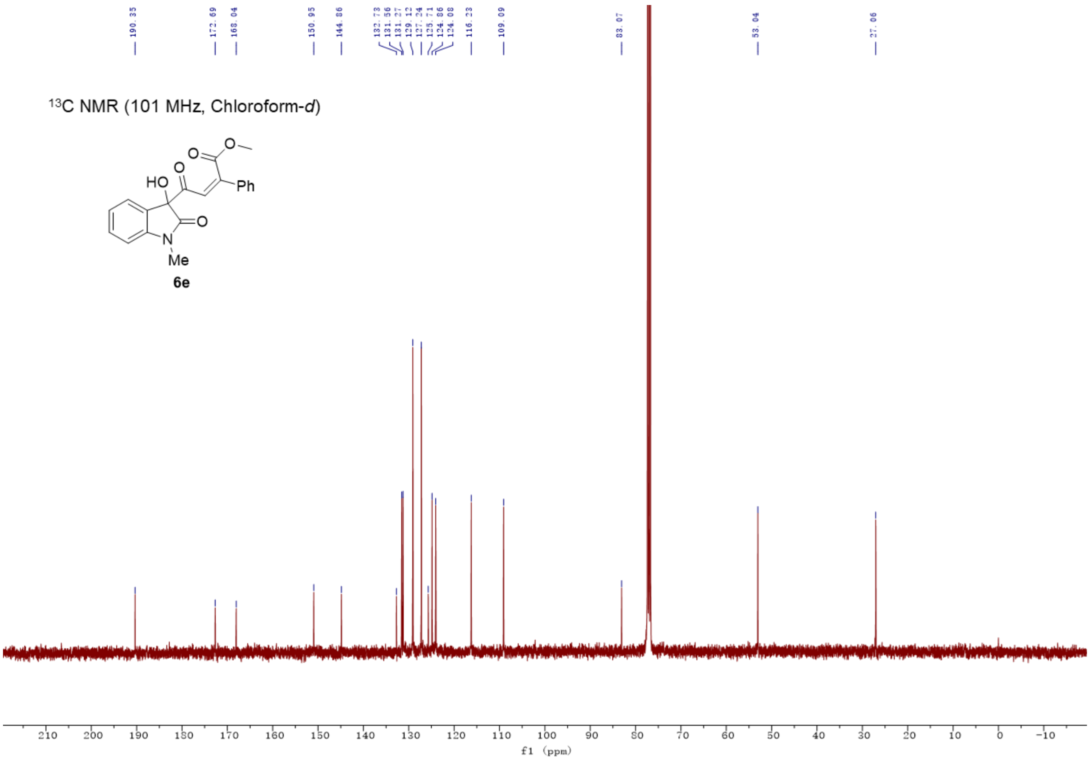

190.35
172.69
168.04
150.95
144.86
132.73
131.56
131.27
129.12
127.24
125.71
124.86
124.08
116.23
109.09
83.07
53.04
27.06
13C NMR (101 MHz, Chloroform-d)
6e
f1 (ppm)

^{1}H NMR (500 MHz, Chloroform-*d*)

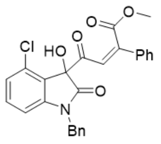

6f

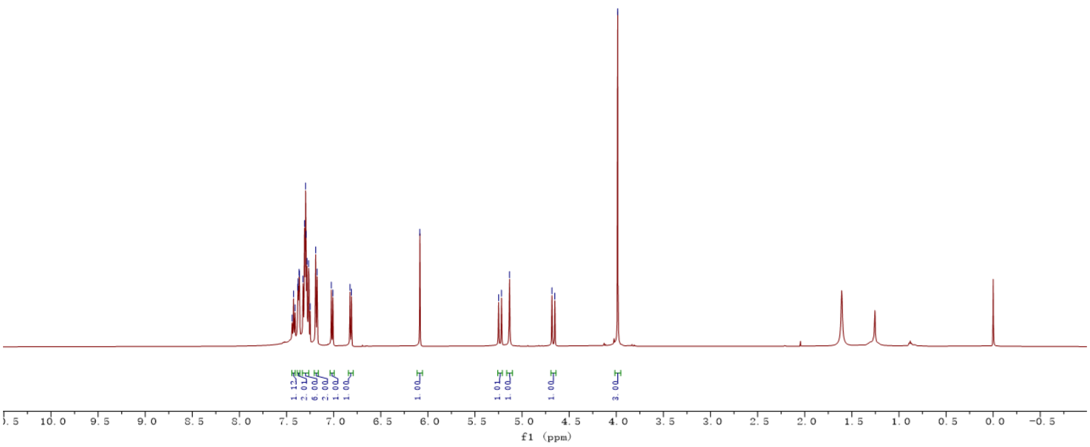

^{13}C NMR (126 MHz, Chloroform-*d*)

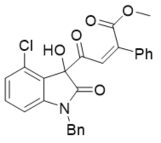

6f

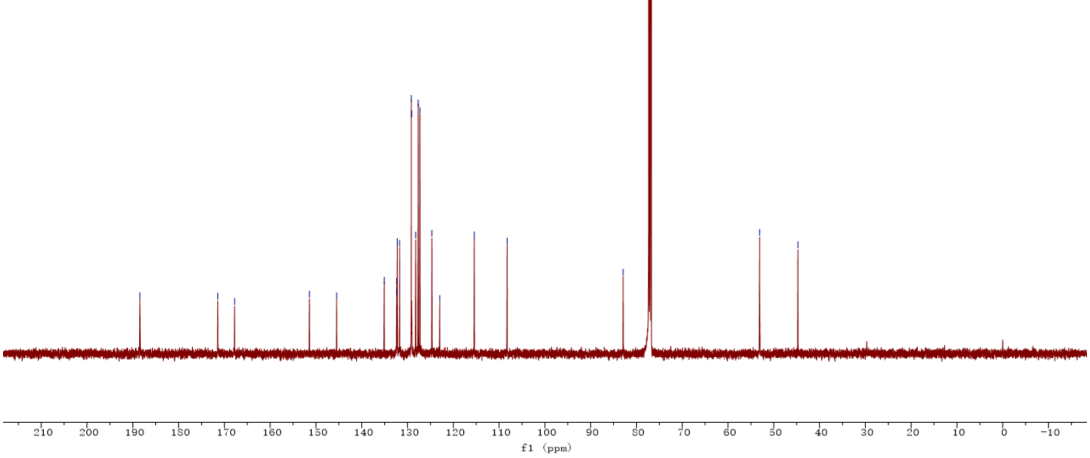

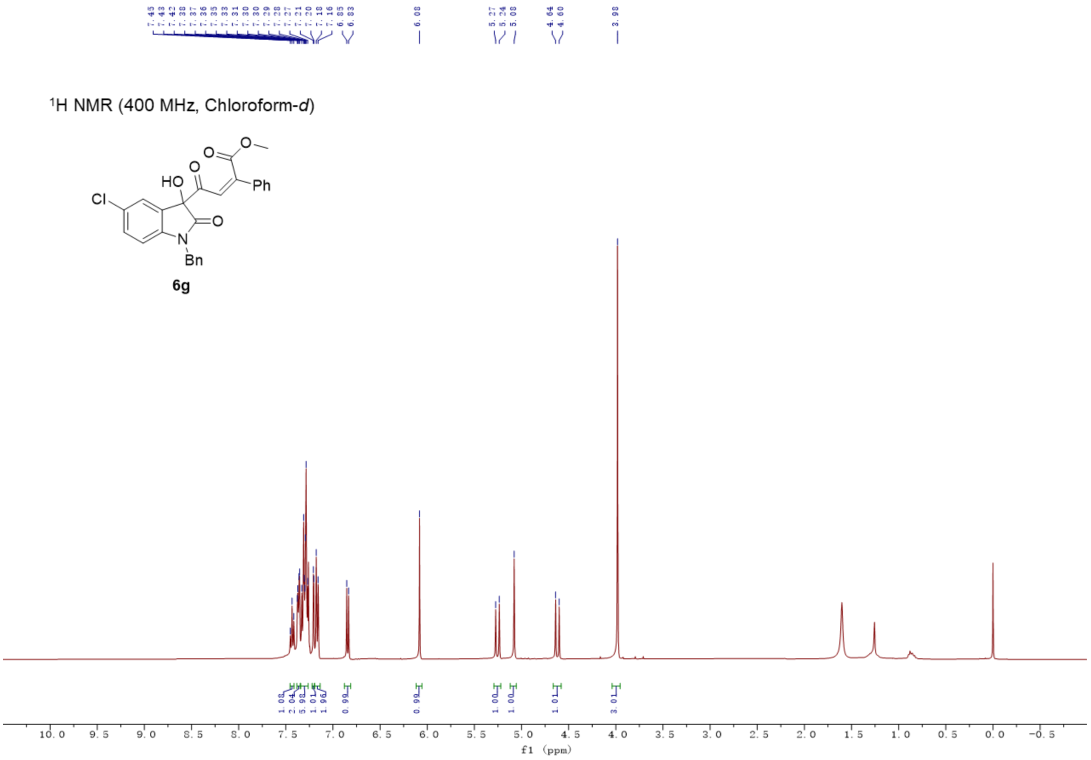

1H NMR (400 MHz, Chloroform-d)
O
O
O
HO
Ph
Cl
O
N
Bn
6g
f1 (ppm)

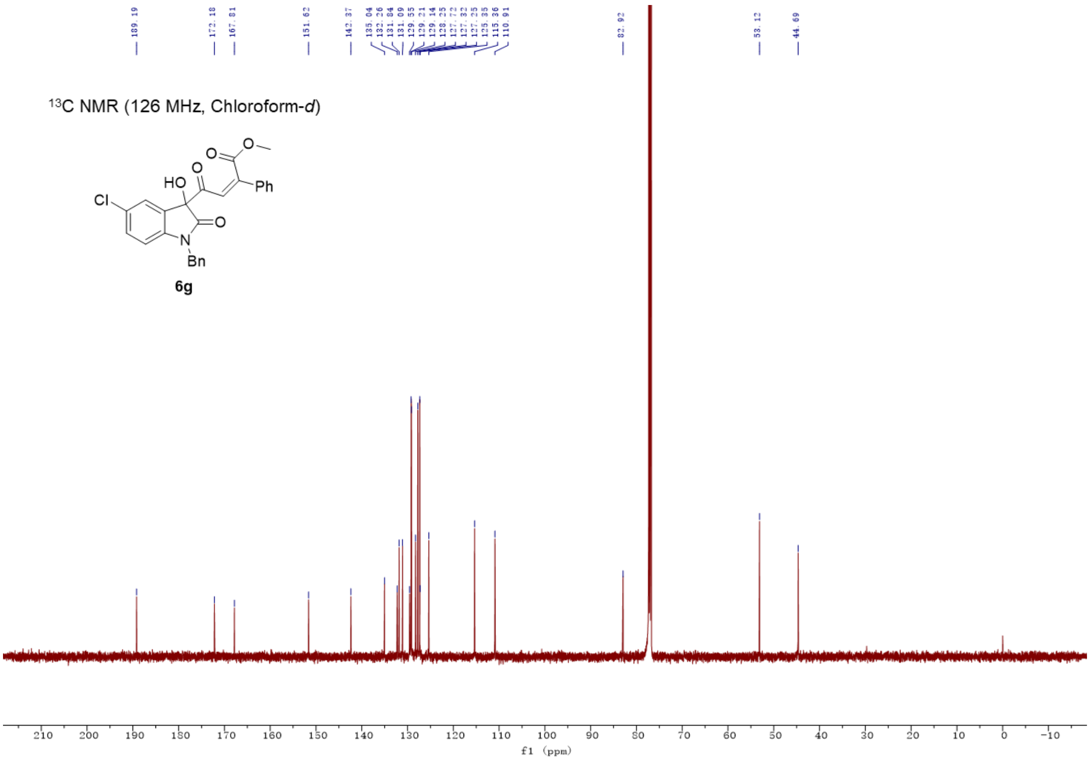

13C NMR (126 MHz, Chloroform-d)
O
O
O
HO
Ph
Cl
O
N
Bn
6g
f1 (ppm)

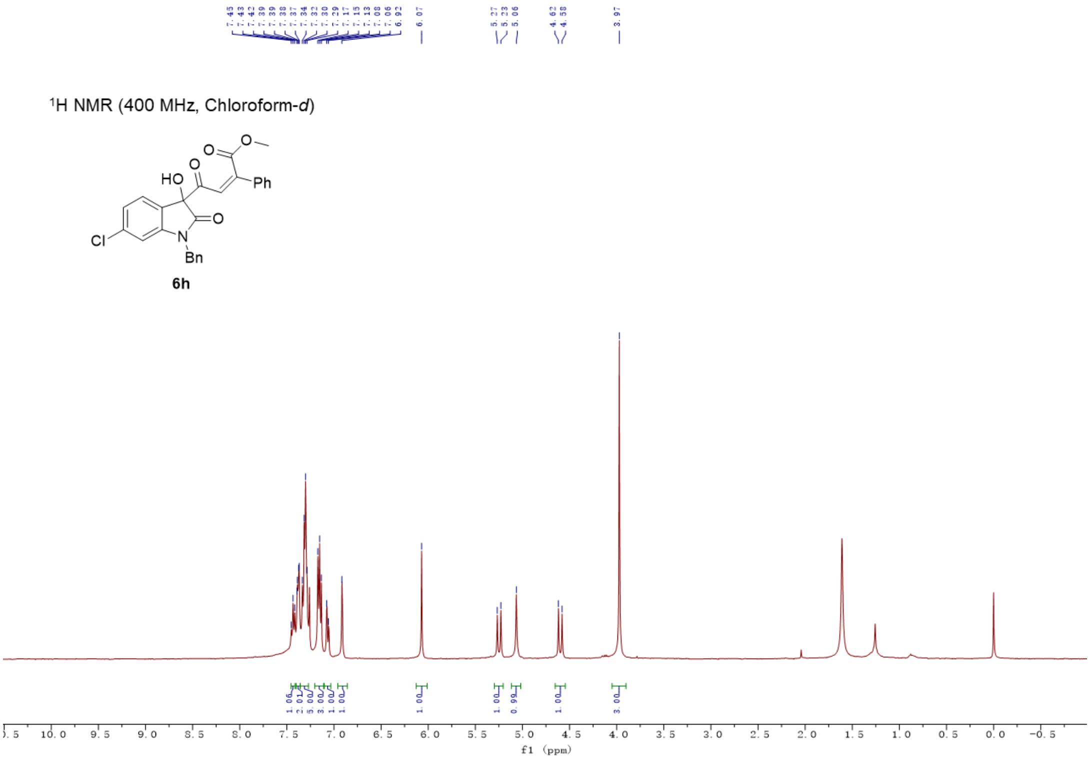
1H NMR (400 MHz, Chloroform-d)
O
O
HO
Ph
O
Cl
N
Bn
6h
f1 (ppm)

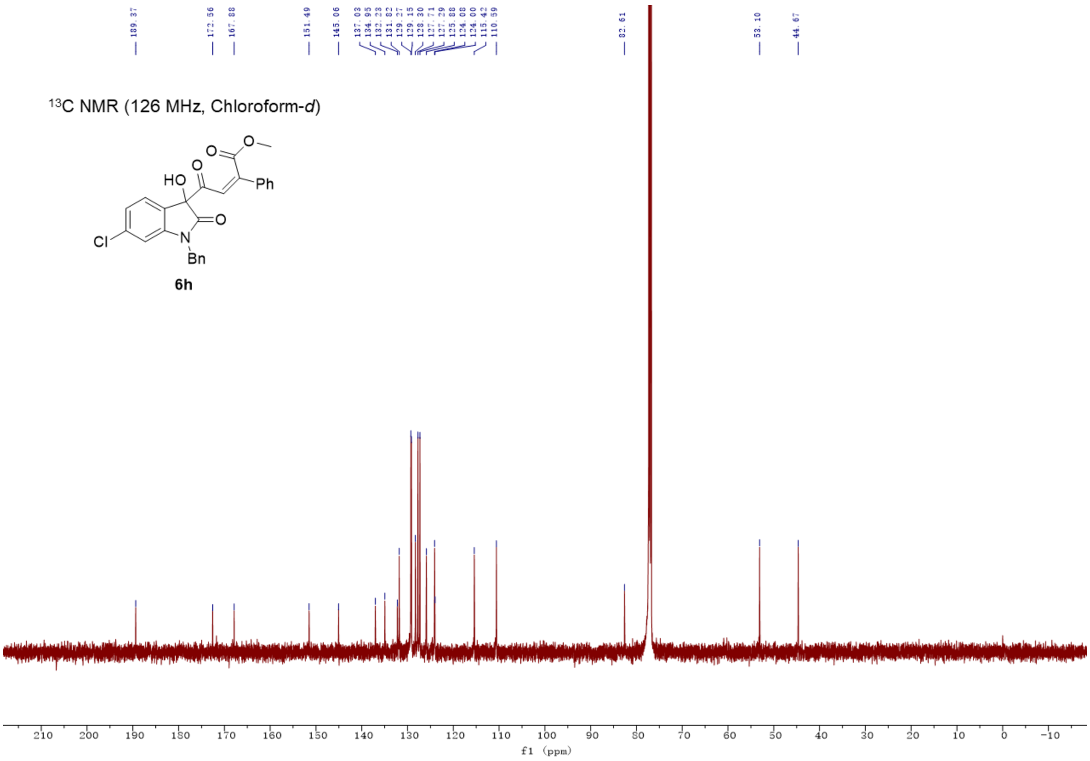
13C NMR (126 MHz, Chloroform-d)
O
O
HO
Ph
O
Cl
N
Bn
6h
f1 (ppm)

^{1}H NMR (400 MHz, Chloroform-d)

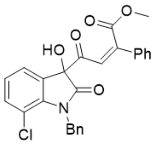

6i

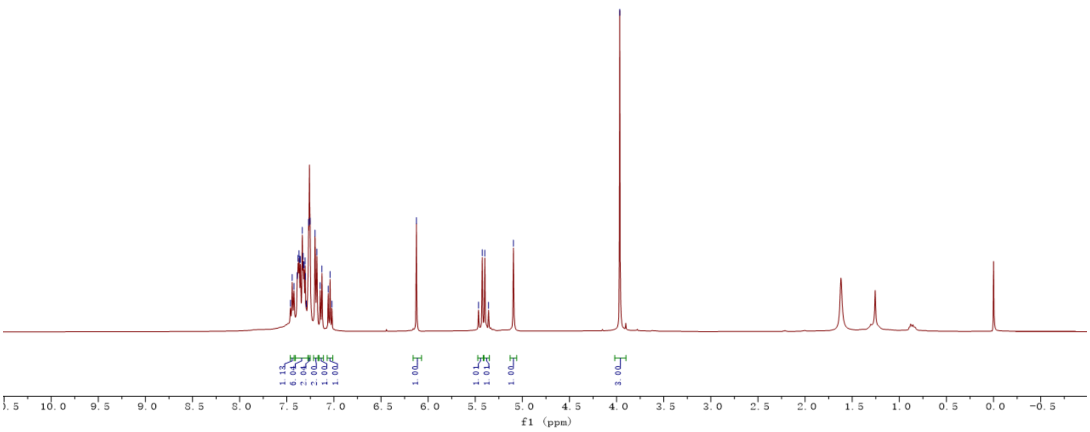

^{13}C NMR (126 MHz, Chloroform-d)

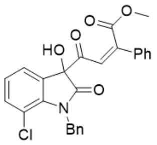

6i

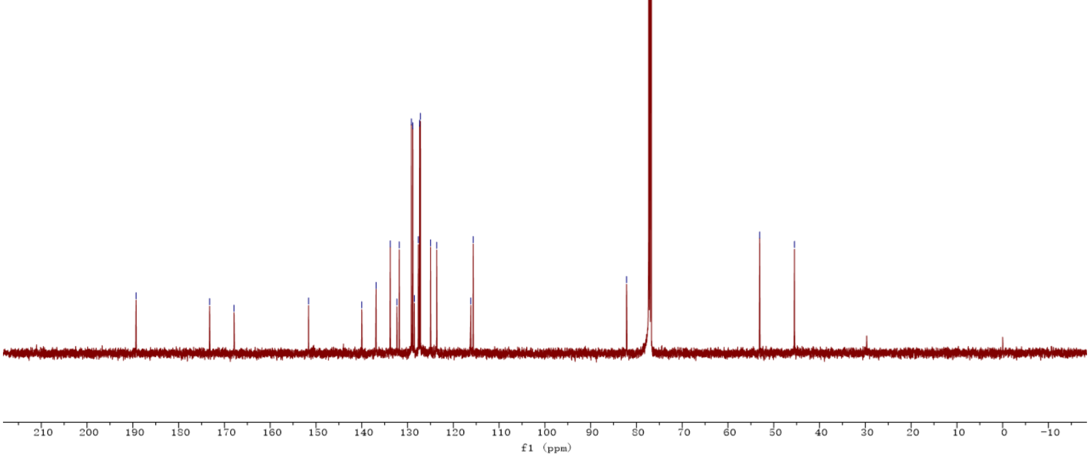

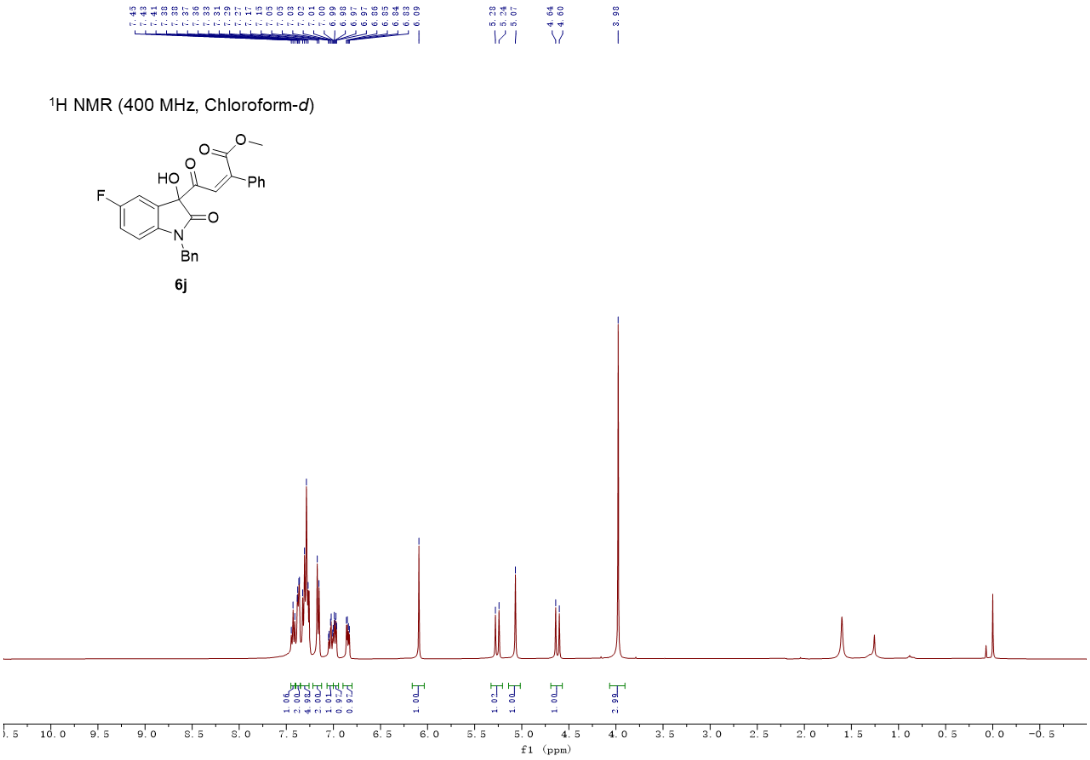
1H NMR (400 MHz, Chloroform-d)
O
HO
Ph
F
N
Bn
6j
f1 (ppm)

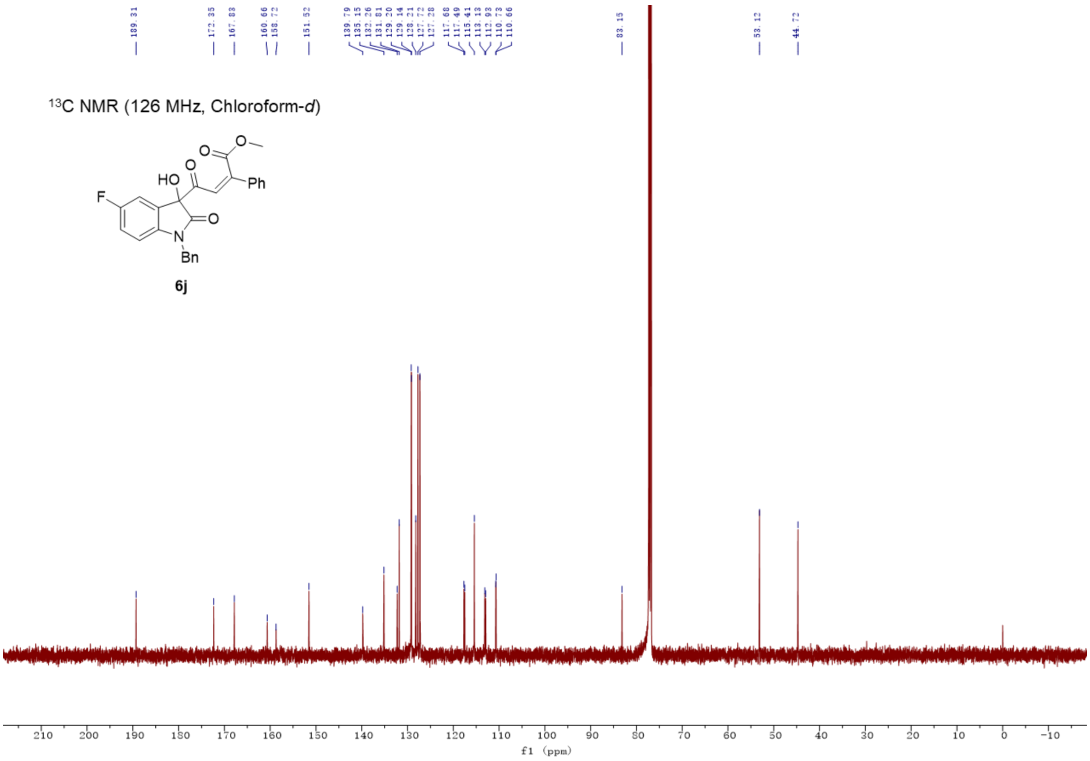
13C NMR (126 MHz, Chloroform-d)
O
HO
Ph
F
N
Bn
6j
f1 (ppm)

^19F NMR (376 MHz, Chloroform-*d*)

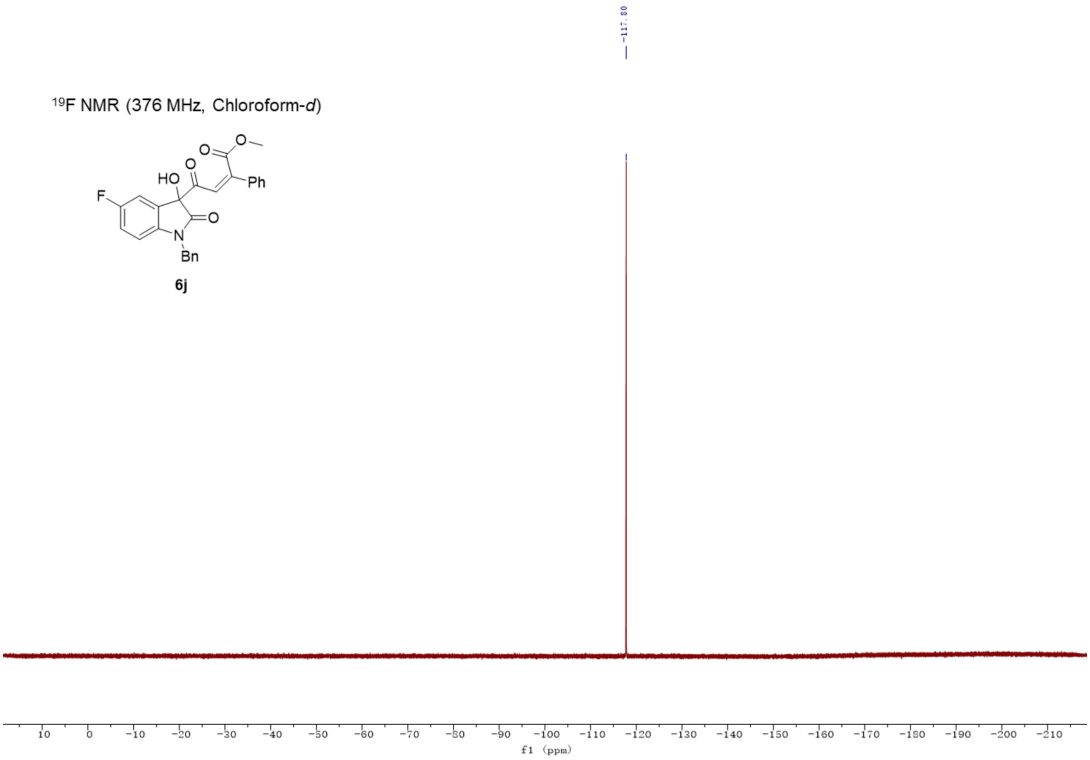

6j

^1H NMR (400 MHz, Chloroform-*d*)

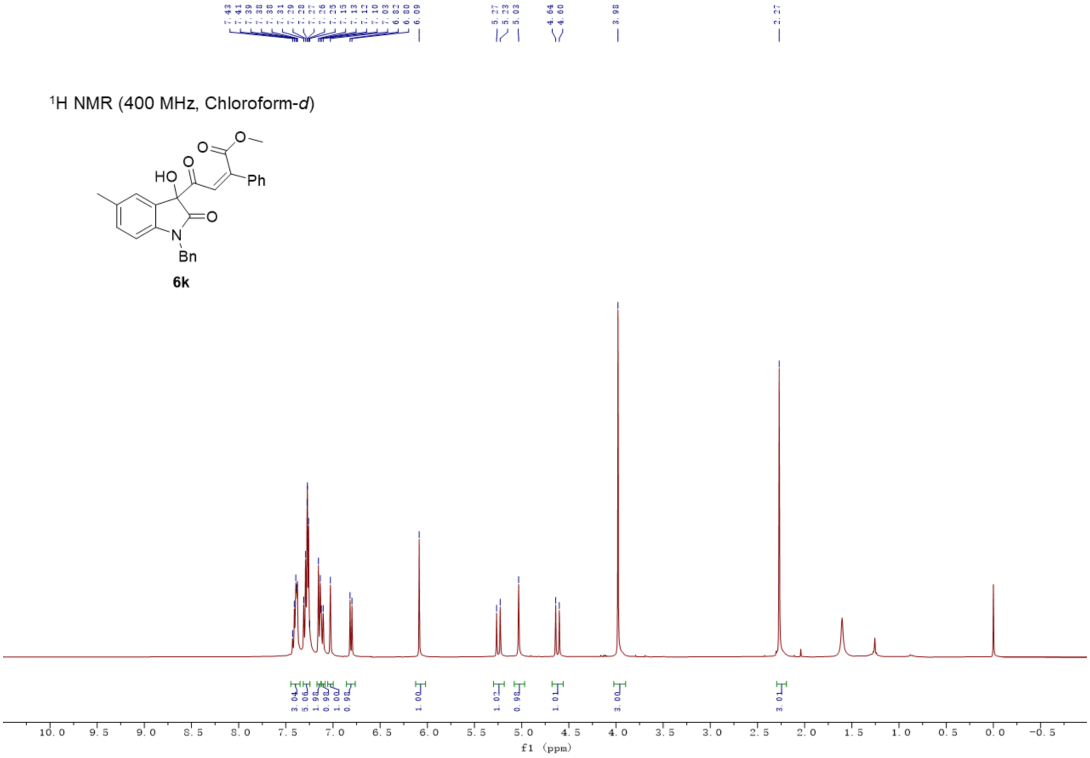

6k

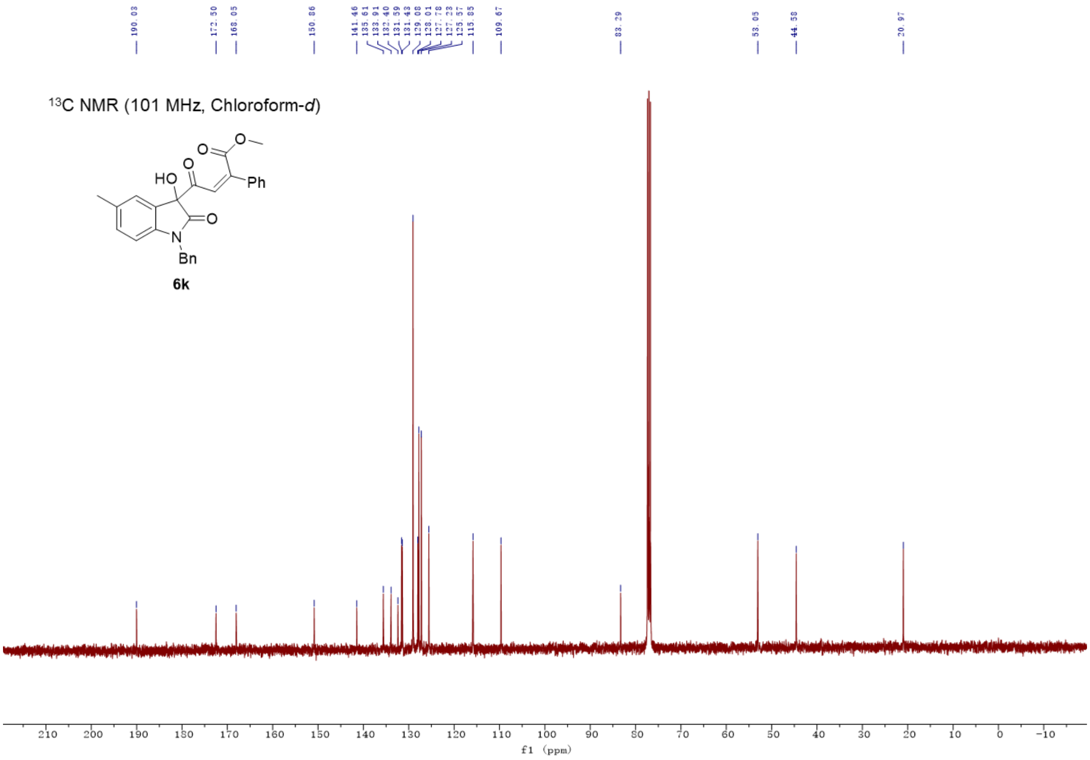
13C NMR (101 MHz, Chloroform-d)
HO
O
O
O
Ph
N
Bn
O
6k

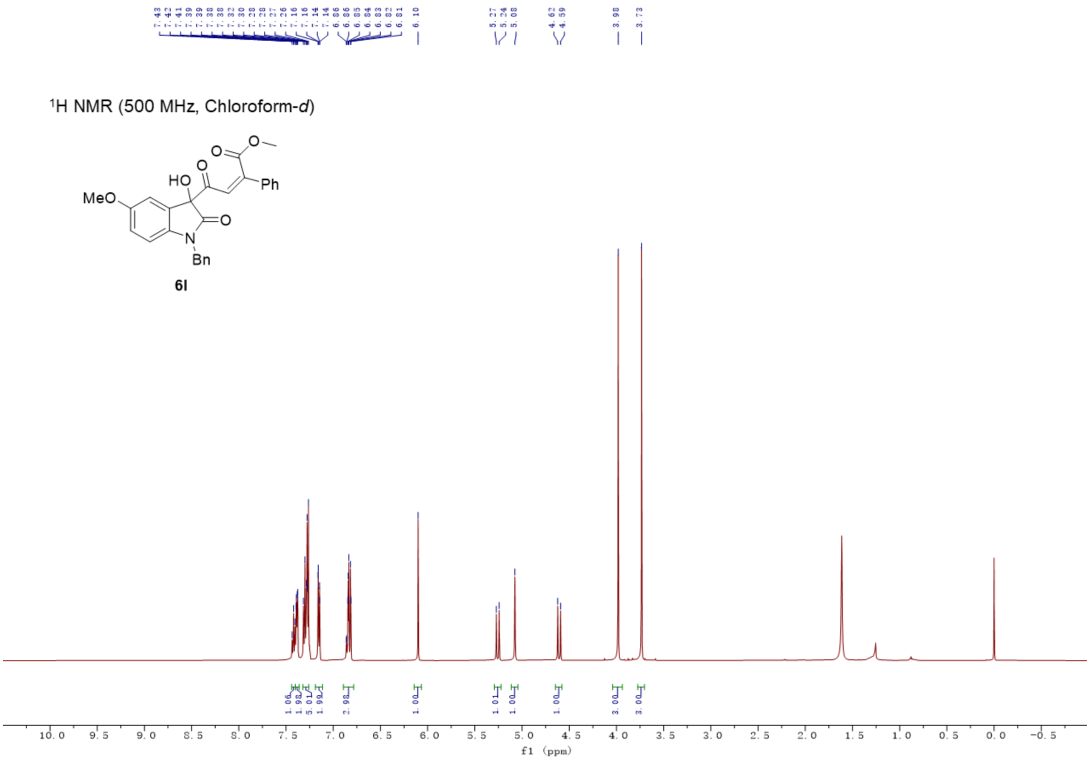
1H NMR (500 MHz, Chloroform-d)
MeO
HO
O
O
O
Ph
N
Bn
O
6l

^{13}C NMR (126 MHz, Chloroform-*d*)

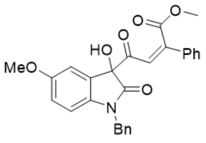

6l

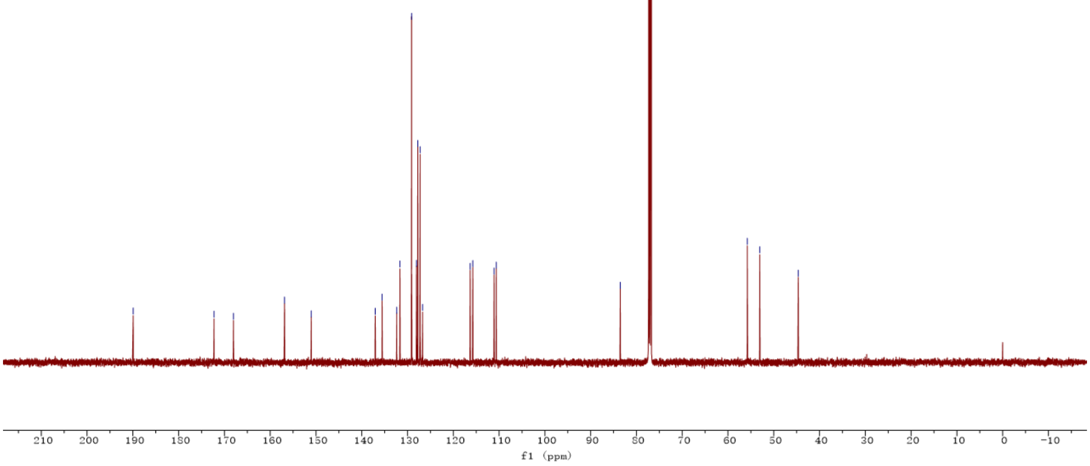

^{1}H NMR (500 MHz, Chloroform-*d*)

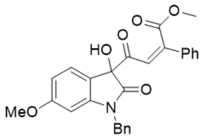

6m

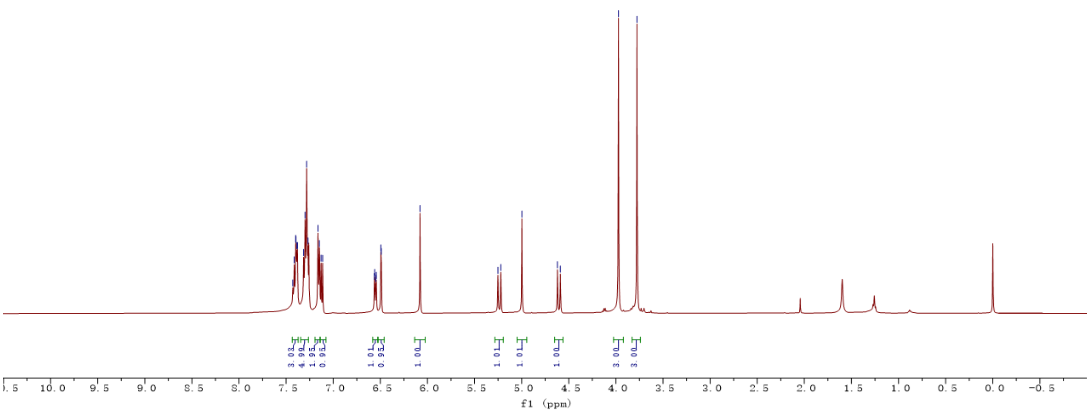

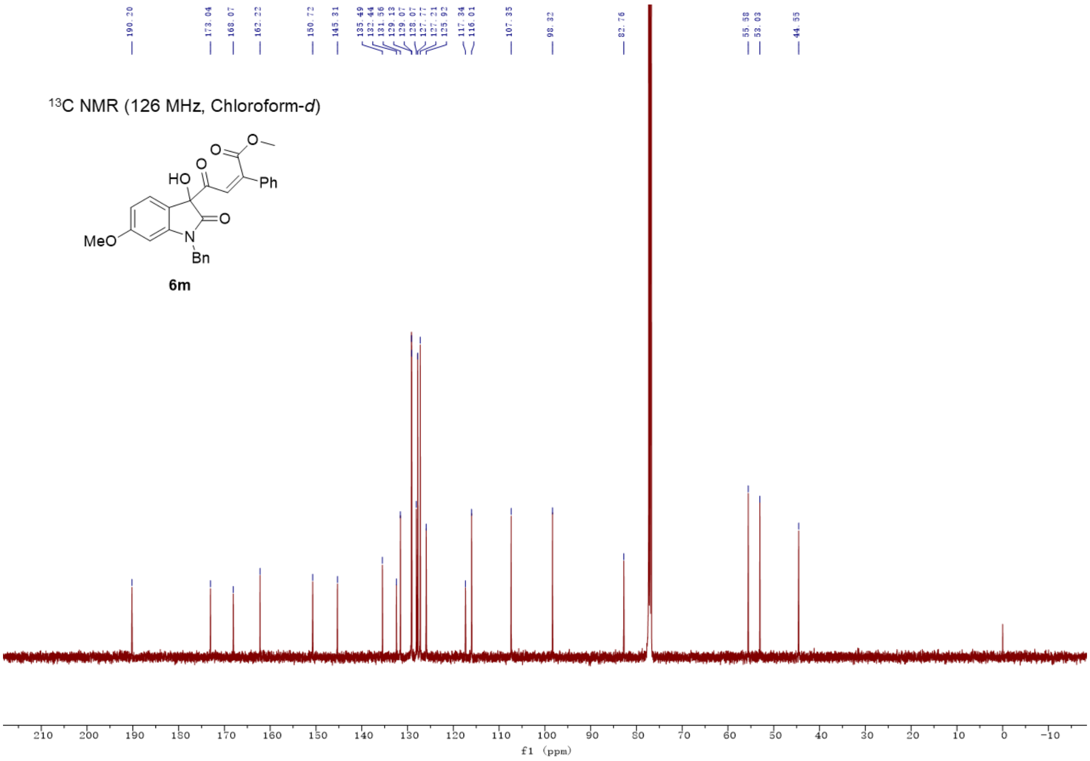

190.20
173.04
168.07
162.22
150.72
145.31
135.49
132.44
131.56
129.13
129.07
128.07
127.71
127.21
125.92
117.34
116.01
107.35
98.32
82.76
55.58
53.03
44.55
^{13}C NMR (126 MHz, Chloroform-*d*)
HO
MeO
N
Bn
Ph
6m
f1 (ppm)

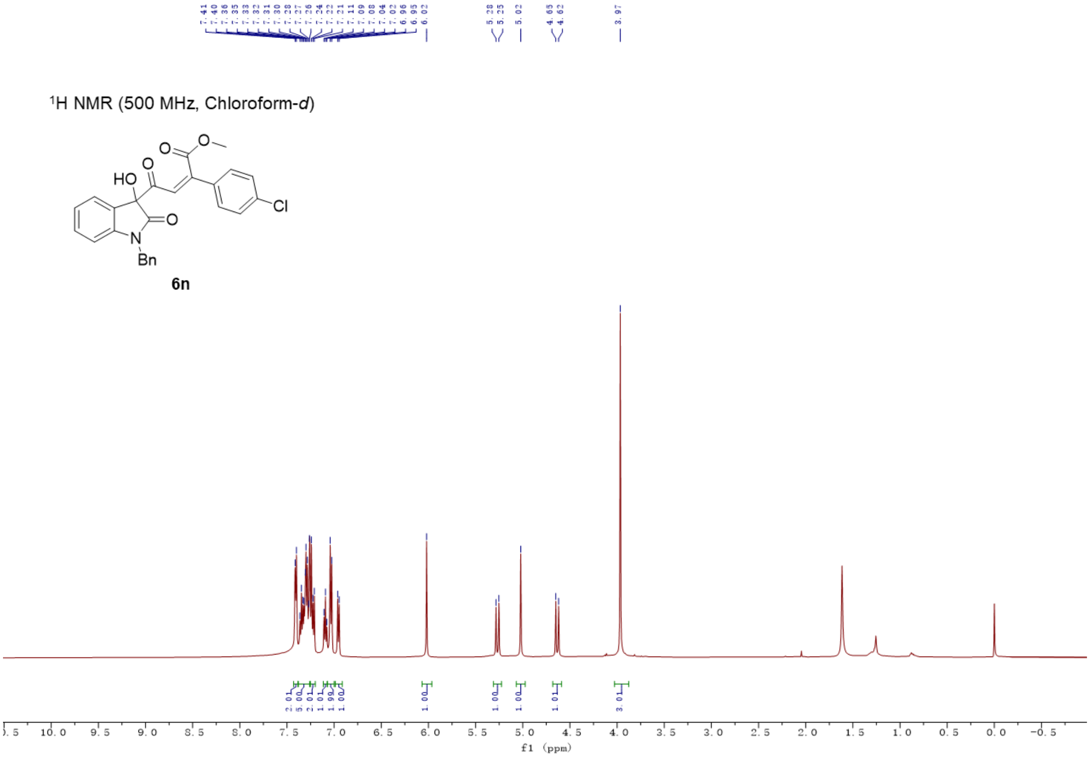

6.02
5.28
5.25
5.02
4.65
4.62
3.97
^{1}H NMR (500 MHz, Chloroform-*d*)
HO
N
Bn
Cl
6n
f1 (ppm)

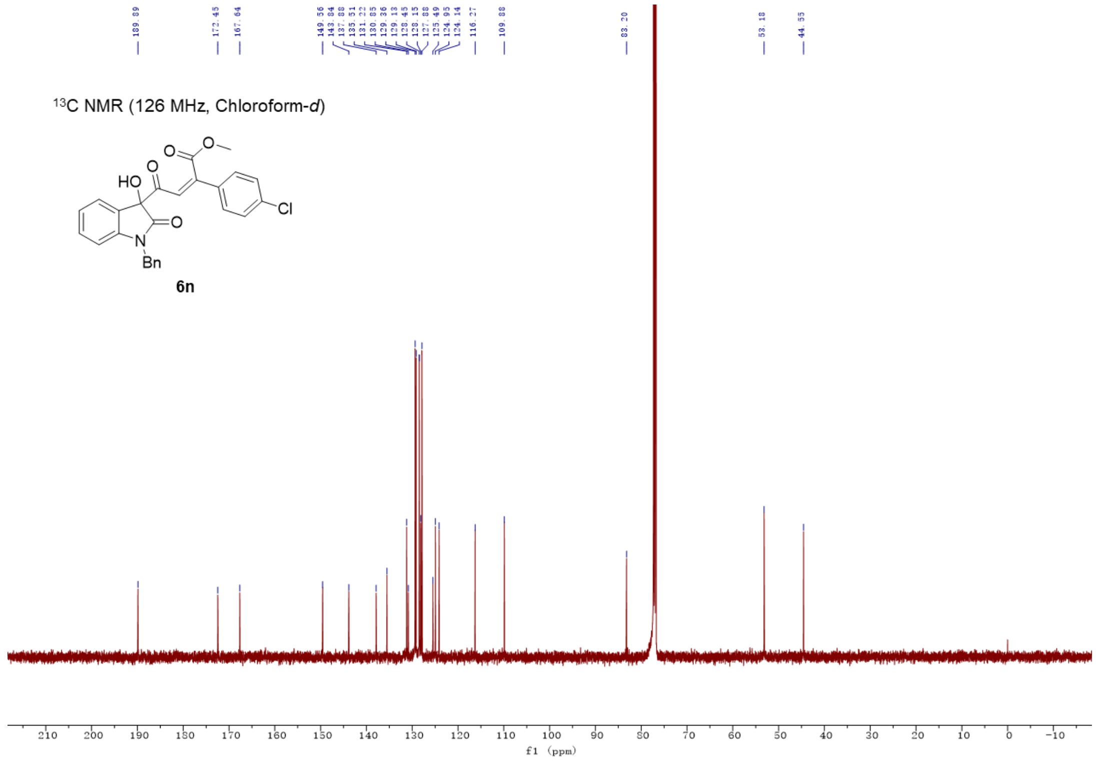
189.89
172.45
167.64
149.56
143.84
137.88
135.51
131.22
130.85
129.36
129.18
128.45
128.15
127.88
125.49
124.95
124.14
116.27
109.88
83.20
53.18
44.55
^{13}C NMR (126 MHz, Chloroform-*d*)
HO
N
Bn
Cl
6n
f1 (ppm)

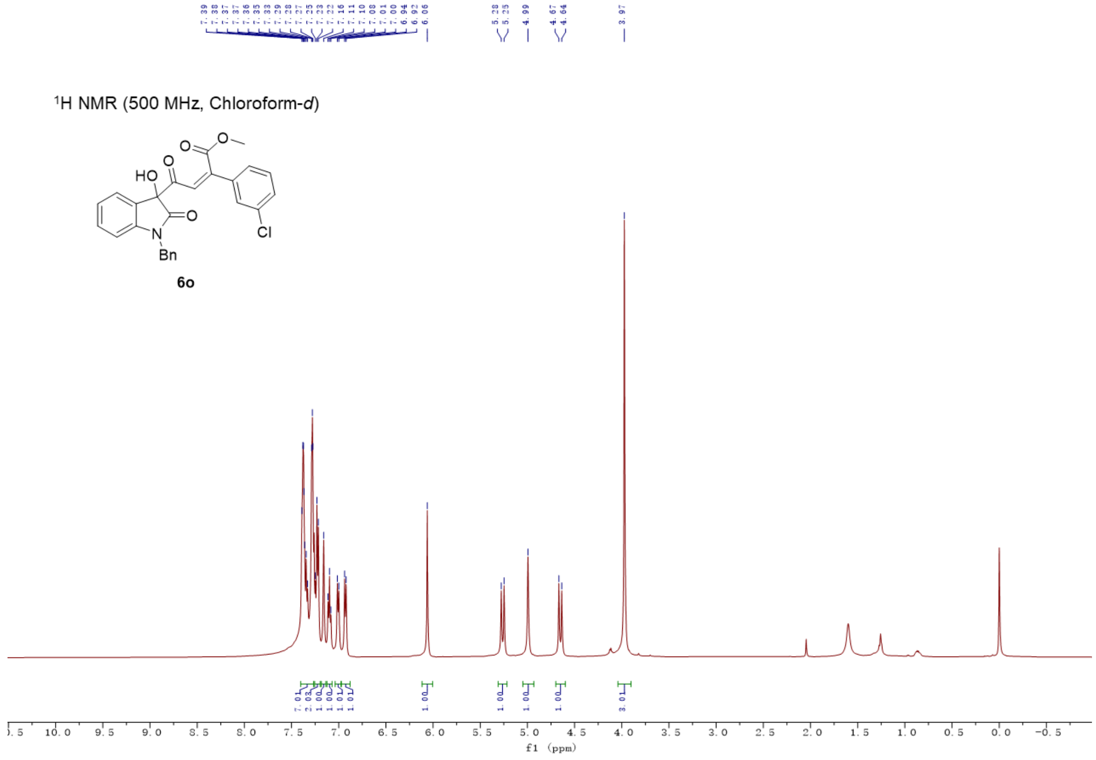
6.08
5.28
5.25
4.99
4.67
4.64
3.97
^{1}H NMR (500 MHz, Chloroform-*d*)
HO
N
Bn
Cl
6o
f1 (ppm)

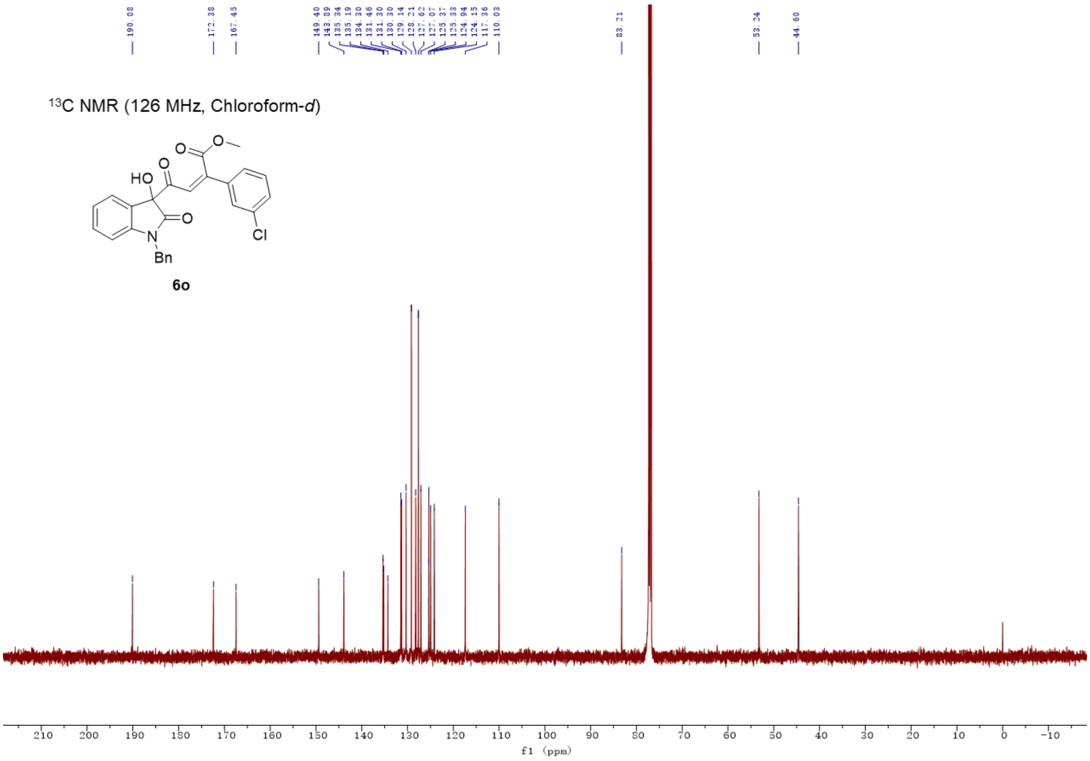
13C NMR (126 MHz, Chloroform-d)
6o

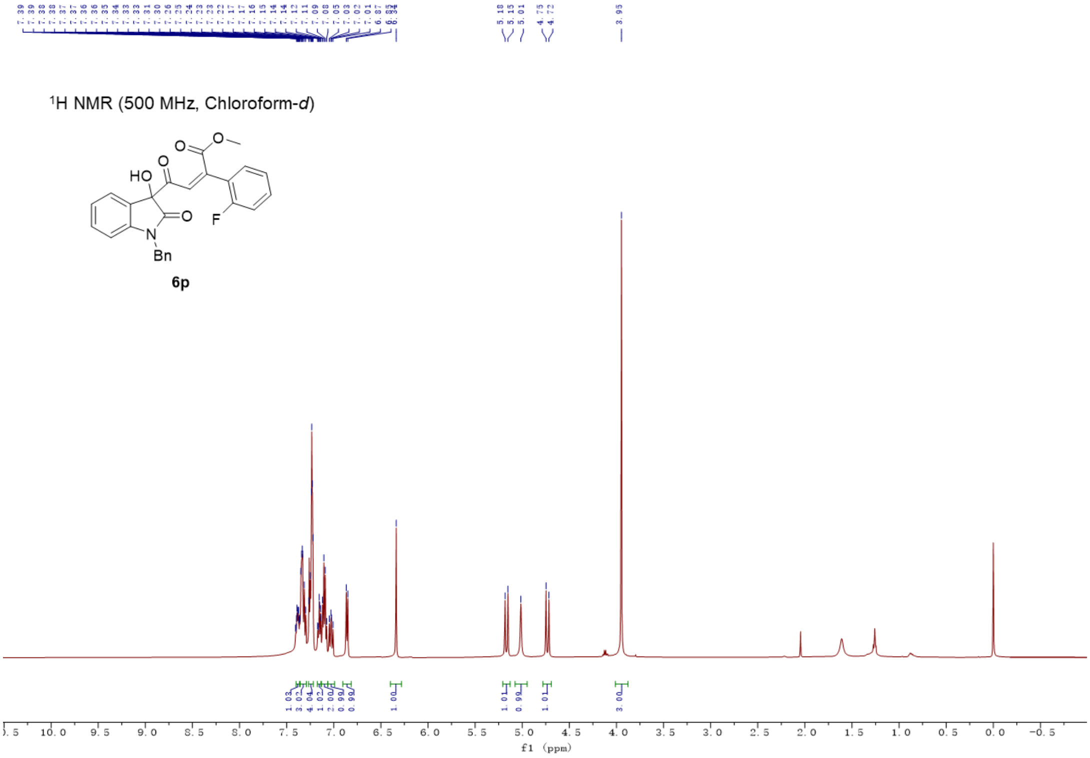
1H NMR (500 MHz, Chloroform-d)
6p

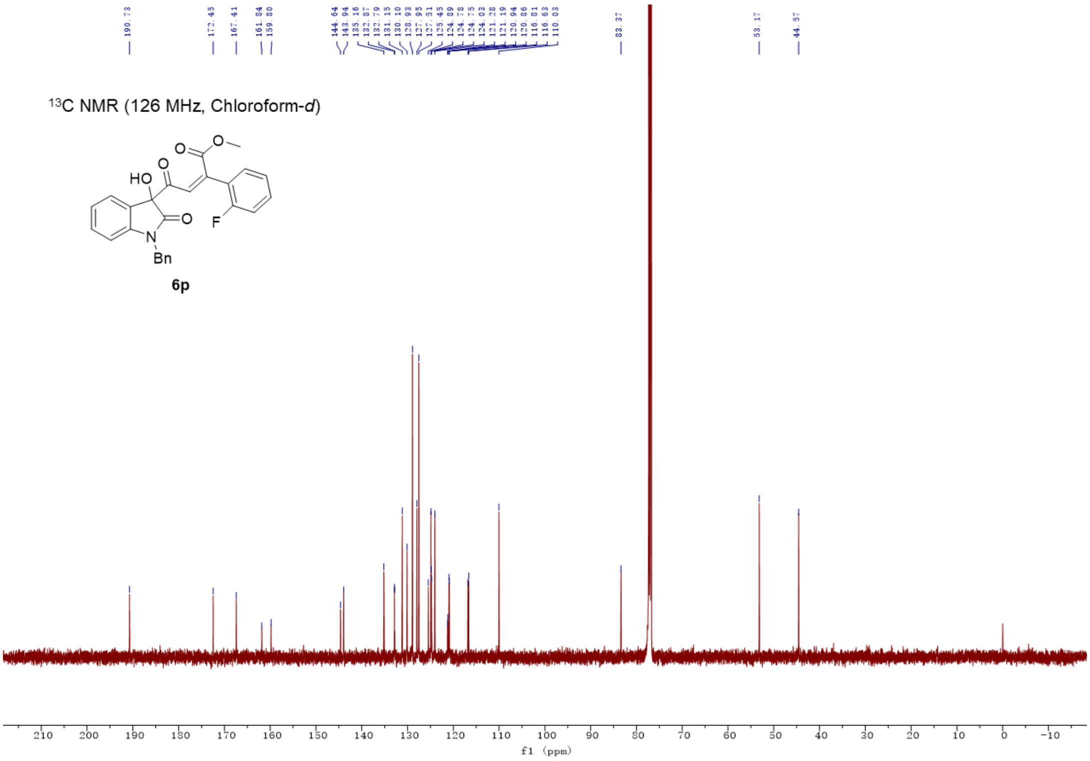
190.73
172.45
167.41
161.84
159.80
144.64
143.94
135.16
132.87
132.79
131.15
130.10
128.93
127.95
127.51
125.45
124.89
124.78
124.75
124.03
121.28
121.19
120.94
120.86
116.81
116.63
110.03
83.37
53.17
44.57
13C NMR (126 MHz, Chloroform-d)
HO
O
O
O
N
Bn
F
6p
f1 (ppm)

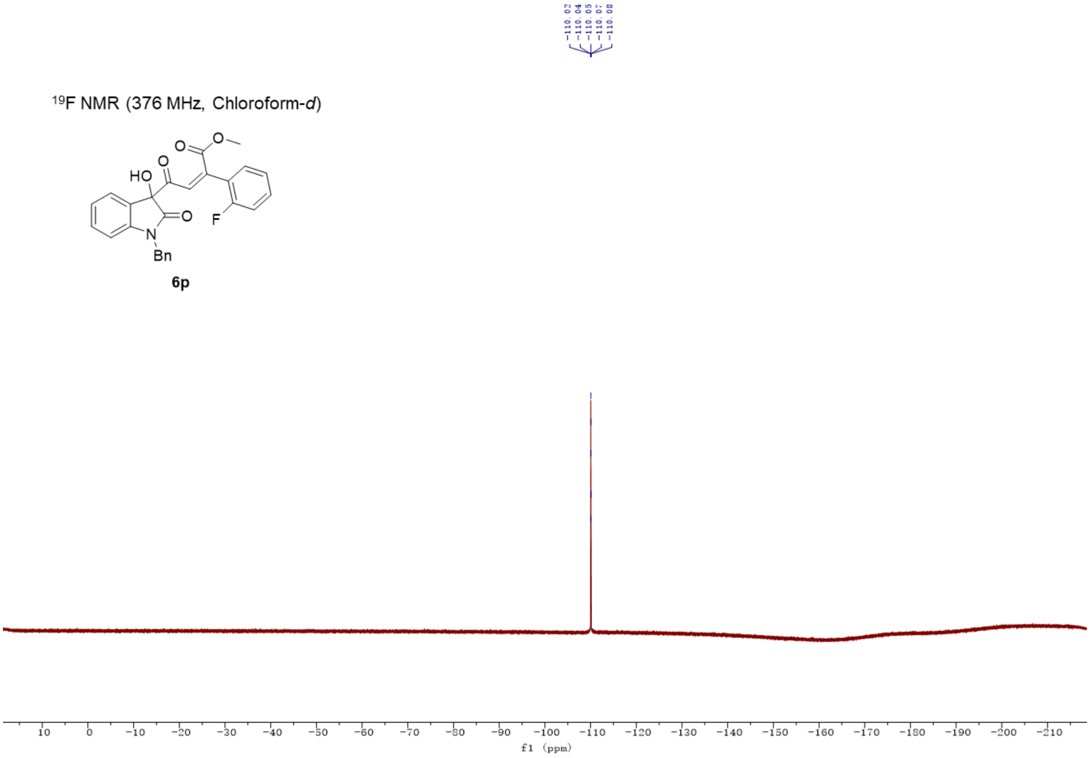
-110.02
-110.04
-110.05
-110.07
-110.08
19F NMR (376 MHz, Chloroform-d)
HO
O
O
O
N
Bn
F
6p
f1 (ppm)

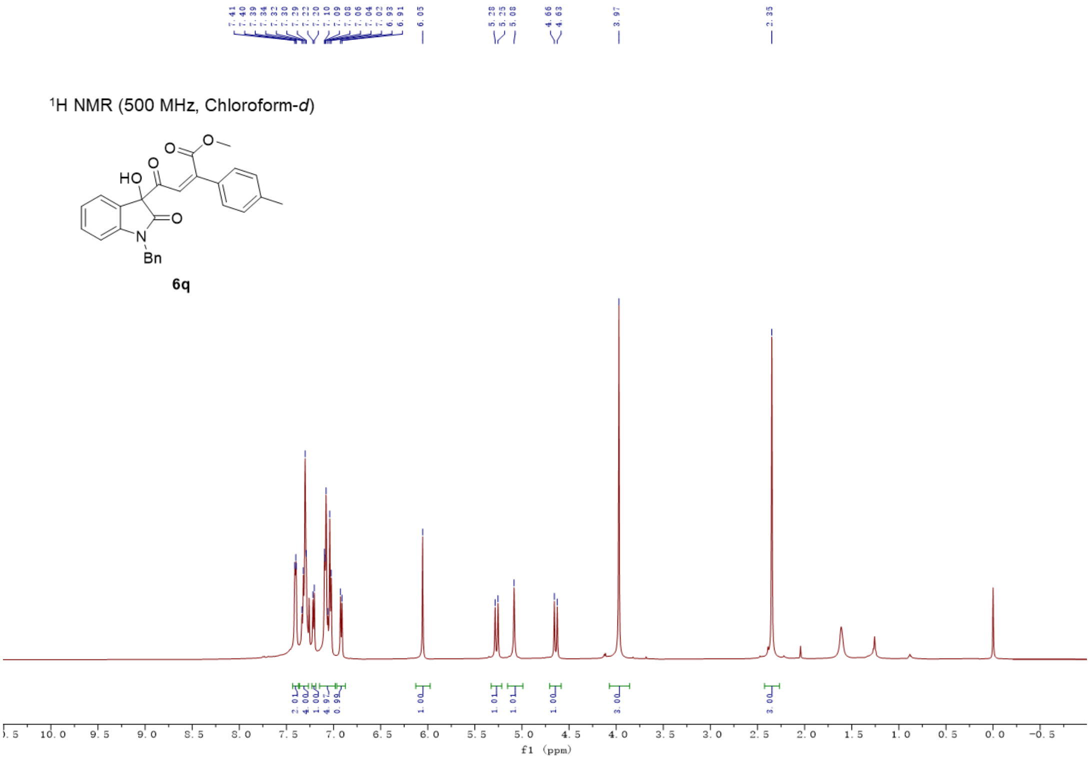

1H NMR (500 MHz, Chloroform-d)
HO
O
O
O
N
Bn
6q
f1 (ppm)

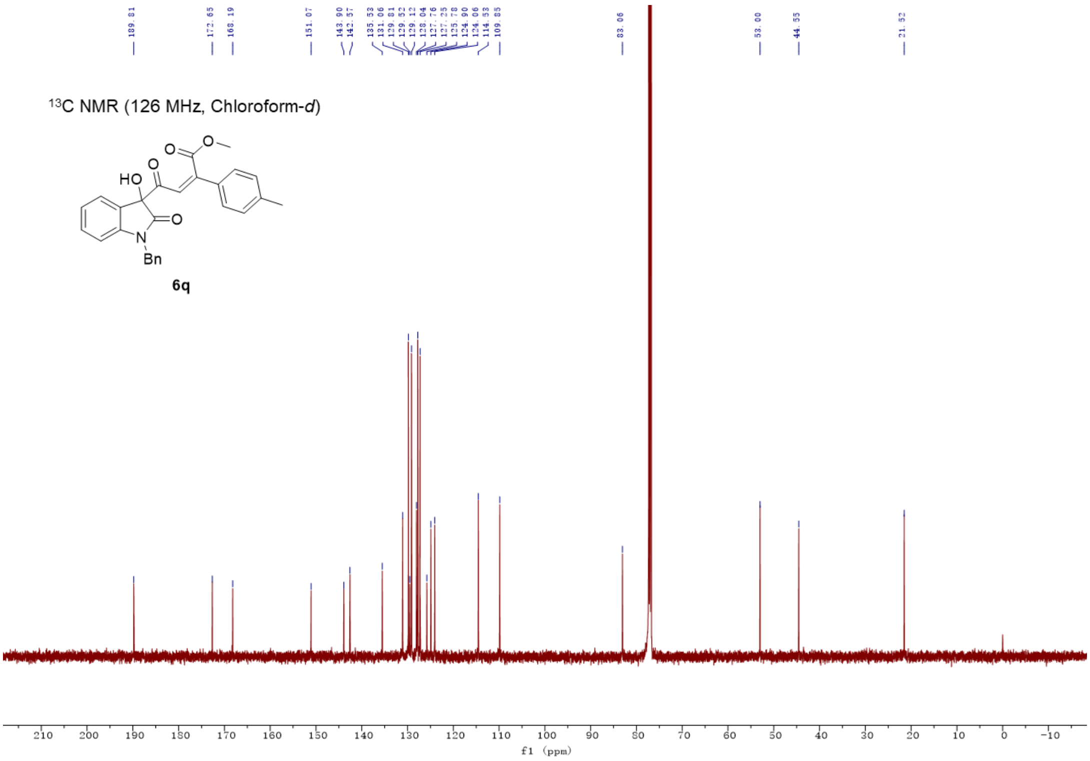

13C NMR (126 MHz, Chloroform-d)
HO
O
O
O
N
Bn
6q
f1 (ppm)

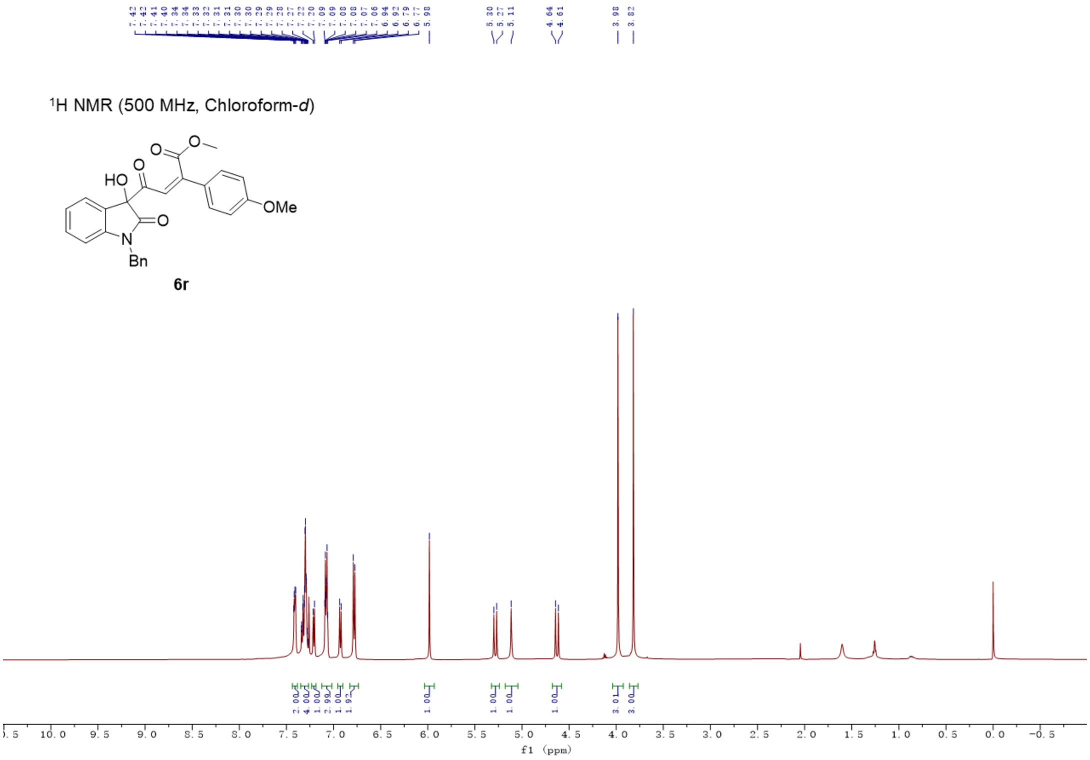
1H NMR (500 MHz, Chloroform-d)
HO
O
O
O
OMe
N
Bn
6r
f1 (ppm)

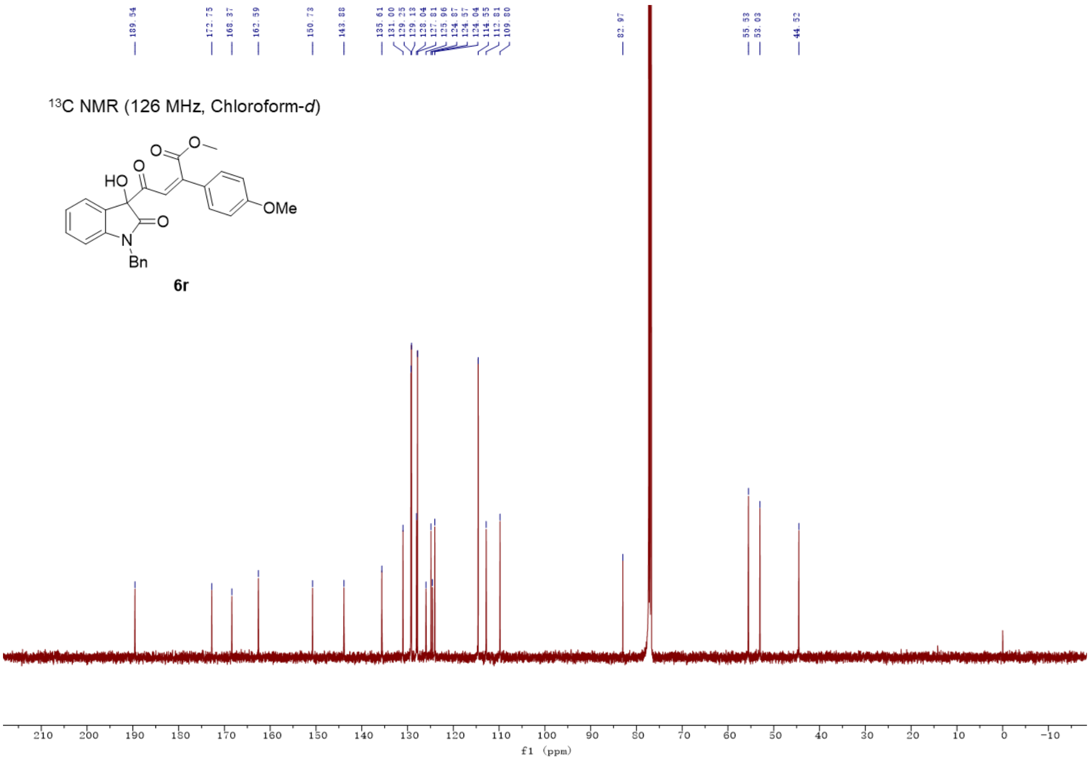
13C NMR (126 MHz, Chloroform-d)
HO
O
O
O
OMe
N
Bn
6r
f1 (ppm)

^{1}H NMR (400 MHz, Chloroform-*d*)

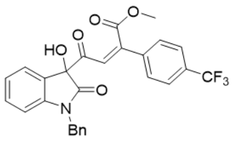

6s

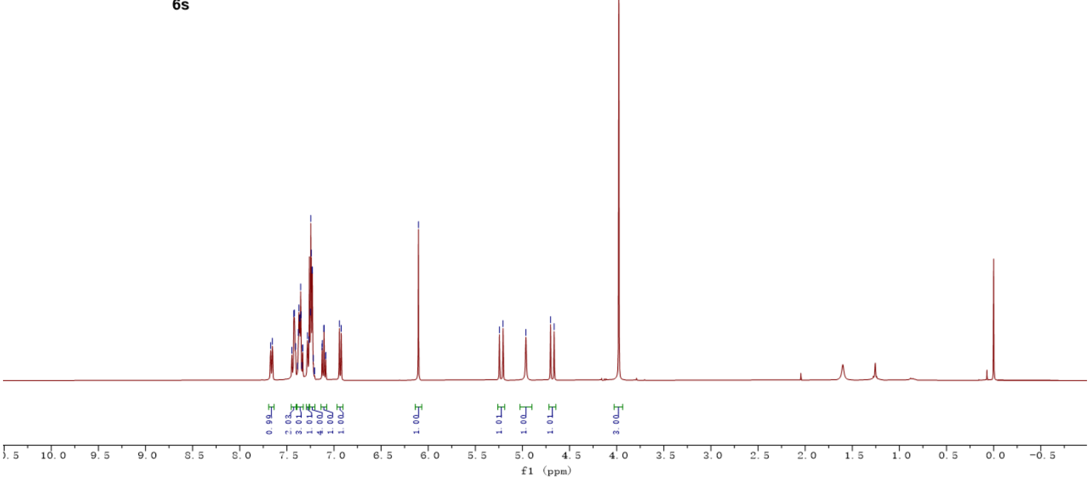

^{13}C NMR (126 MHz, Chloroform-*d*)

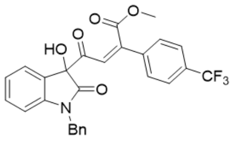

6s

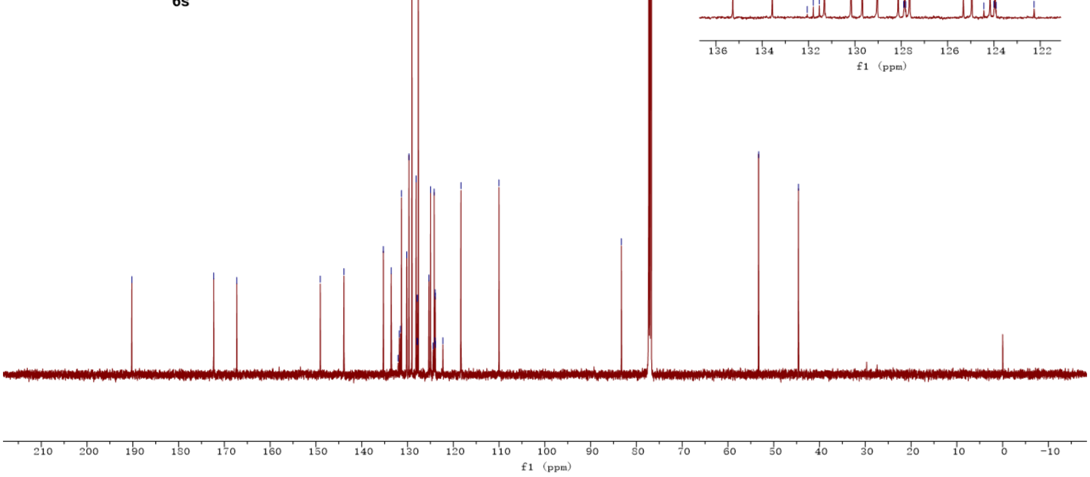

^{19}F NMR (376 MHz, Chloroform-*d*)

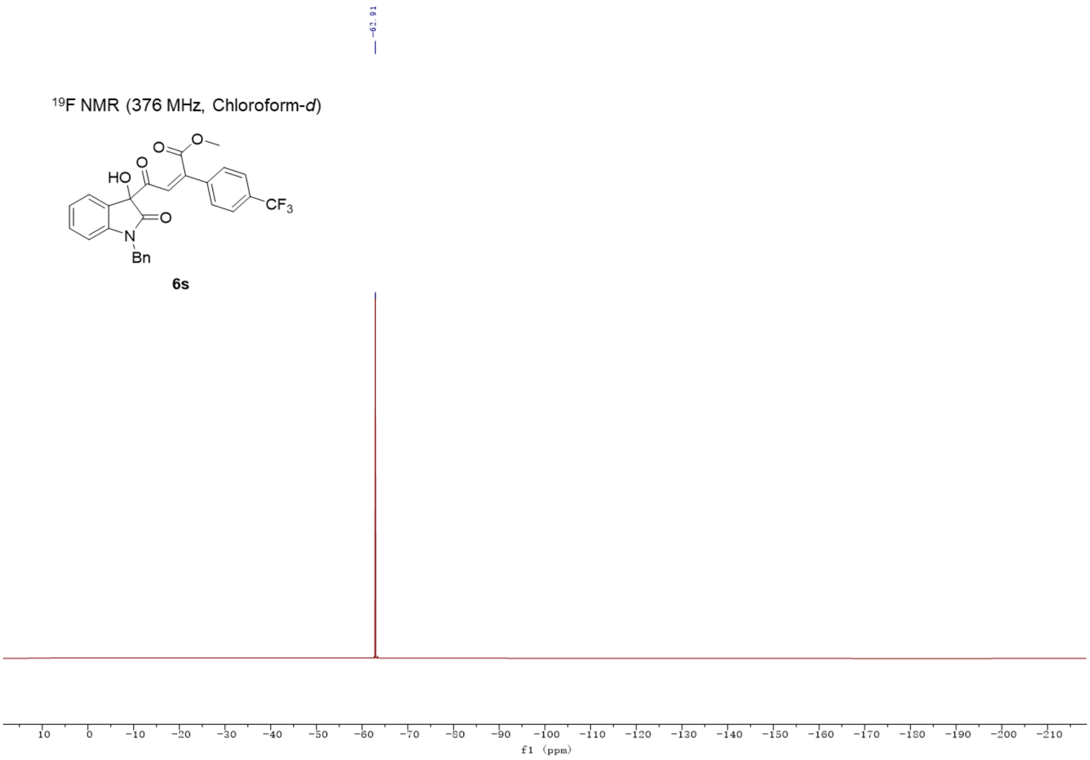

^{1}H NMR (500 MHz, Chloroform-*d*)

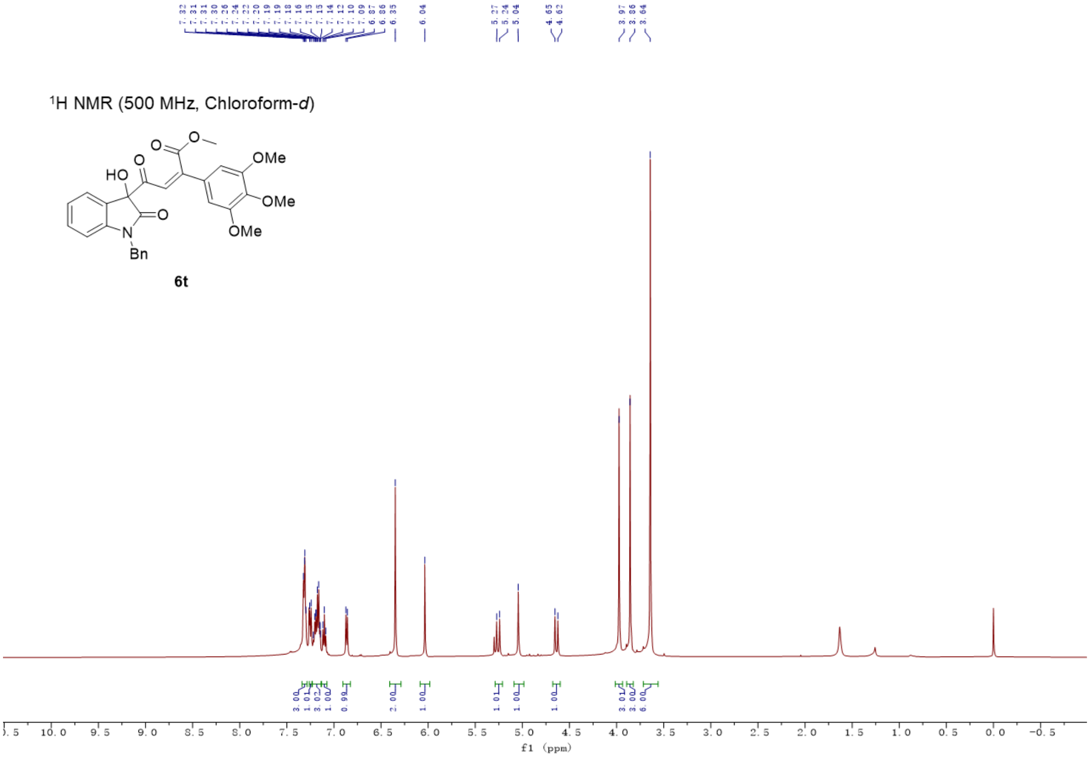

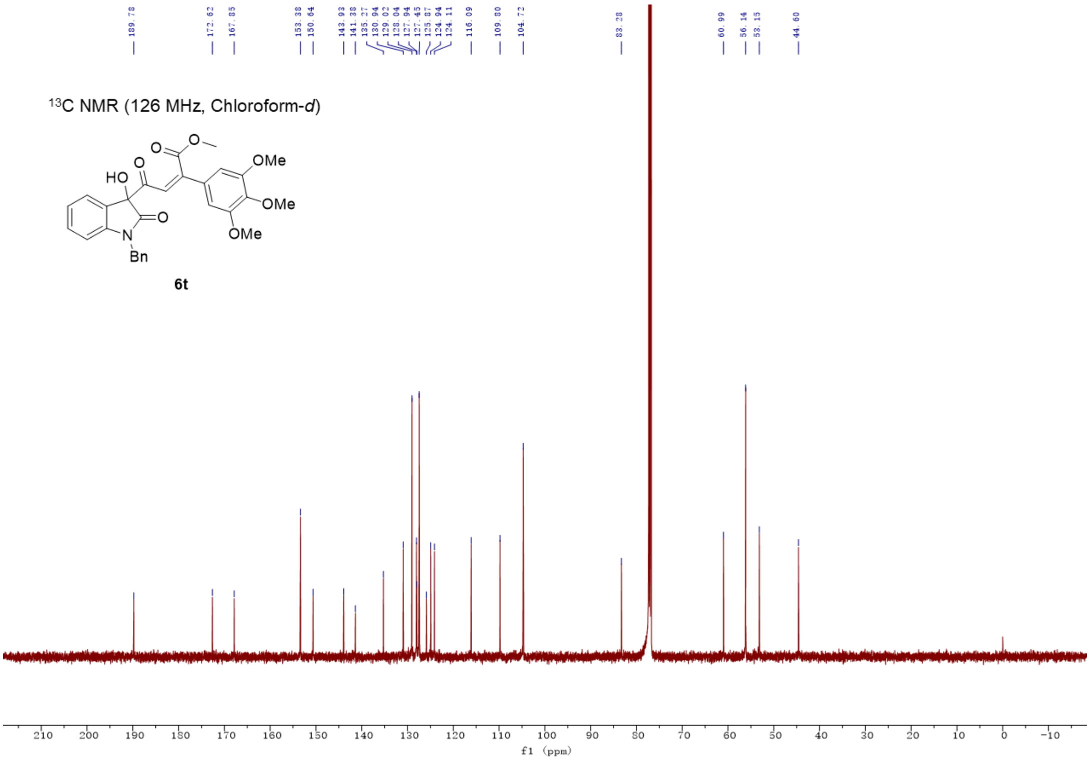
189.78
172.62
167.85
153.38
150.64
143.93
141.38
135.27
130.94
129.02
128.04
127.94
127.45
125.87
124.94
124.11
116.09
109.80
104.72
83.28
60.99
56.14
53.15
44.60
13C NMR (126 MHz, Chloroform-d)
HO
O
OMe
N
Bn
6t
f1 (ppm)

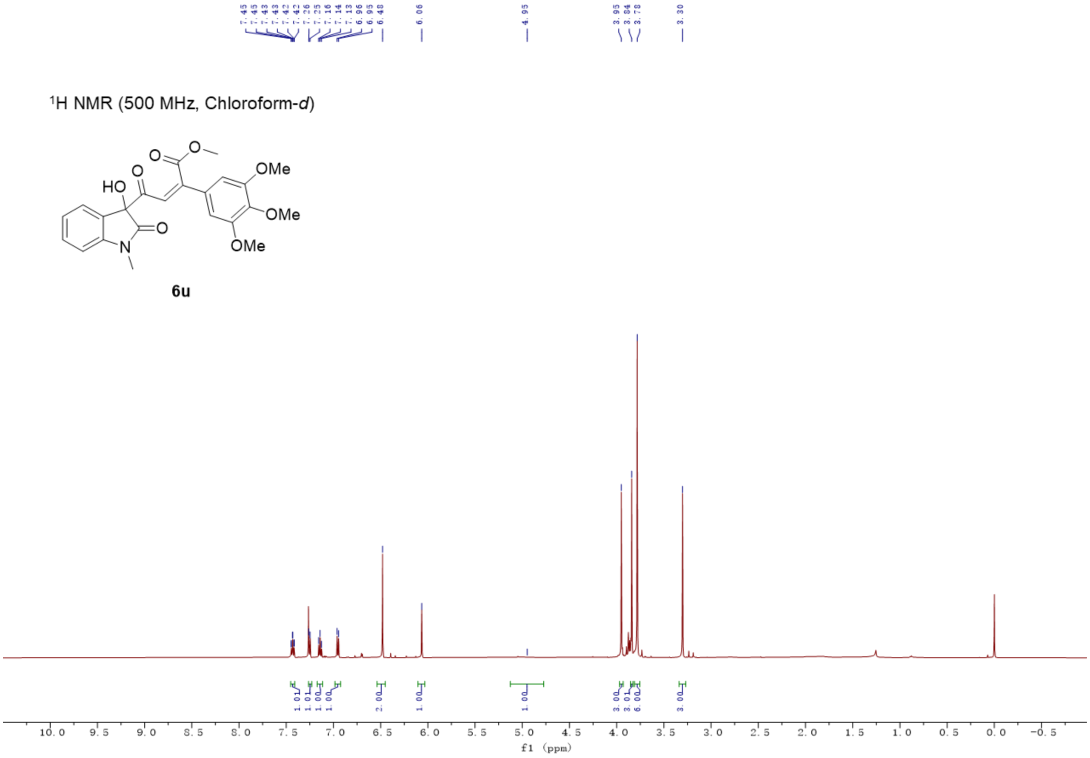
7.45
7.45
7.43
7.43
7.42
7.26
7.25
7.16
7.14
7.13
6.96
6.95
6.48
6.06
4.95
3.95
3.84
3.78
3.30
1H NMR (500 MHz, Chloroform-d)
HO
O
OMe
N
6u
f1 (ppm)

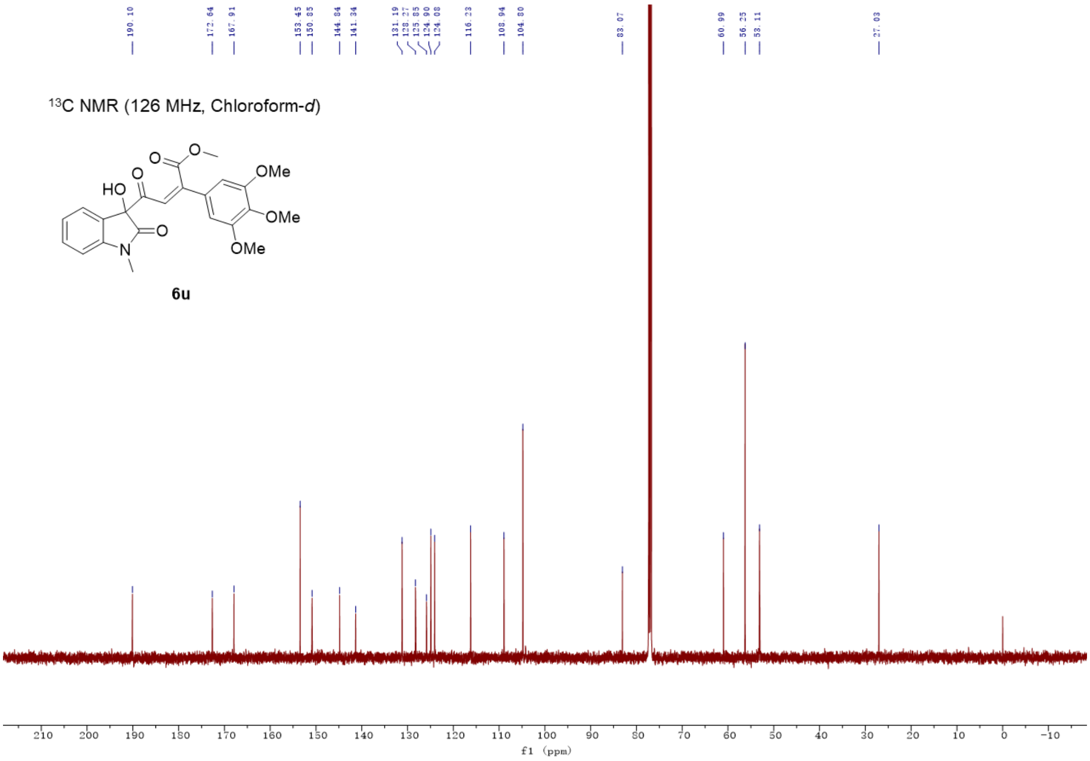
190.10
172.64
167.91
153.45
150.85
144.84
141.34
131.19
128.27
125.85
124.90
124.08
116.23
108.94
104.80
83.07
60.99
56.25
53.11
27.03
13C NMR (126 MHz, Chloroform-d)
HO
O
O
O
OMe
OMe
OMe
N
6u
f1 (ppm)

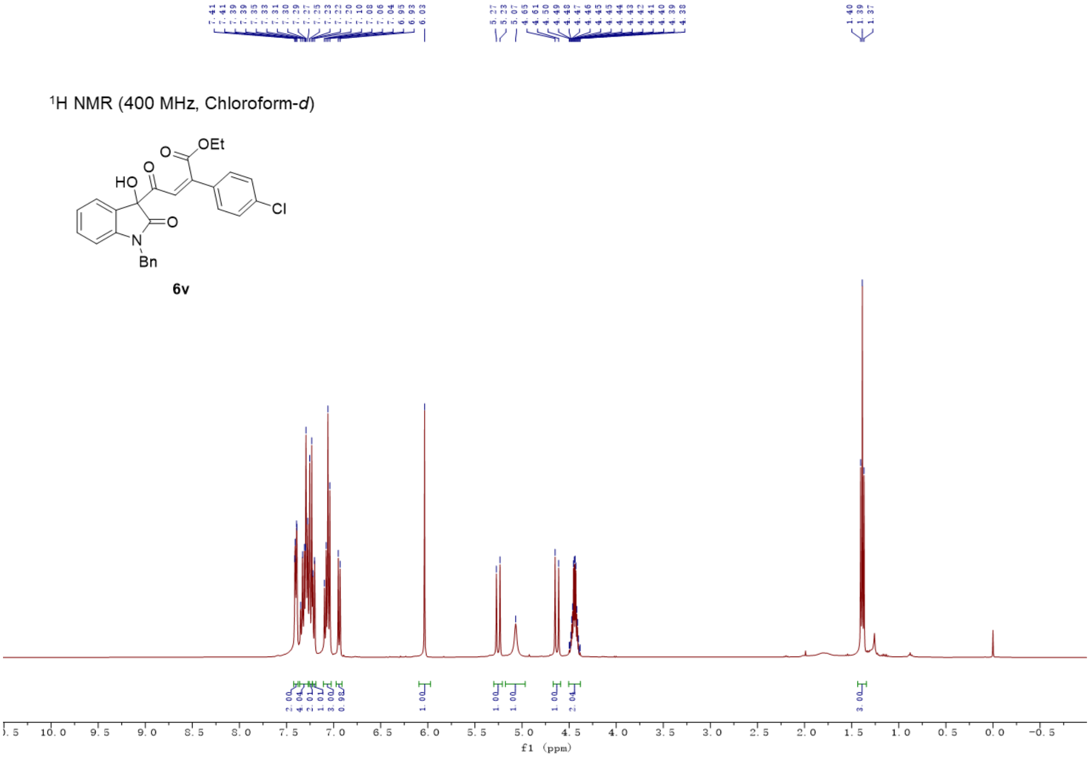
1H NMR (400 MHz, Chloroform-d)
OEt
O
O
HO
Cl
O
N
Bn
6v
f1 (ppm)

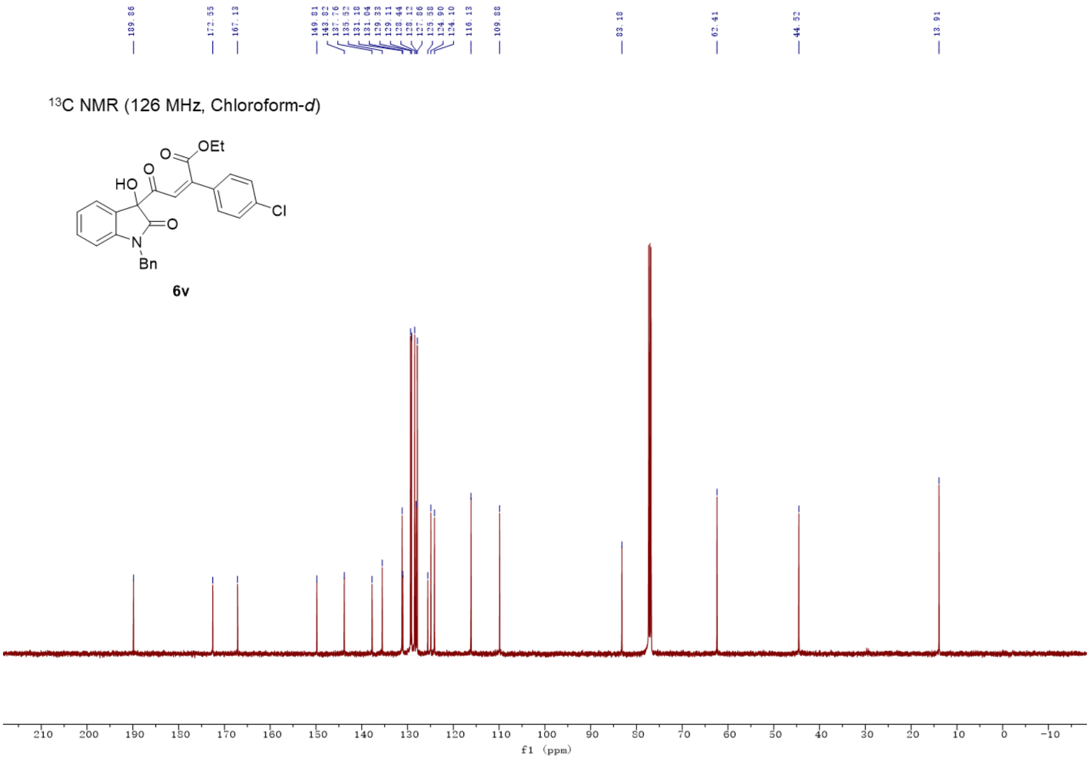
13C NMR (126 MHz, Chloroform-d)
OEt
HO
O
N
Bn
Cl
6v
f1 (ppm)

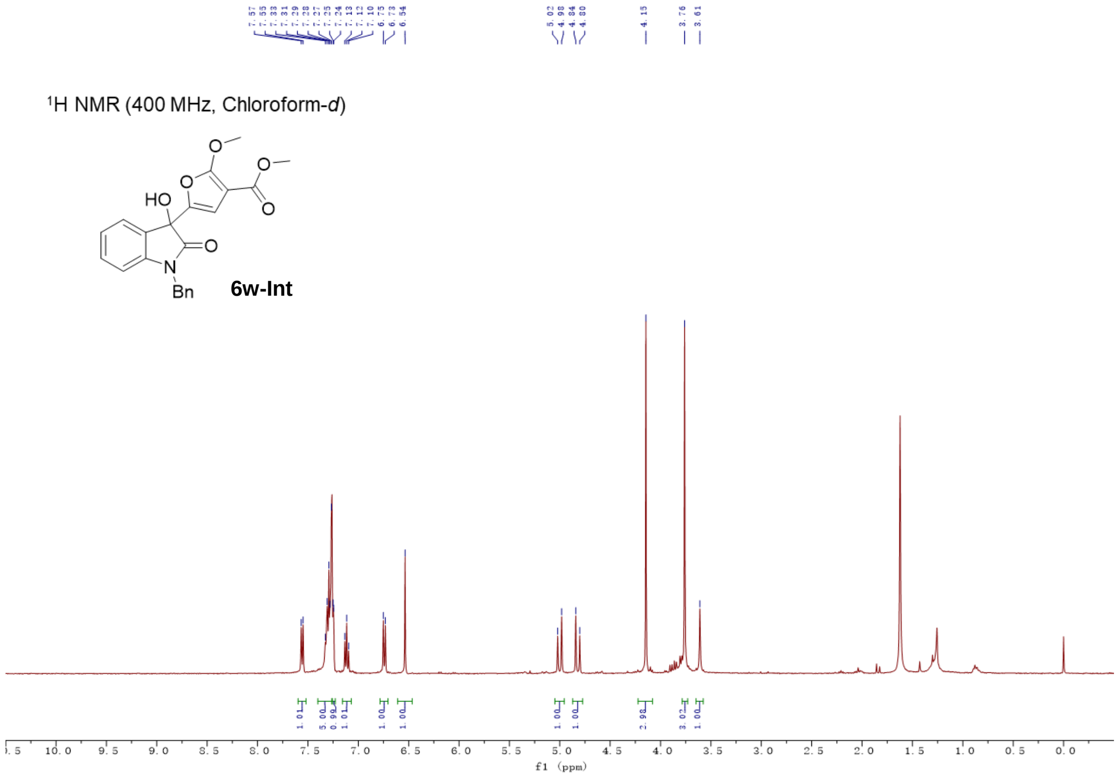
1H NMR (400 MHz, Chloroform-d)
HO
O
N
Bn
6w-Int
f1 (ppm)

^{13}C NMR (126 MHz, Chloroform-*d*)

6w-Int

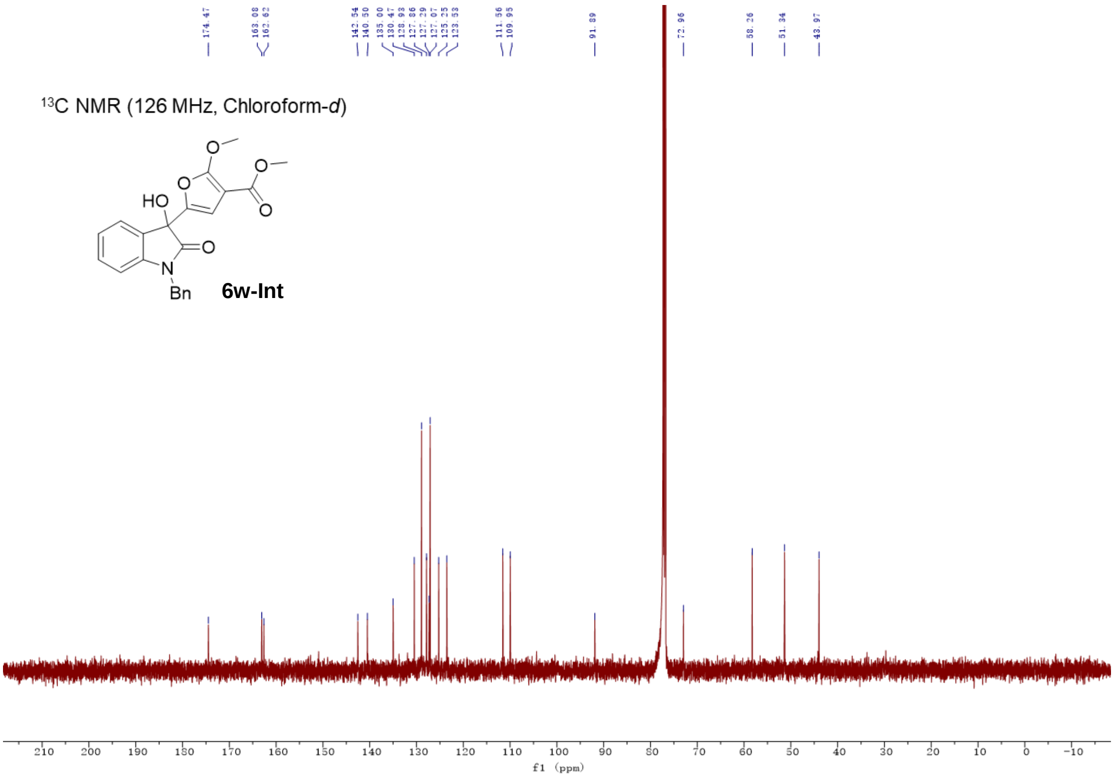

^{1}H NMR (400 MHz, Chloroform-*d*)

6x-H_2O

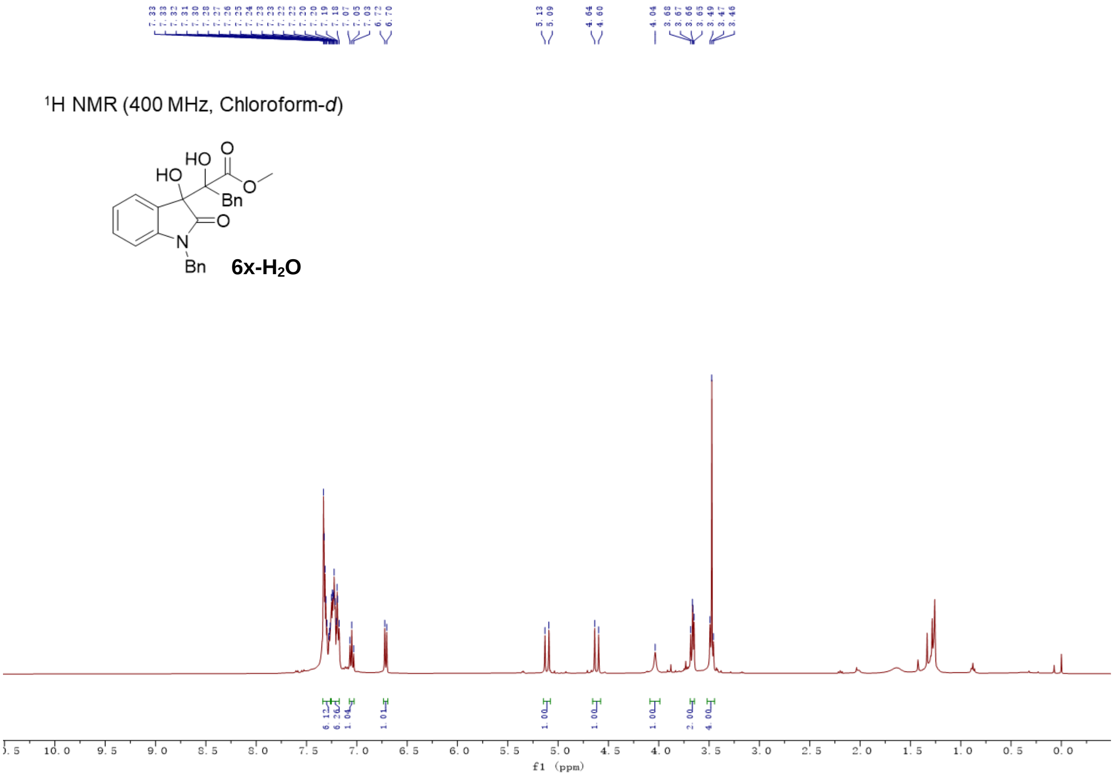

6x-H_2O

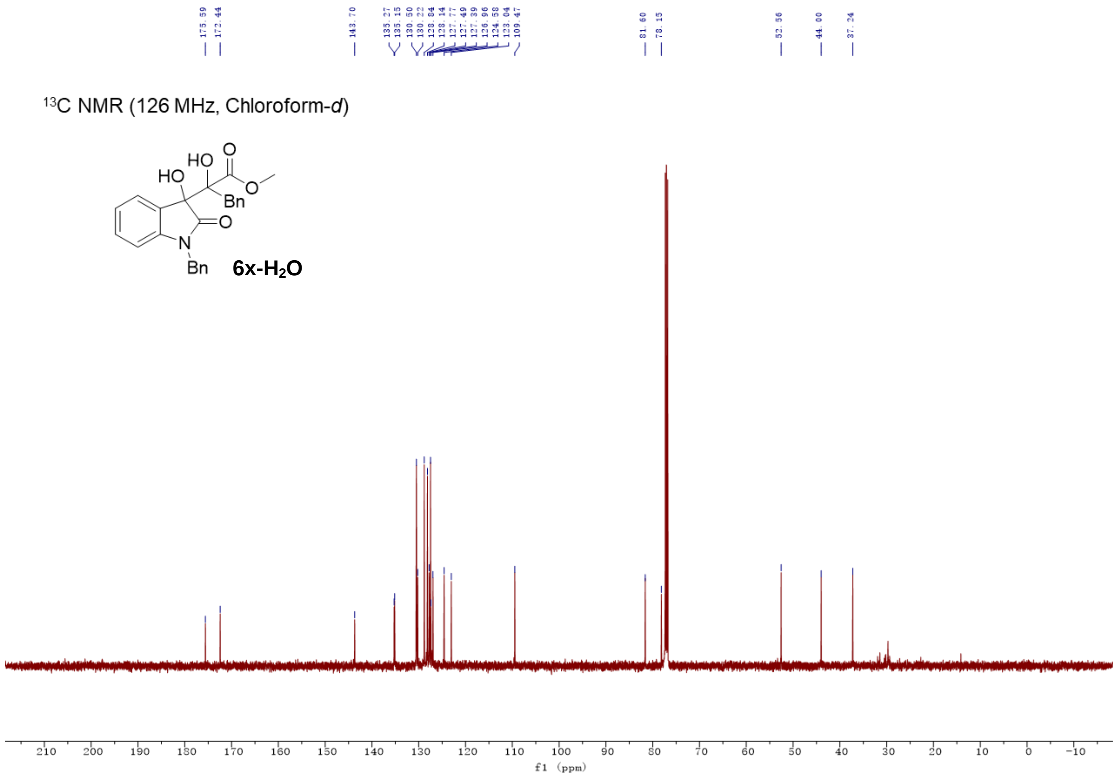

13C NMR (126 MHz, Chloroform-d)
HO
HO
O
O
Bn
O
N
Bn
6x-H2O
f1 (ppm)

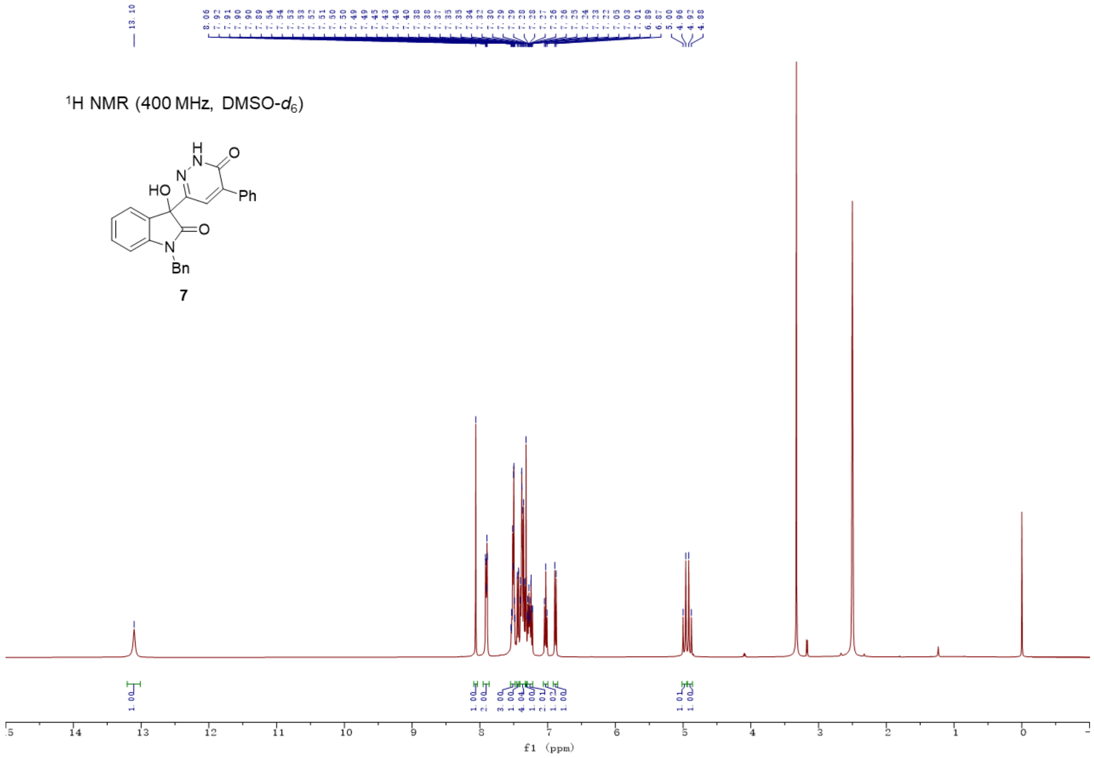

1H NMR (400 MHz, DMSO-d6)
H
N
N
O
HO
Ph
O
N
Bn
7
f1 (ppm)

^{13}C NMR (101 MHz, DMSO-d_6)

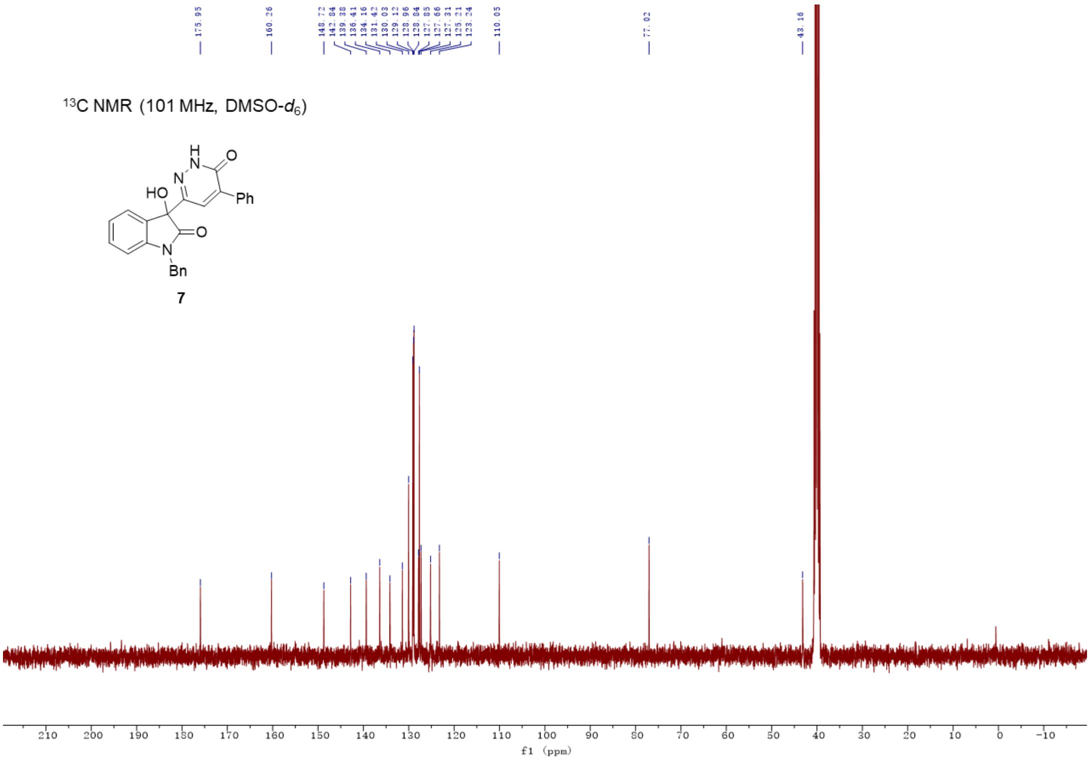

^{1}H NMR (500 MHz, DMSO-d_6)

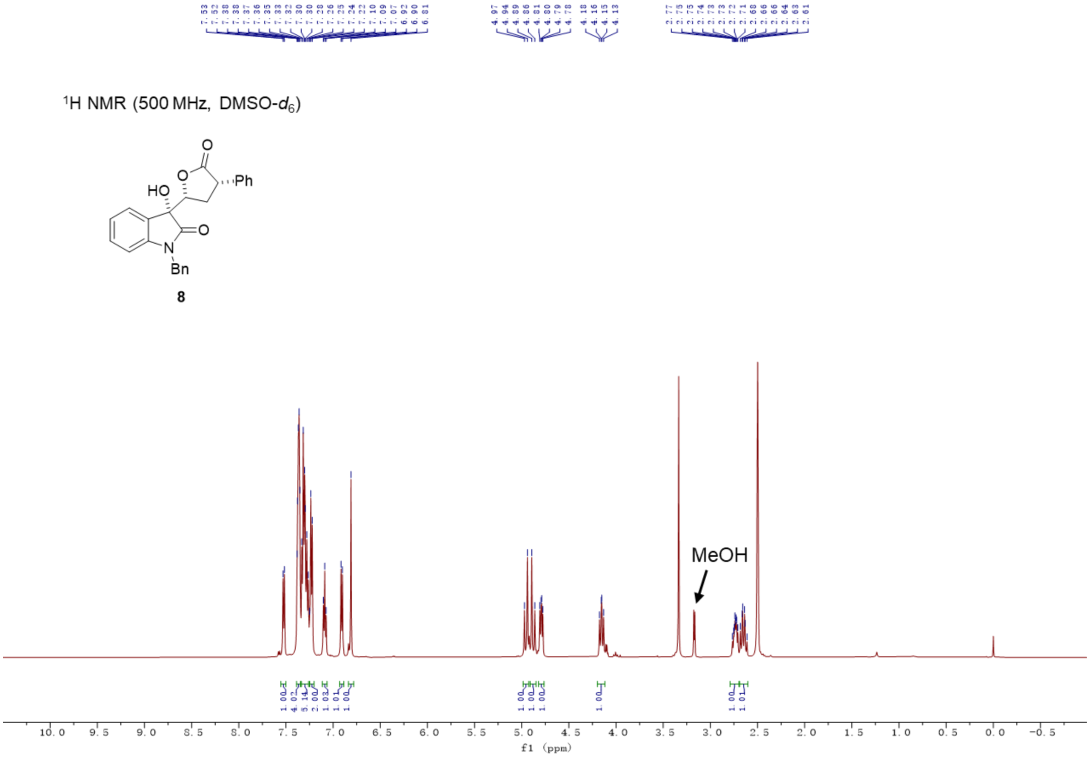

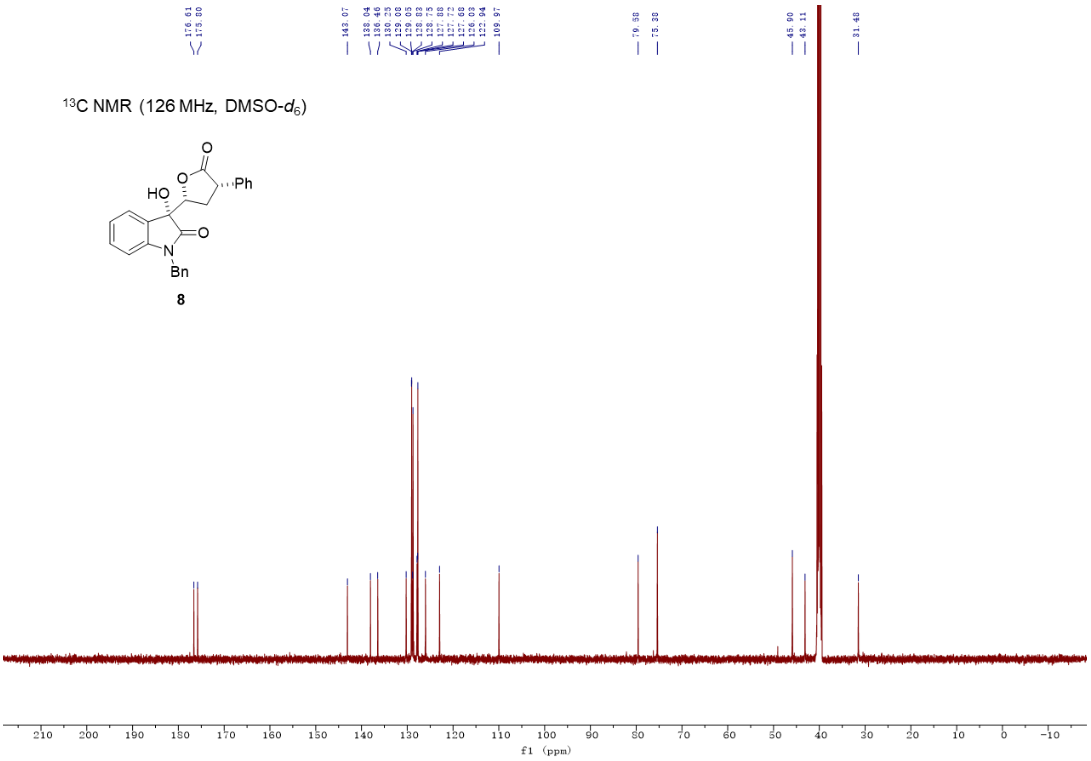

COSY spectrum for compound **8**

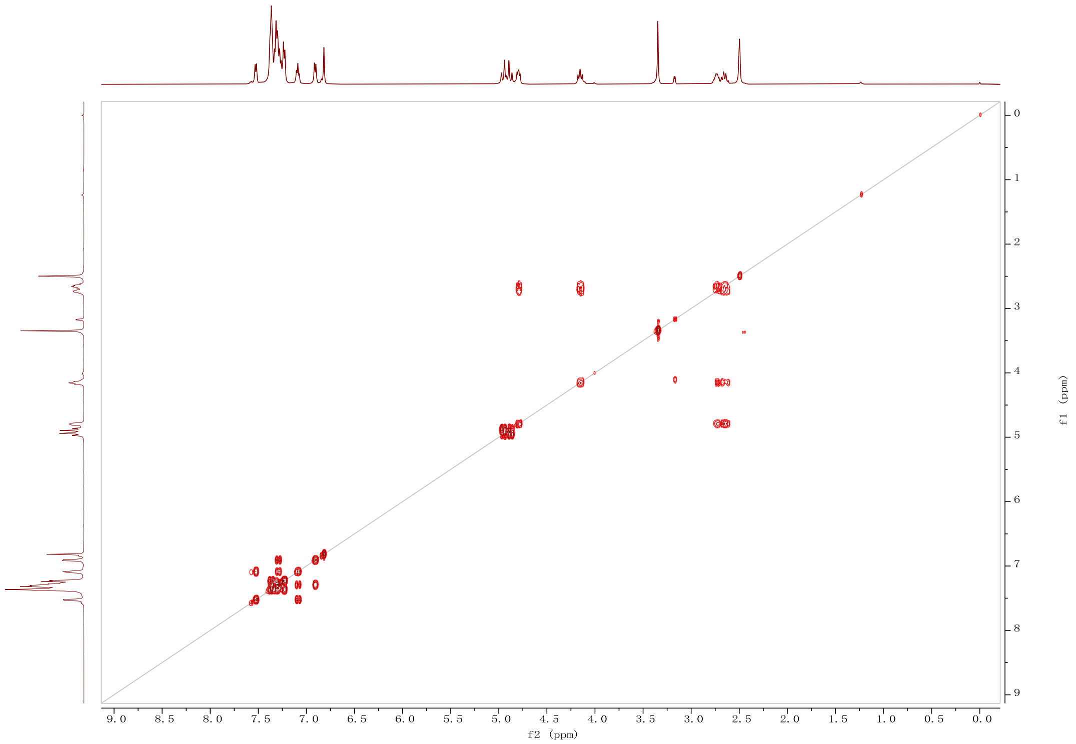

1D-NOE spectrum for **8** (500 MHz, DMSO-d_6)

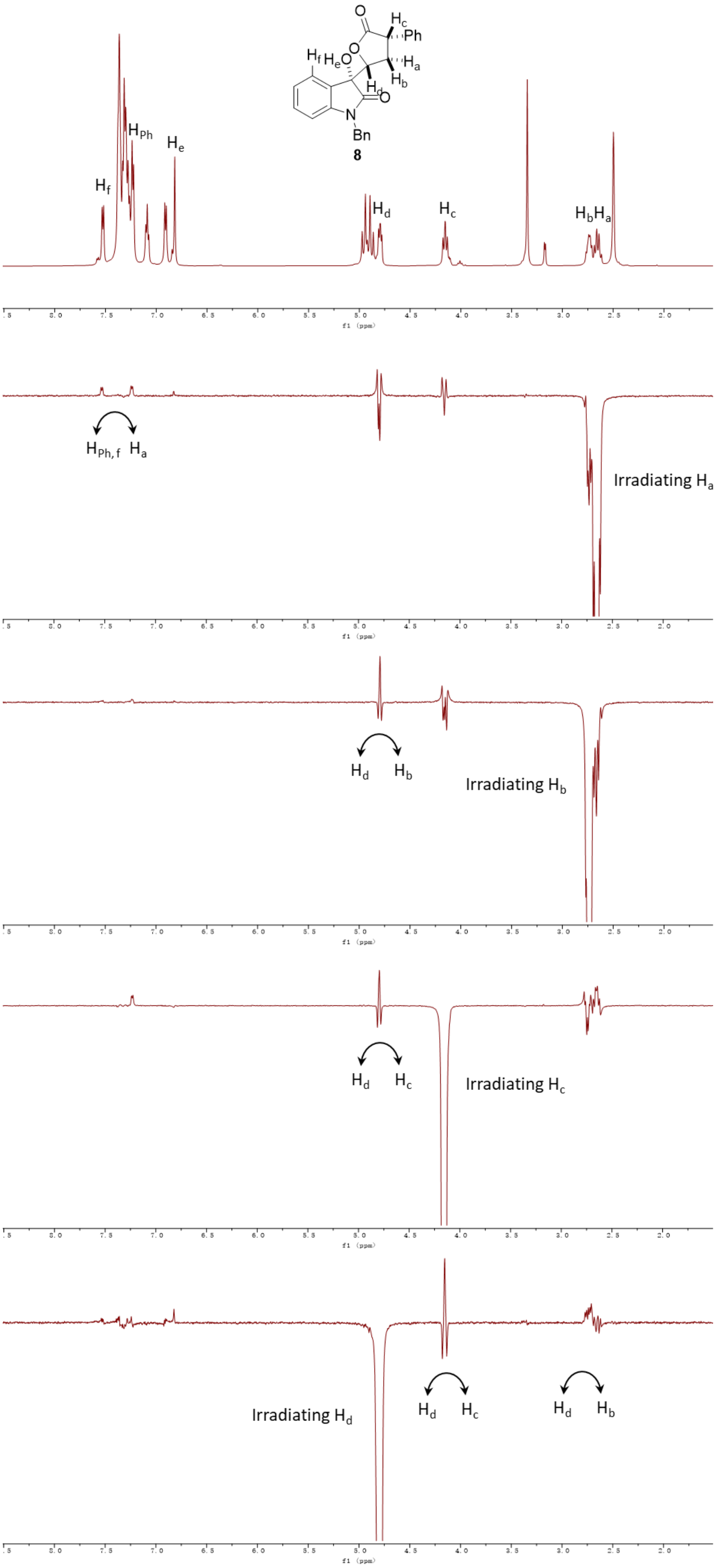

1D-NOE spectrum for **8** (500 MHz, DMSO-d_6)

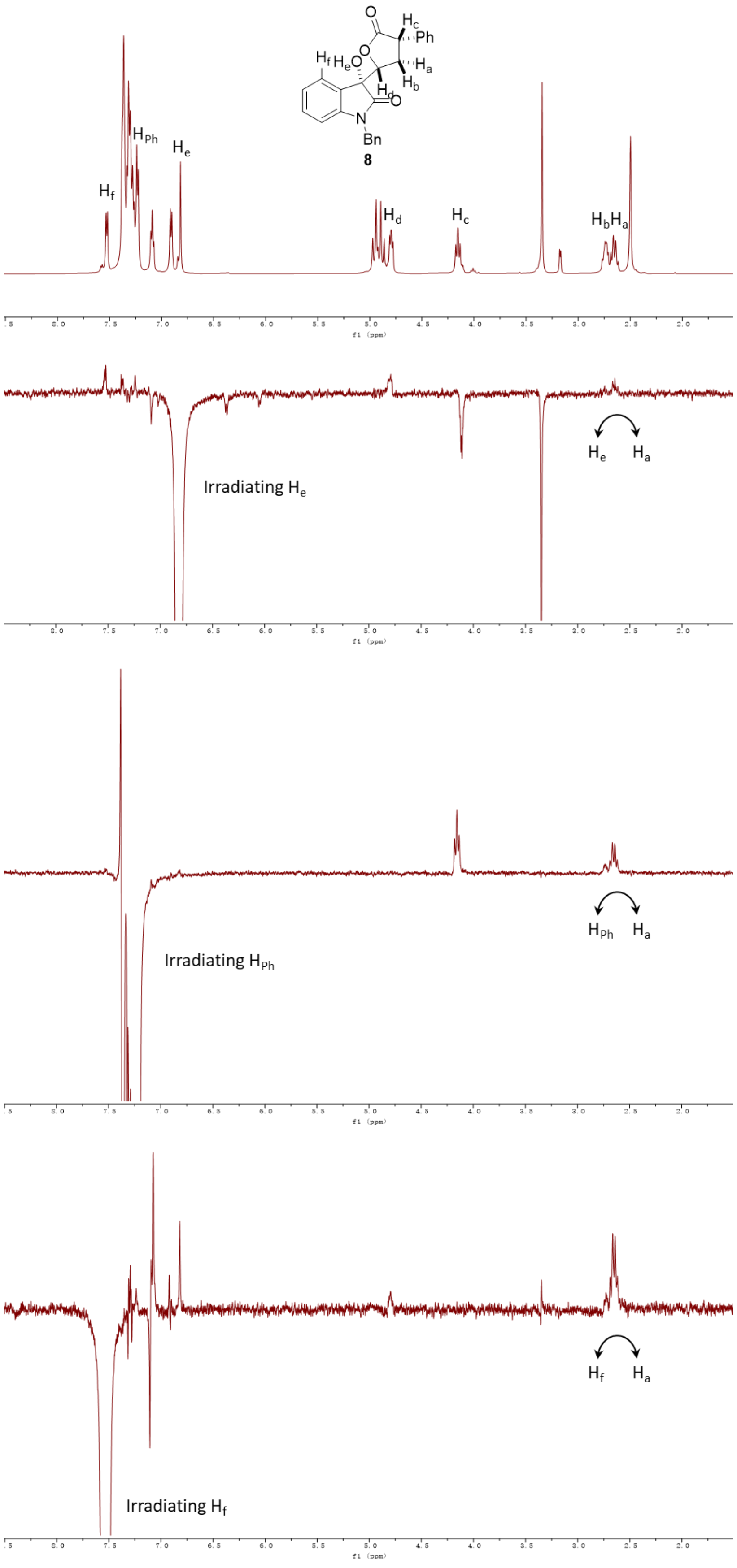

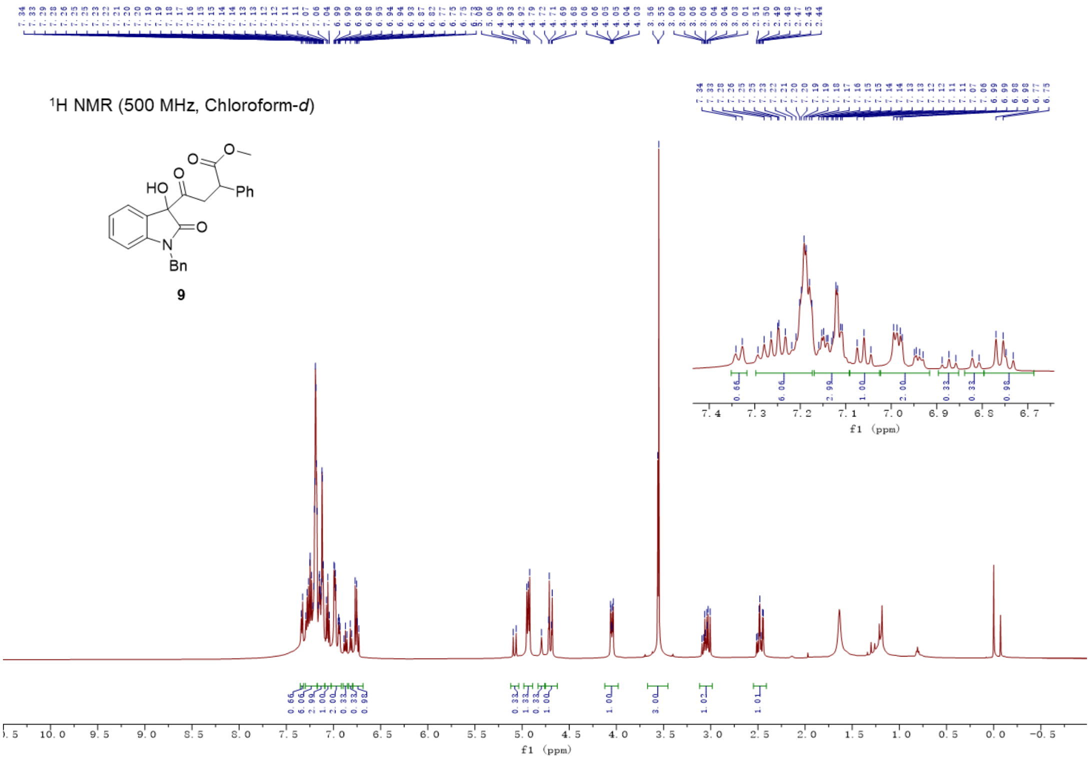

^{1}H NMR (500 MHz, Chloroform-*d*)
HO
O
O
O
Ph
N
O
Bn
9
f1 (ppm)

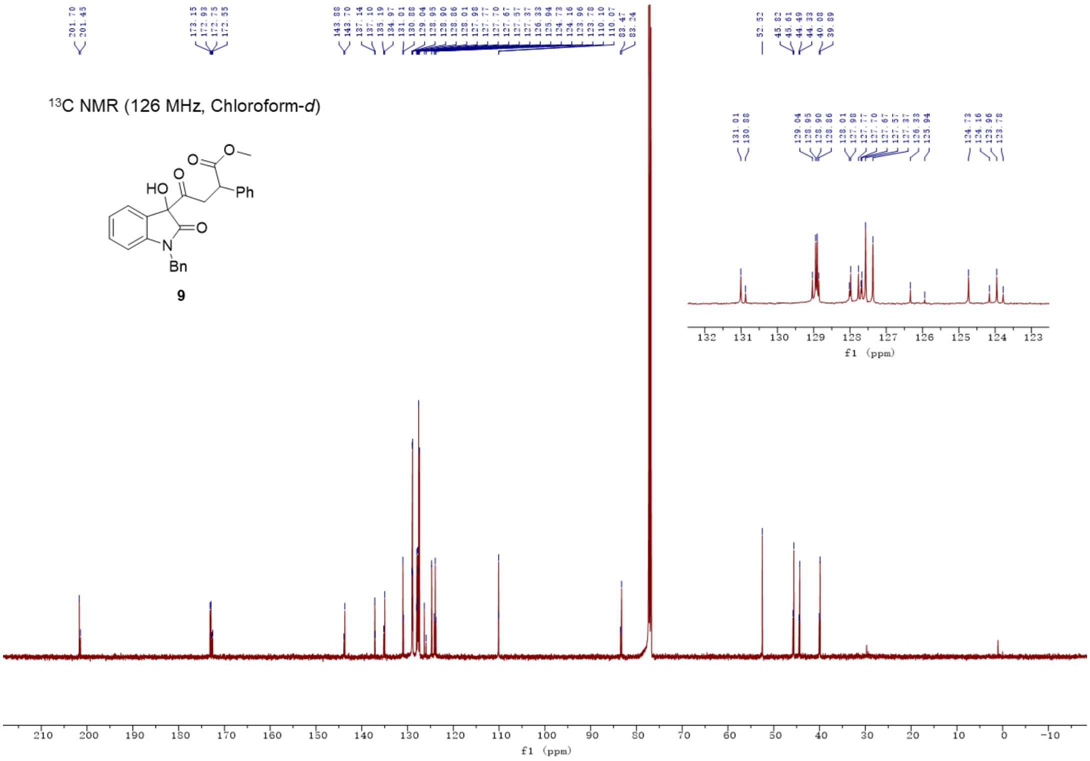

^{13}C NMR (126 MHz, Chloroform-*d*)
HO
O
O
O
Ph
N
O
Bn
9
f1 (ppm)

^{1}H NMR (500 MHz, DMSO-d_6)

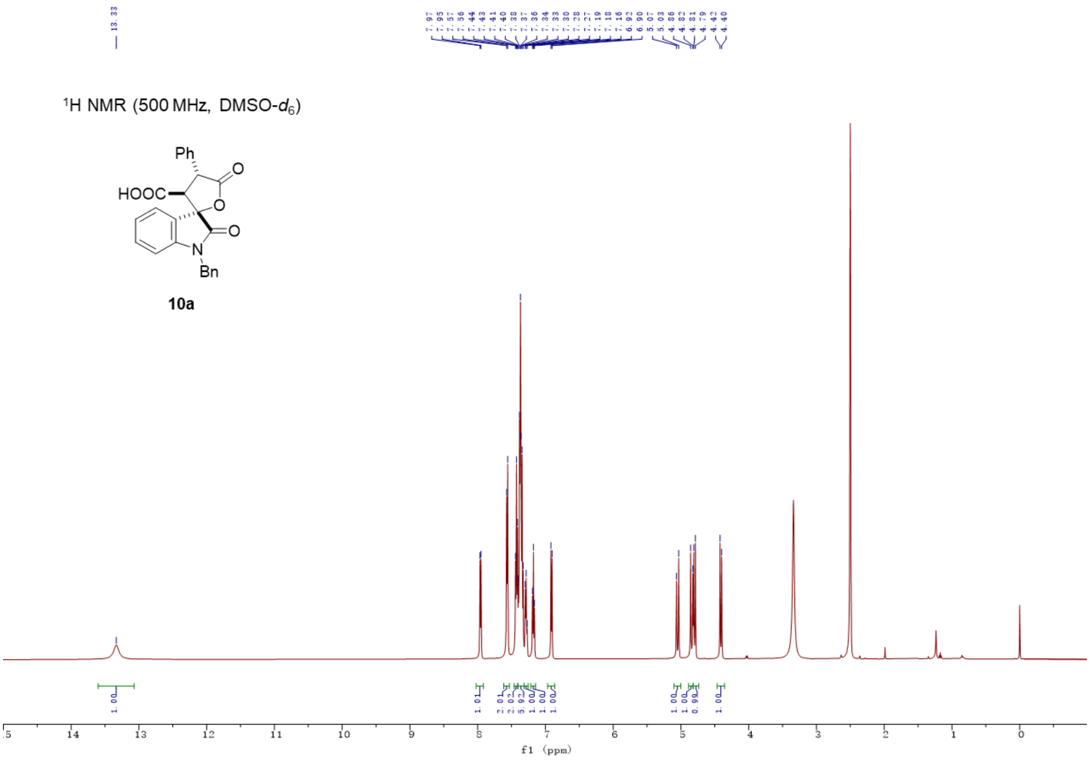

10a

^{13}C NMR (126 MHz, DMSO-d_6)

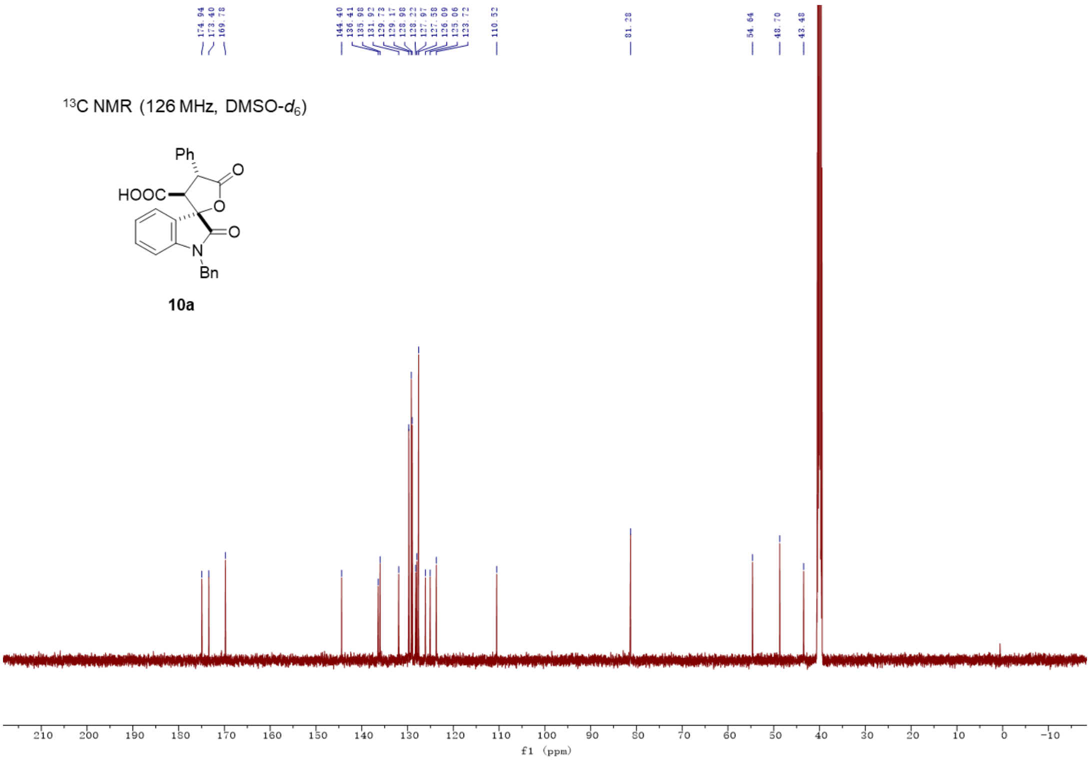

10a

DEPT 135 spectrum for compound **10a** (126 MHz, DMSO-d_6)

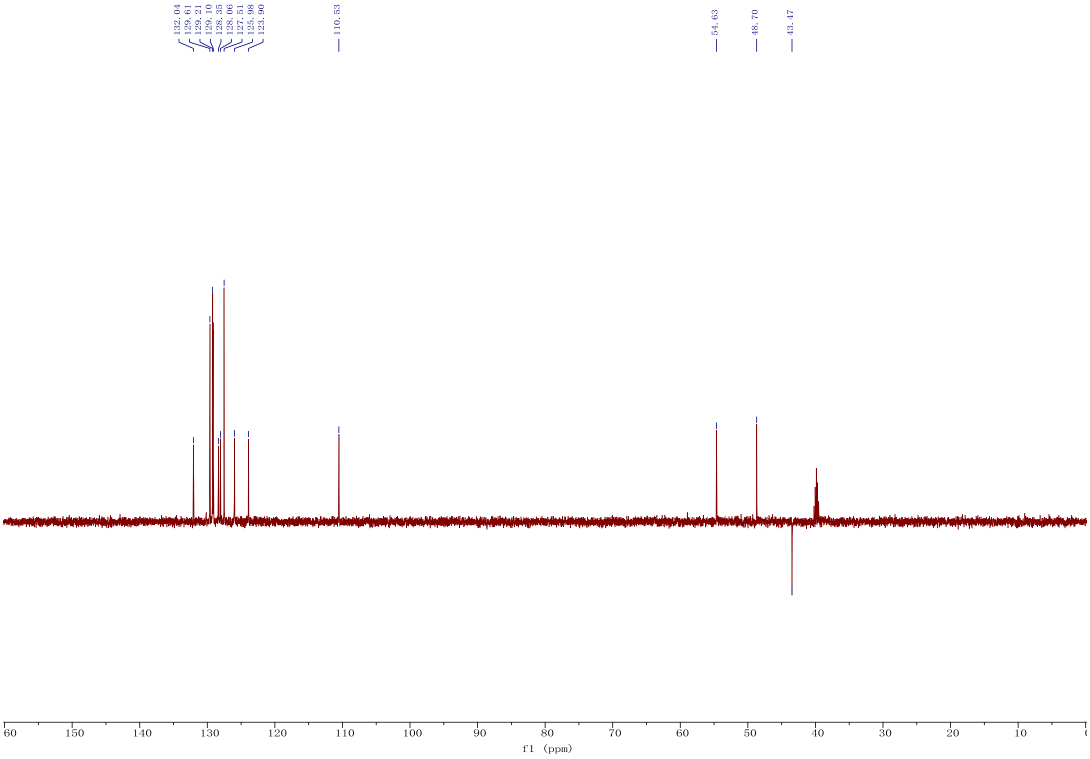

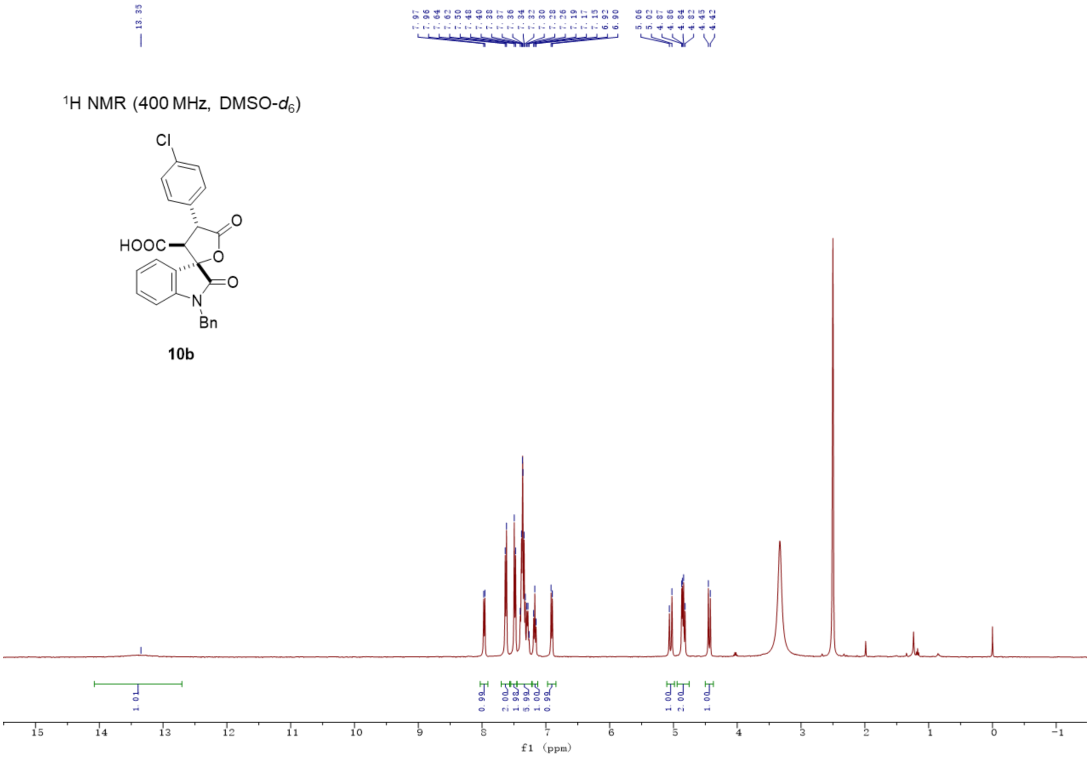

^{1}H NMR (400 MHz, DMSO-d_6)

10b

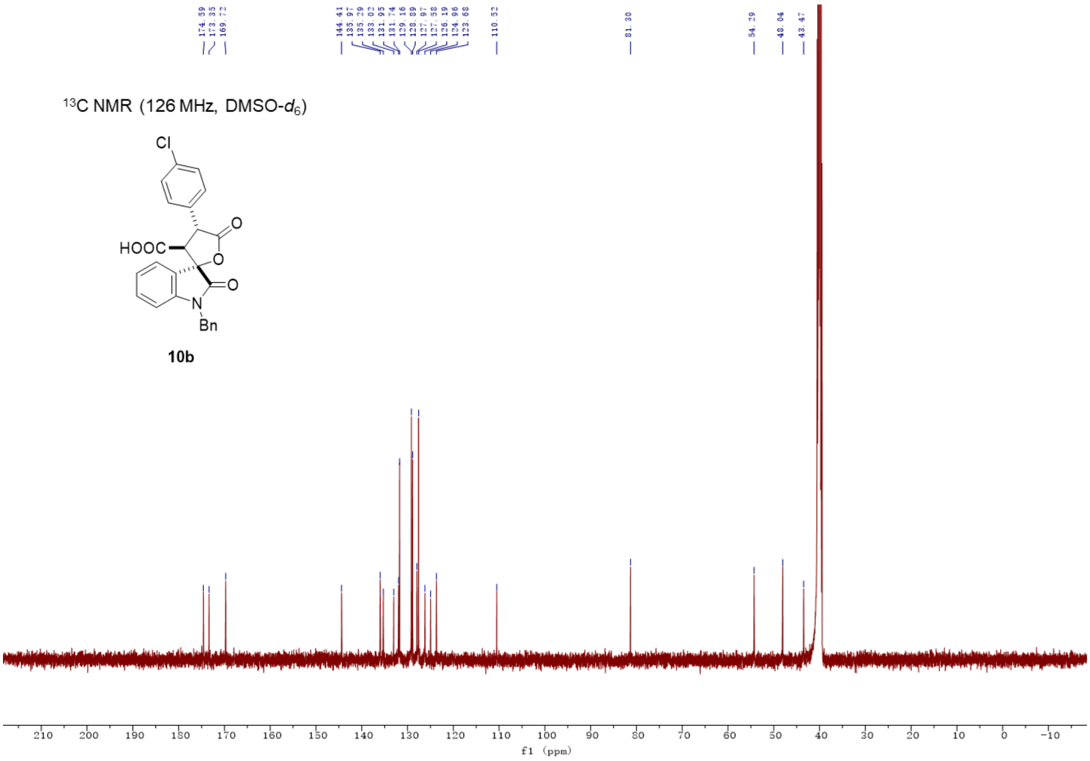

^{13}C NMR (126 MHz, DMSO-d_6)
Cl
HOOC
O
O
O
N
Bn
10b
f1 (ppm)

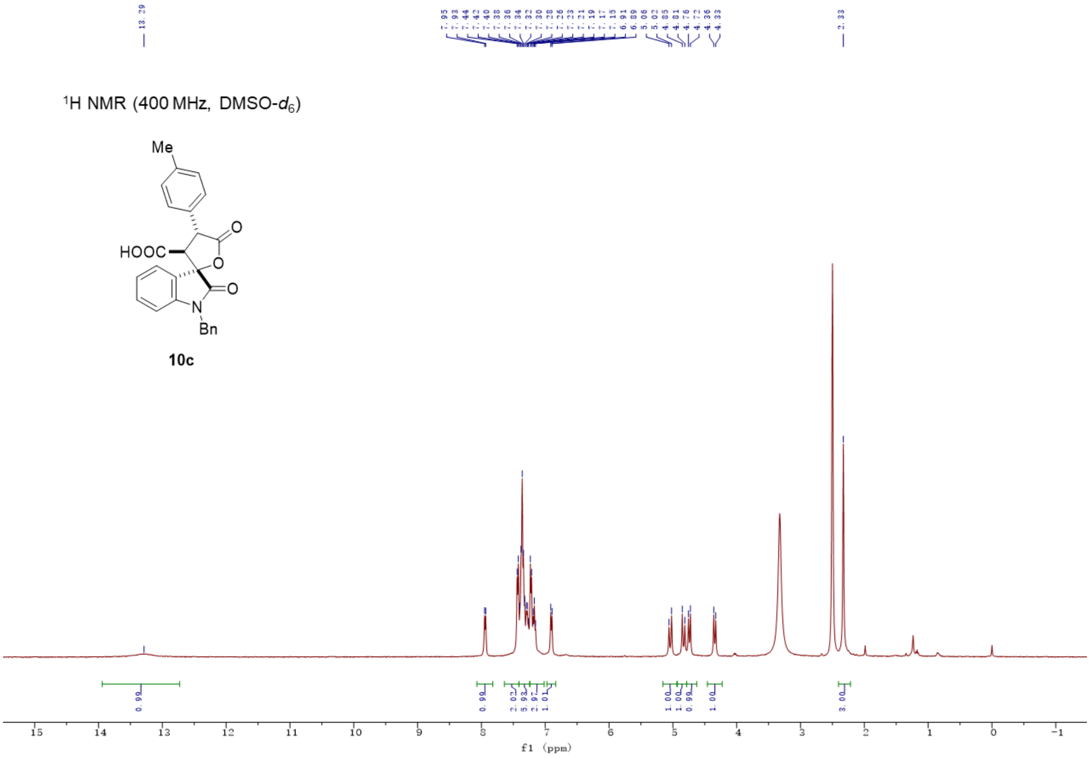

^{1}H NMR (400 MHz, DMSO-d_6)
Me
HOOC
O
O
O
N
Bn
10c
f1 (ppm)

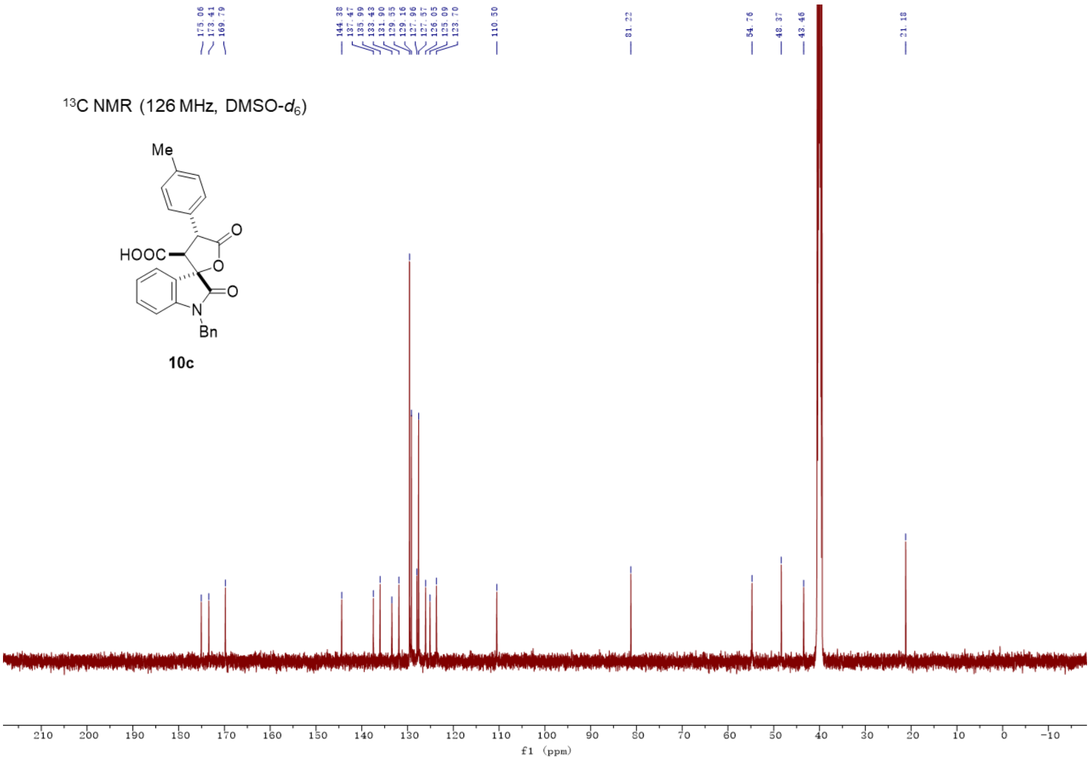
175.06
173.41
169.79
144.38
137.47
135.99
133.43
131.90
129.55
129.16
127.96
127.57
126.05
125.09
123.70
110.50
81.22
54.76
48.37
43.46
21.18
^{13}C NMR (126 MHz, DMSO-d_6)
Me
HOOC
O
N
Bn
10c
f1 (ppm)

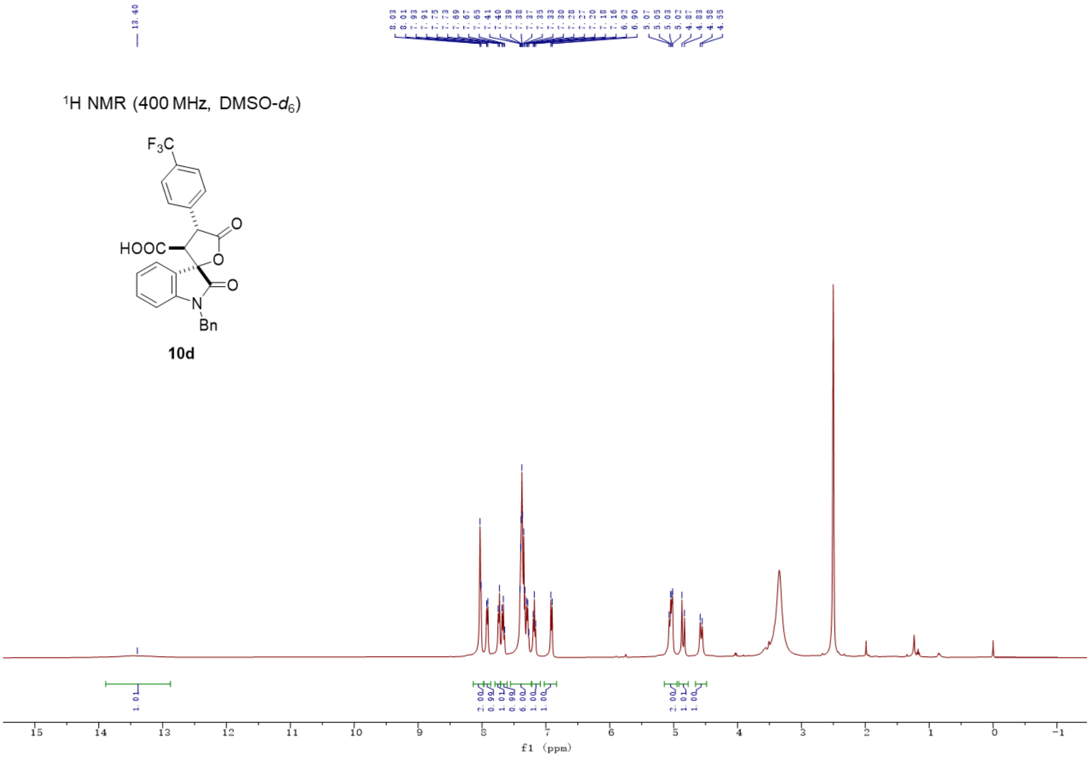
13.40
^{1}H NMR (400 MHz, DMSO-d_6)
F_3C
HOOC
O
N
Bn
10d
f1 (ppm)

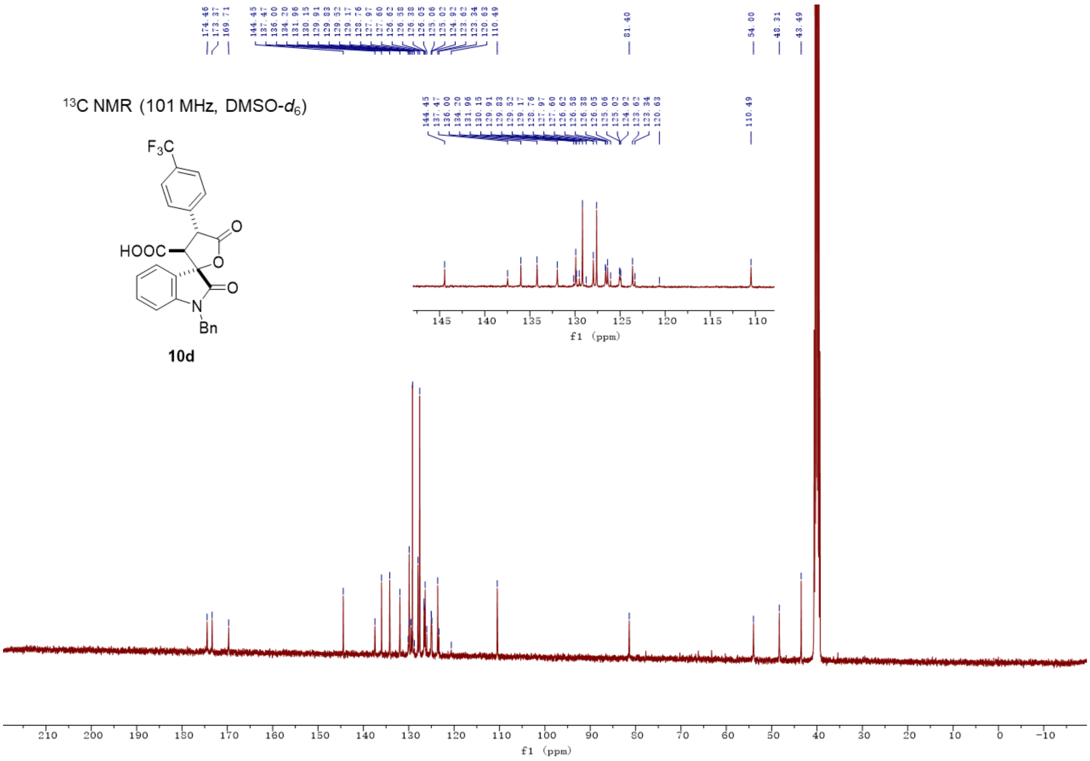
174.46
173.37
169.71
144.45
137.47
136.00
134.20
131.96
130.15
129.91
129.83
129.52
129.17
128.76
127.97
127.60
126.62
126.58
126.38
126.05
125.06
125.02
124.92
123.62
123.34
120.63
110.49
81.40
54.00
48.31
43.49
^{13}C NMR (101 MHz, DMSO-d_6)
F$_3$C
HOOC
O
N
Bn
10d
f1 (ppm)

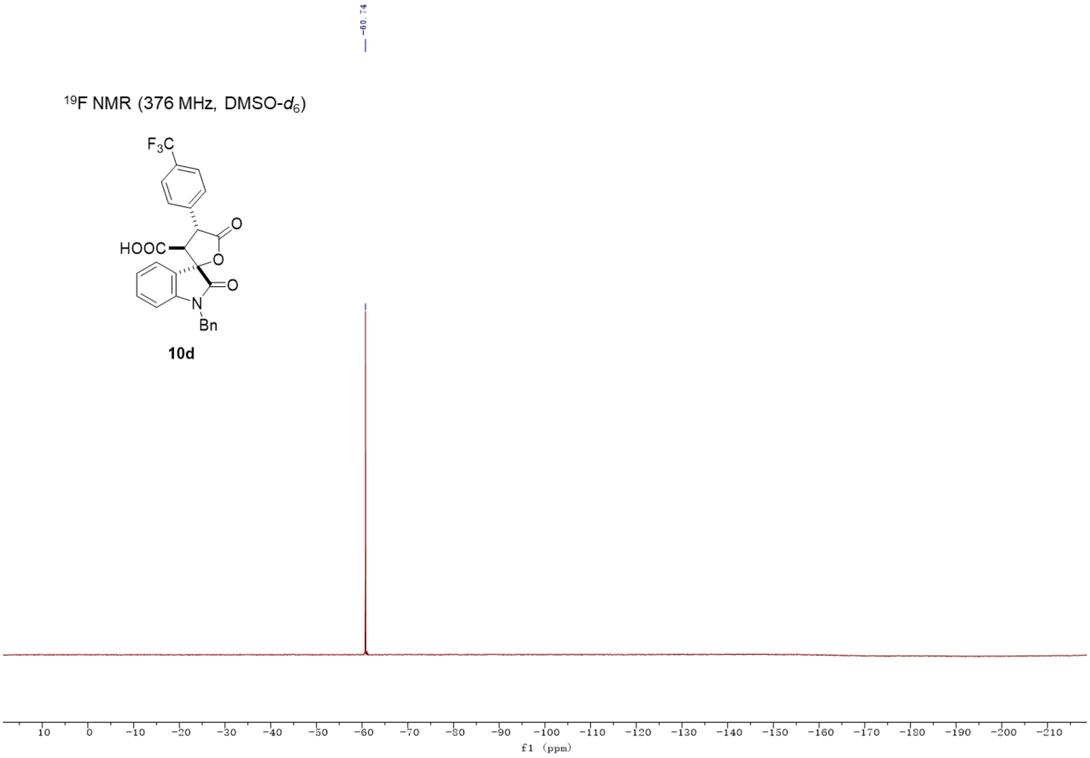
−60.74
^{19}F NMR (376 MHz, DMSO-d_6)
F$_3$C
HOOC
O
N
Bn
10d
f1 (ppm)

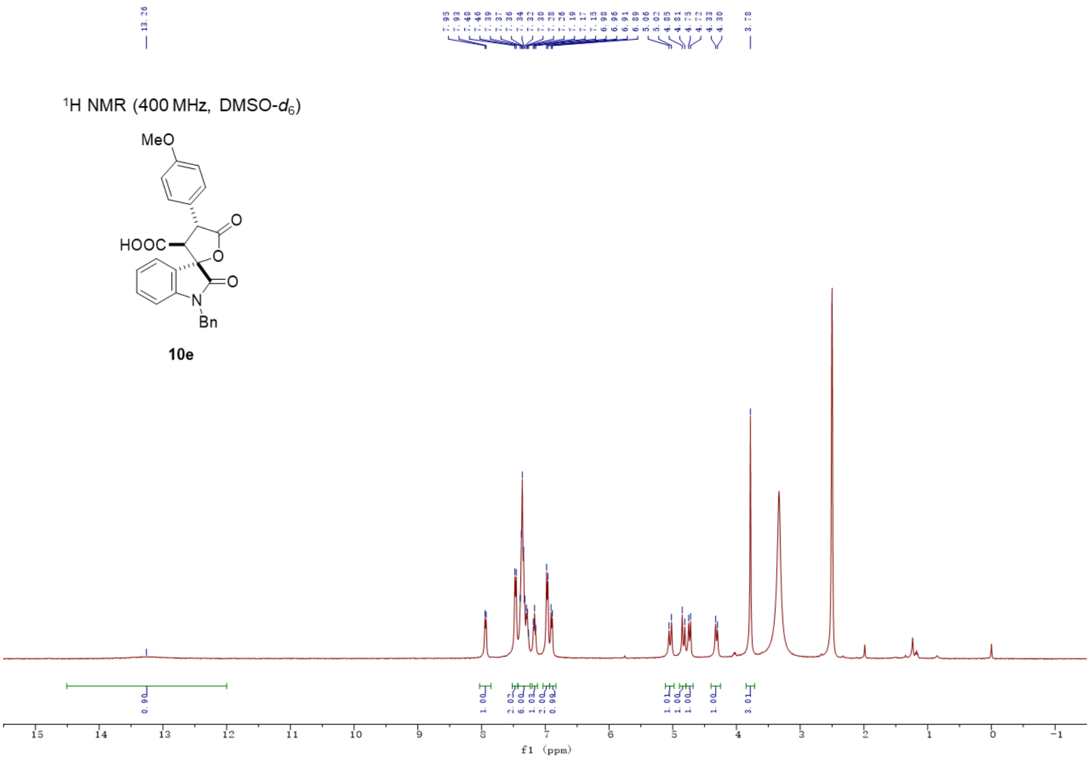
13.26
7.95 7.93 7.48 7.46 7.39 7.37 7.36 7.34 7.32 7.30 7.28 7.26 7.19 7.17 7.15 6.98 6.96 6.91 6.89 5.06 5.02 4.85 4.81 4.75 4.72 4.33 4.30
3.78
1H NMR (400 MHz, DMSO-d6)
MeO
O
HOOC
O
O
N
Bn
10e
0.90
1.00
2.02 6.00 1.03 1.02 2.00 0.99
1.01 1.00 1.00
1.00
3.01
f1 (ppm)

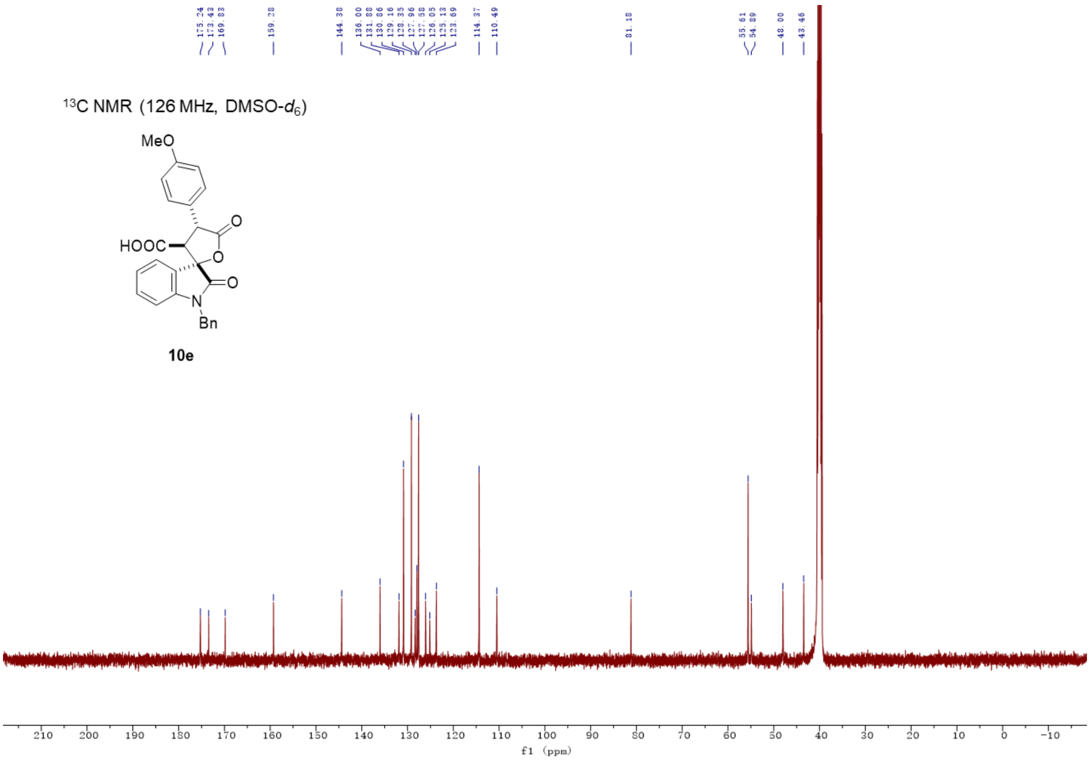
175.24 173.43 169.83
159.28
144.38
136.00 131.88 130.86 129.16 128.35 127.96 127.58 126.05 125.13 123.69
114.37 110.49
81.18
55.61 54.89
48.00
43.46
13C NMR (126 MHz, DMSO-d6)
MeO
O
HOOC
O
O
N
Bn
10e
f1 (ppm)

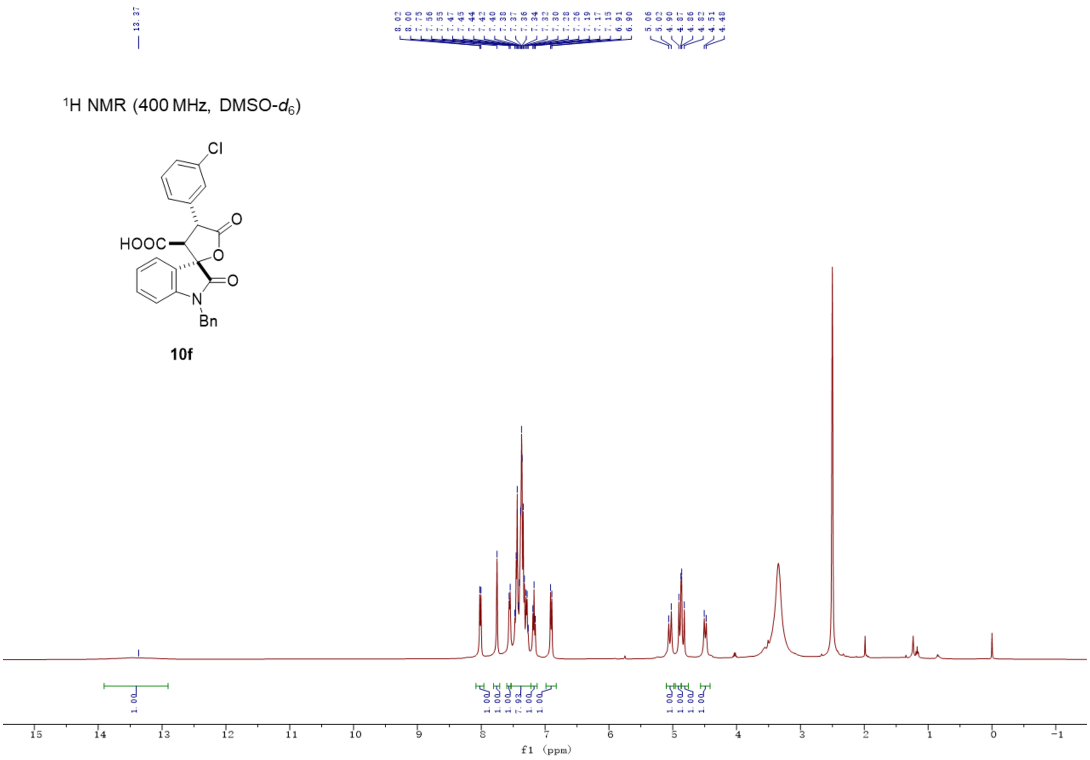
13.37
8.02
8.00
7.75
7.56
7.55
7.47
7.45
7.44
7.42
7.40
7.38
7.37
7.36
7.34
7.32
7.30
7.28
7.26
7.19
7.17
7.15
6.91
6.90
5.06
5.02
4.90
4.87
4.86
4.82
4.51
4.48
^{1}H NMR (400 MHz, DMSO-d_6)
Cl
O
HOOC
O
O
N
Bn
10f
1.00
1.00
1.00
1.00
7.94
1.00
1.00
1.00
1.00
1.00
1.00
f1 (ppm)

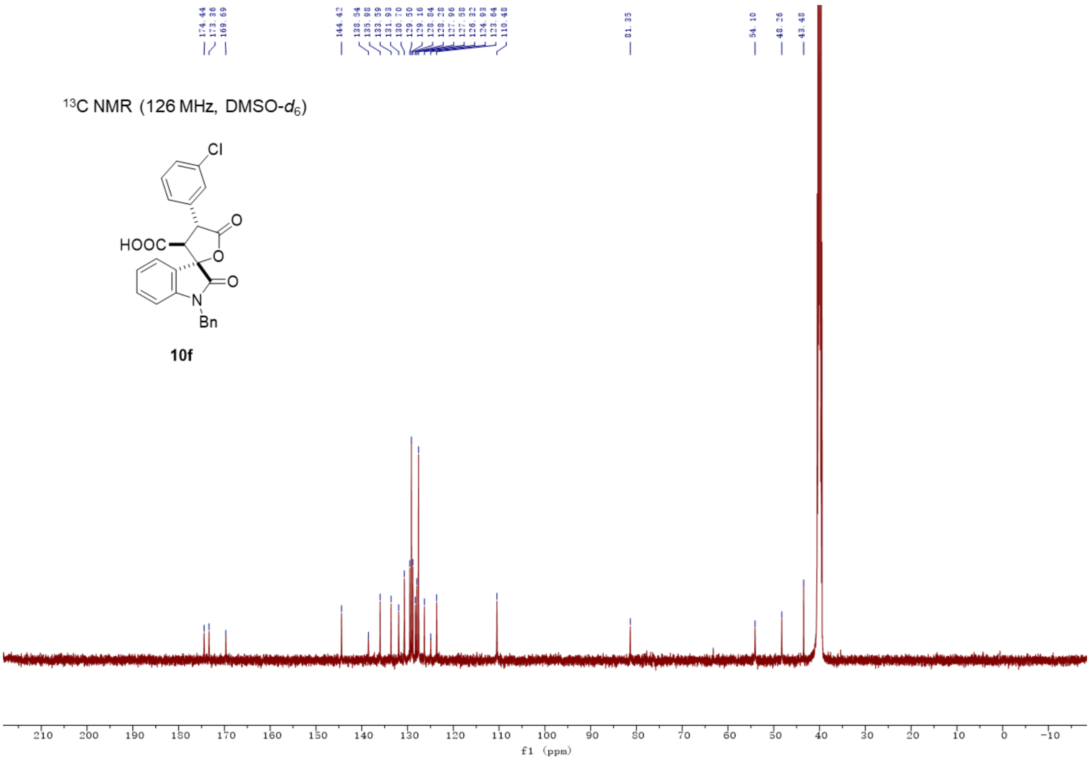
174.44
173.36
169.69
144.42
138.54
135.98
133.59
131.93
130.70
129.50
129.16
128.84
128.28
127.96
127.58
126.32
124.93
123.64
110.48
81.35
54.10
48.26
43.48
^{13}C NMR (126 MHz, DMSO-d_6)
Cl
O
HOOC
O
O
N
Bn
10f
f1 (ppm)

1H NMR (400 MHz, Chloroform-*d*)

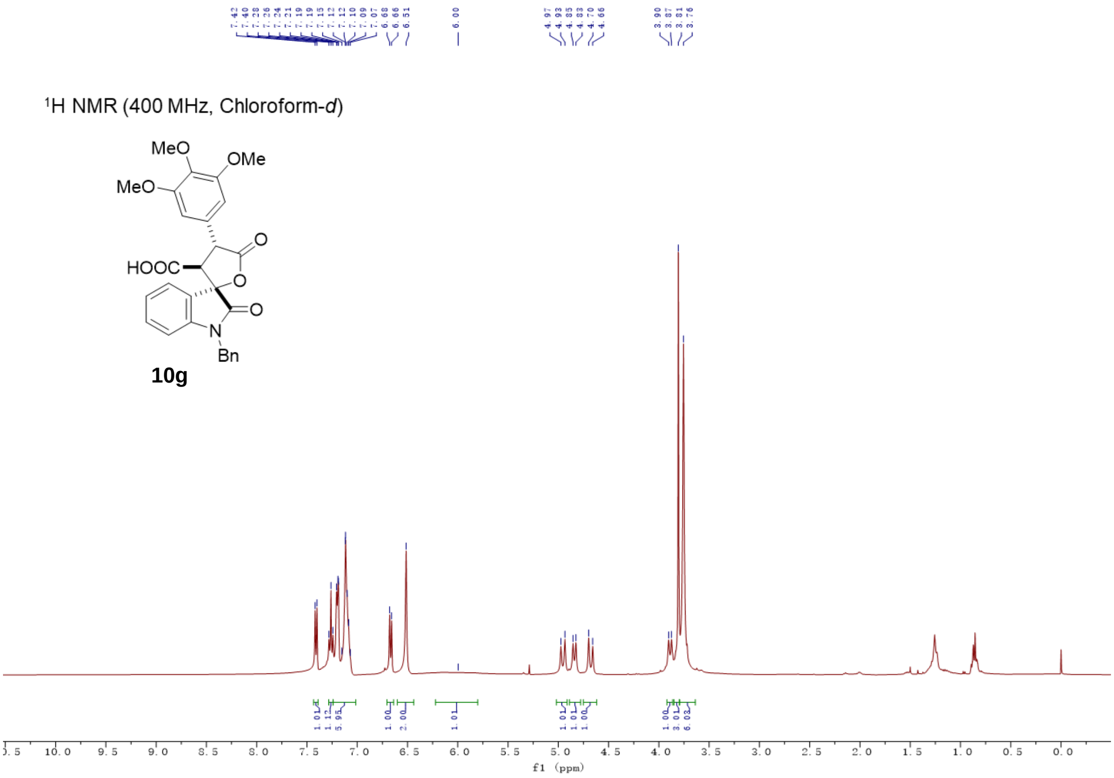

10g

13C NMR (101 MHz, Chloroform-*d*)

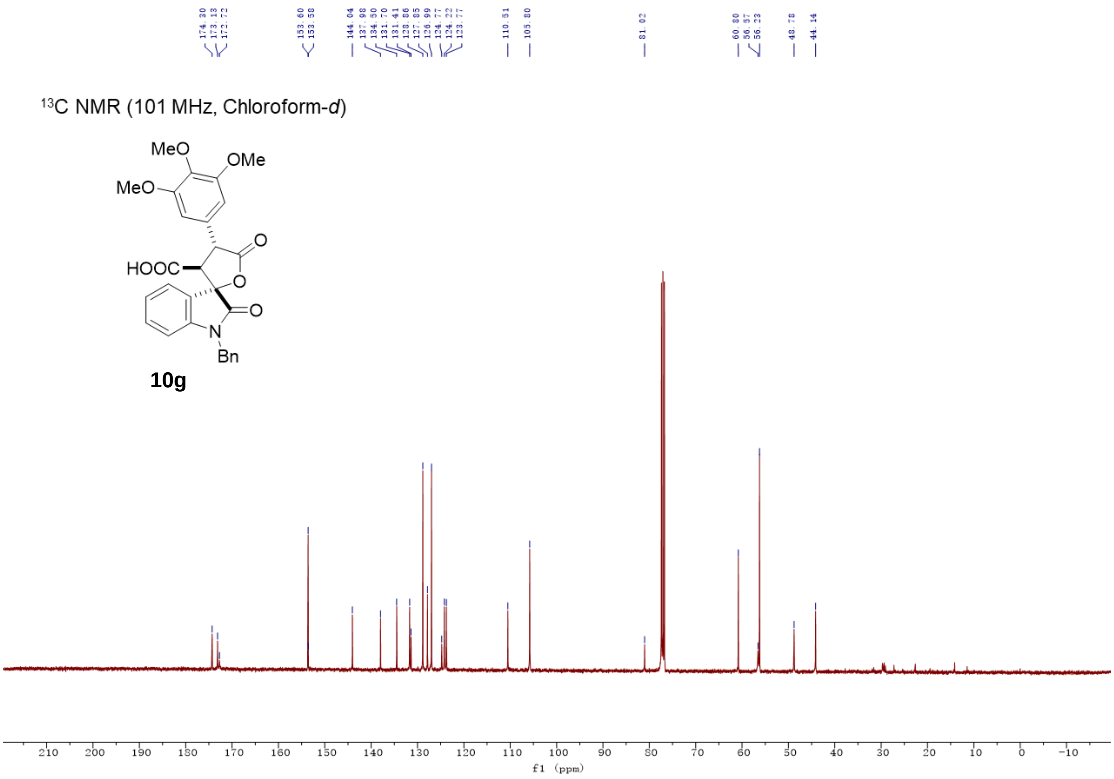

10g

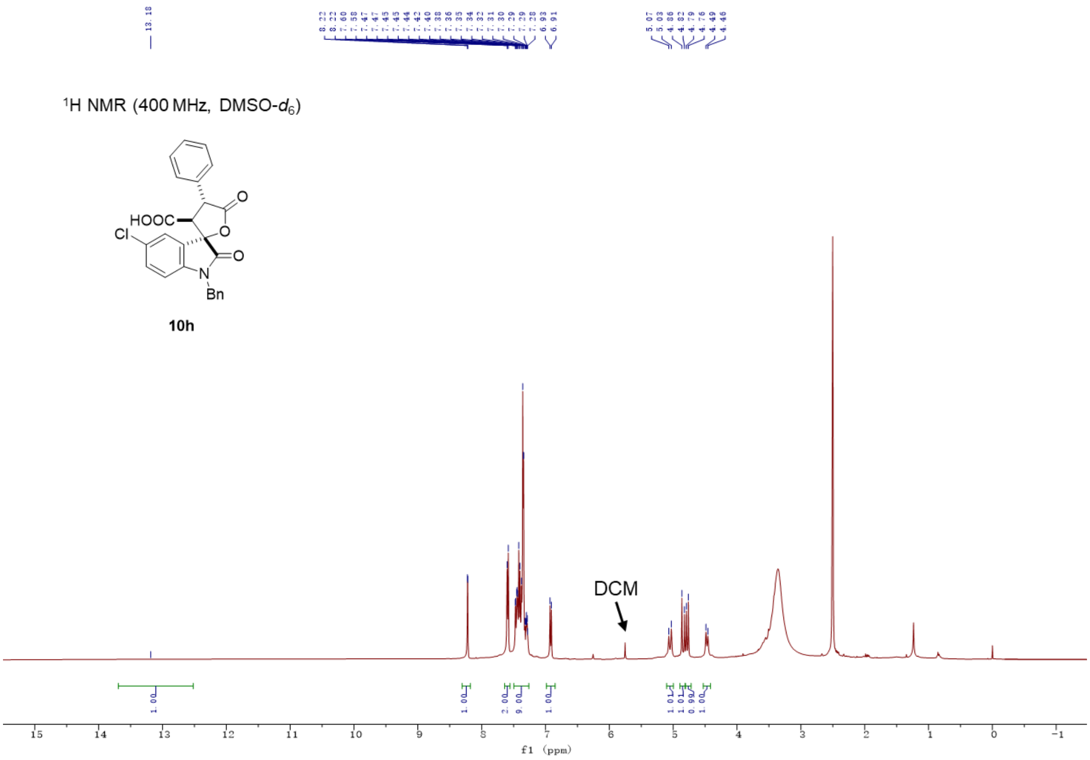

1H NMR (400 MHz, DMSO-d6)
HOOC
Cl
O
N
Bn
10h
DCM
f1 (ppm)

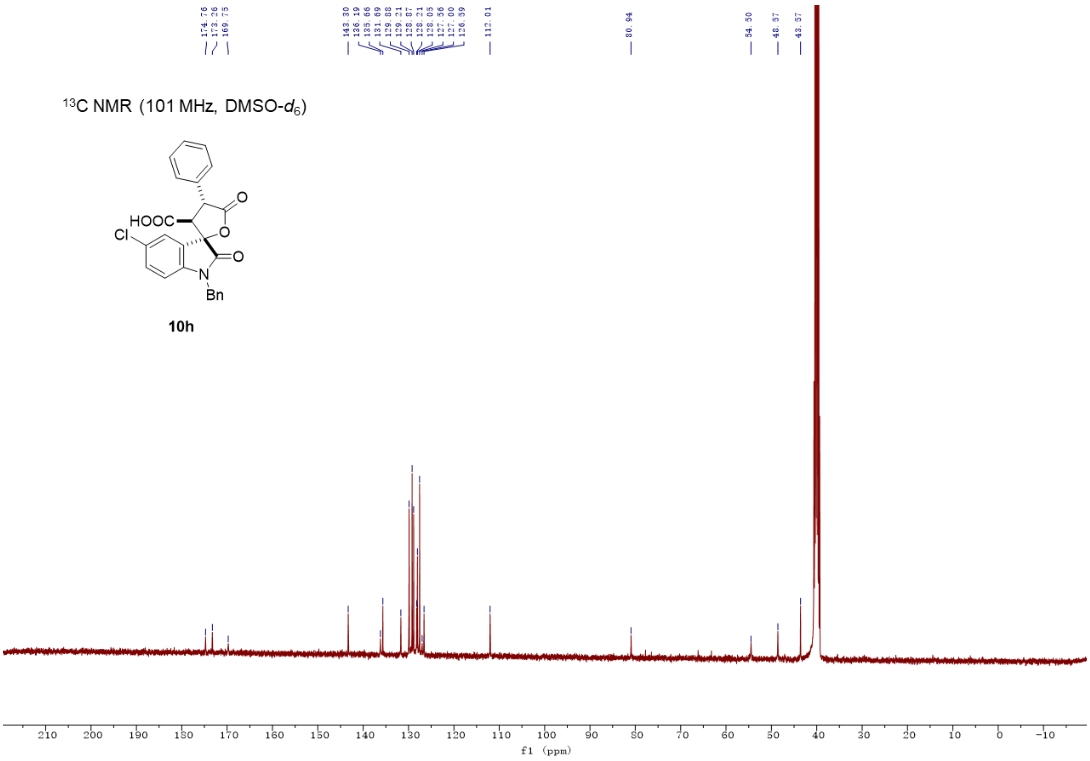

13C NMR (101 MHz, DMSO-d6)
HOOC
Cl
O
N
Bn
10h
f1 (ppm)

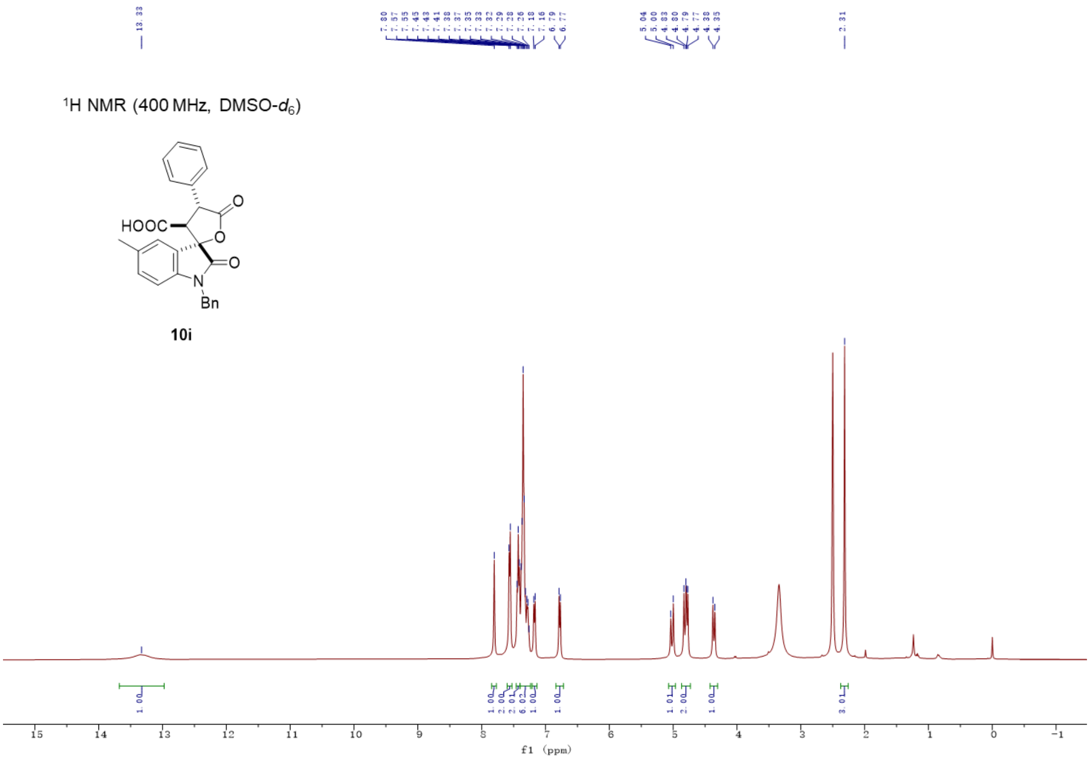

^{1}H NMR (400 MHz, DMSO-d_6)
HOOC
O
O
O
N
Bn
10i
f1 (ppm)

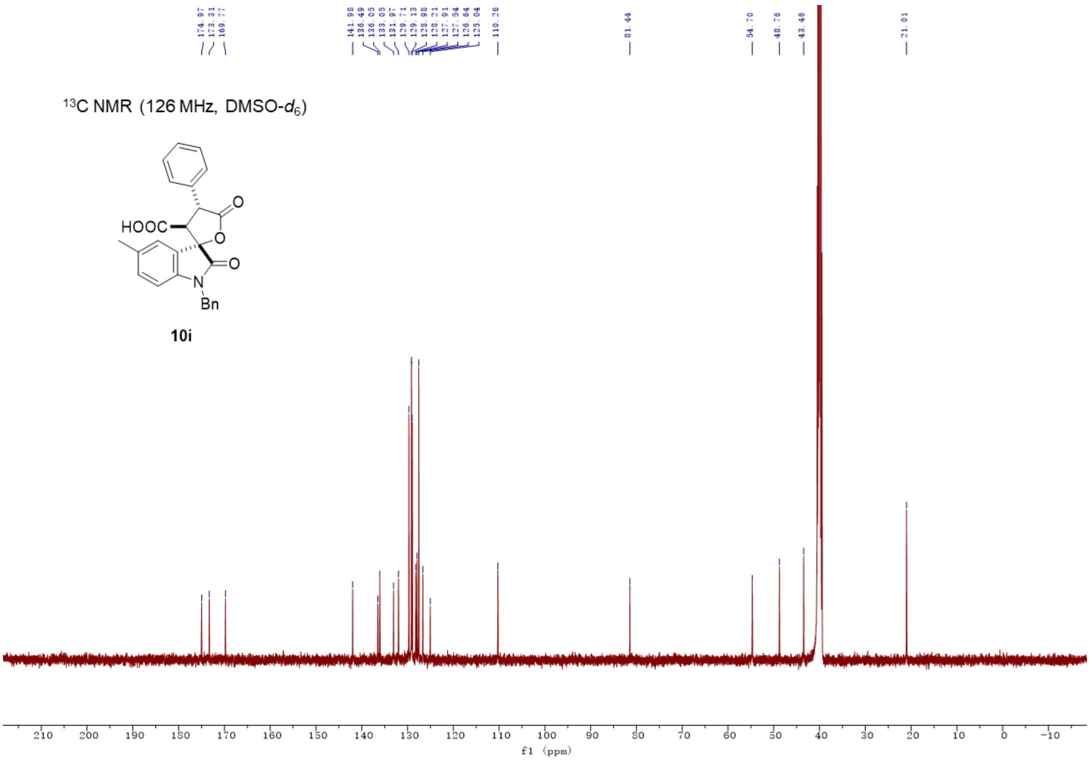

^{13}C NMR (126 MHz, DMSO-d_6)
HOOC
O
O
O
N
Bn
10i
f1 (ppm)

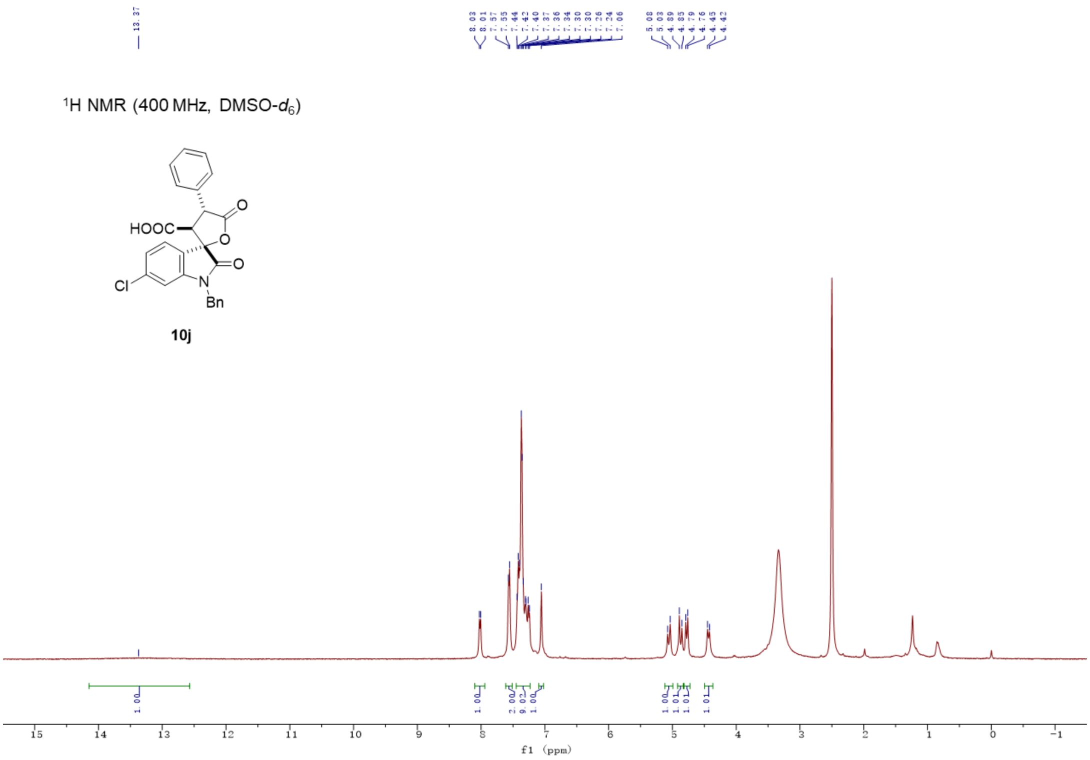

1H NMR (400 MHz, DMSO-d6)
HOOC
O
O
O
Cl
N
Bn
10j
13.37
8.03 8.01 7.57 7.55 7.44 7.42 7.40 7.37 7.36 7.34 7.30 7.30 7.26 7.24 7.06
5.08 5.03 4.89 4.85 4.79 4.76 4.45 4.42
1.00 2.00 9.02 1.00 1.00 1.00 1.01 1.01 1.01 1.01
f1 (ppm)

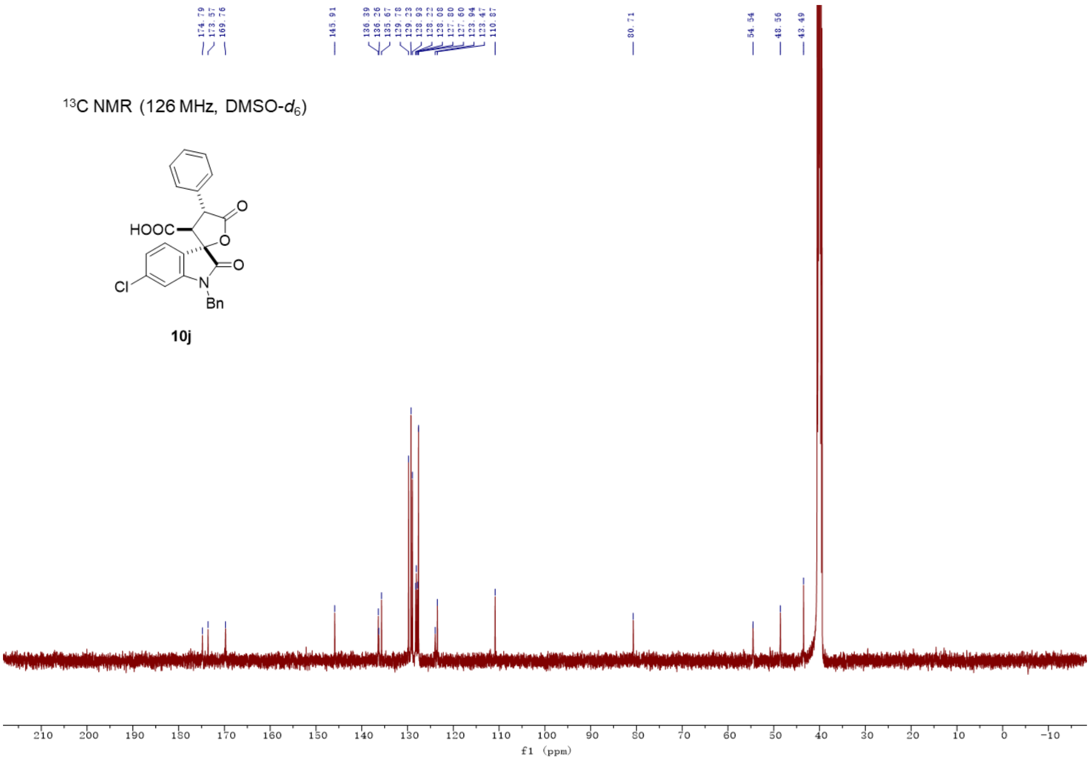

13C NMR (126 MHz, DMSO-d6)
HOOC
O
O
O
Cl
N
Bn
10j
174.79 173.57 169.76
145.91
136.39 136.26 135.67 129.78 129.23 128.93 128.22 128.08 127.80 123.94 123.47 110.87
80.71
54.54 48.56 43.49
f1 (ppm)

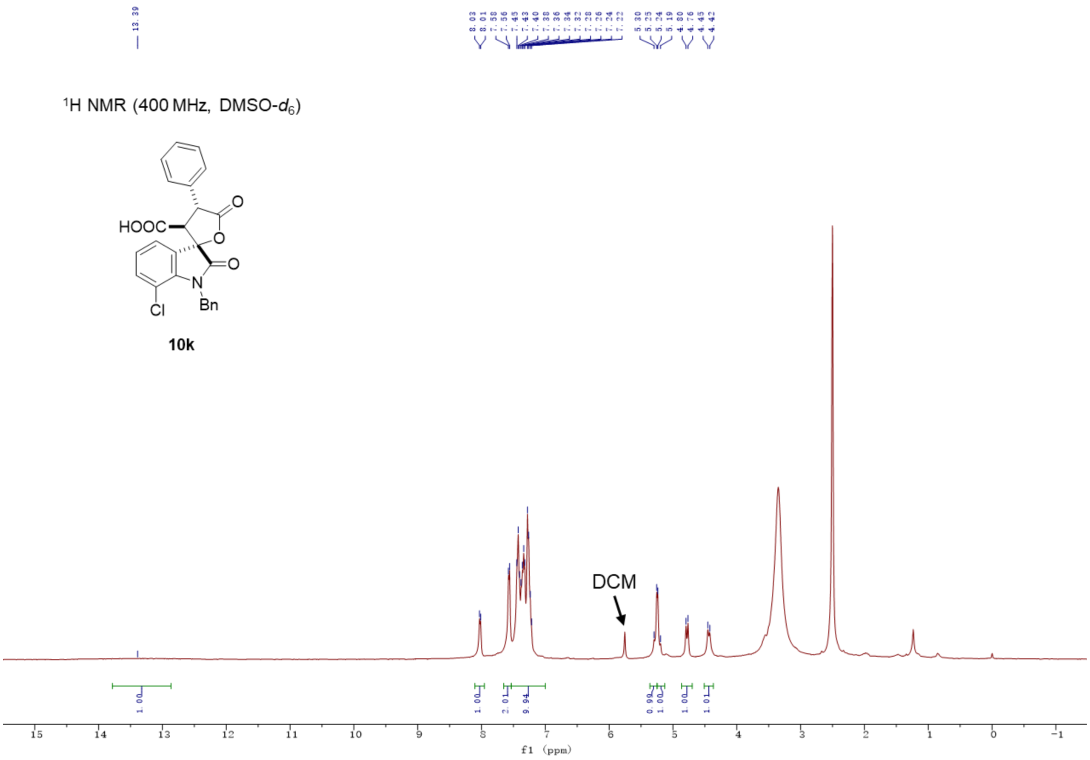
13.39
8.03 8.01 7.58 7.56 7.45 7.43 7.40 7.38 7.36 7.34 7.32 7.28 7.26 7.24 7.22 5.30 5.25 5.24 5.19 4.80 4.76 4.45 4.42
1H NMR (400 MHz, DMSO-d6)
HOOC
O
O
O
N
Cl
Bn
10k
DCM
1.00
1.00 2.01 9.94
0.99 1.00 1.00 1.01
f1 (ppm)

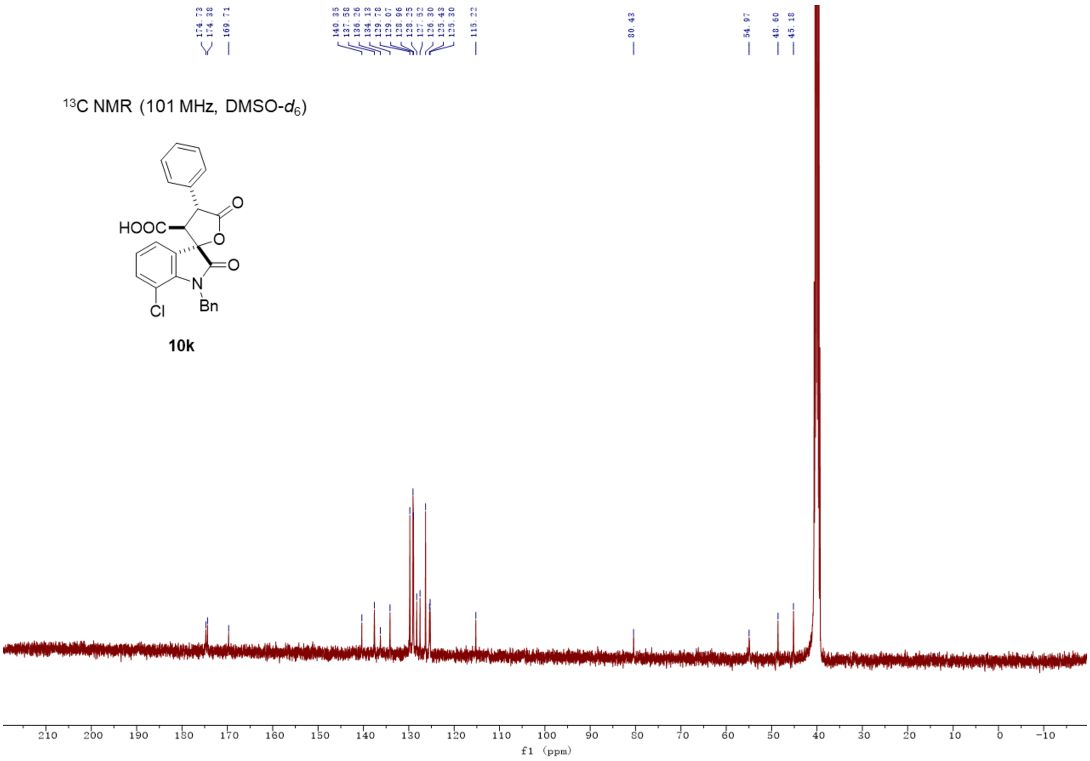
174.73 174.38 169.71
140.35 137.58 136.26 134.13 129.78 129.07 128.96 128.25 127.52 126.30 125.43 125.30 115.22
80.43
54.97 48.60 45.18
13C NMR (101 MHz, DMSO-d6)
HOOC
O
O
O
N
Cl
Bn
10k
f1 (ppm)

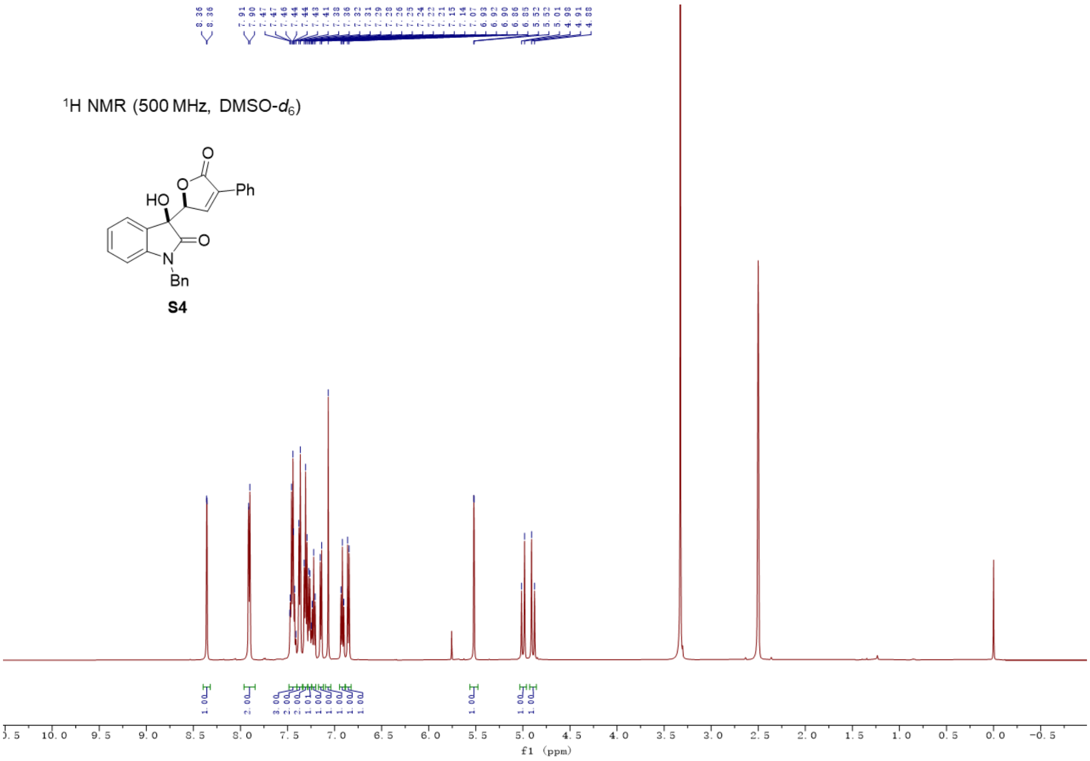
1H NMR (500 MHz, DMSO-d6)
HO
O
Ph
N
Bn
S4
f1 (ppm)

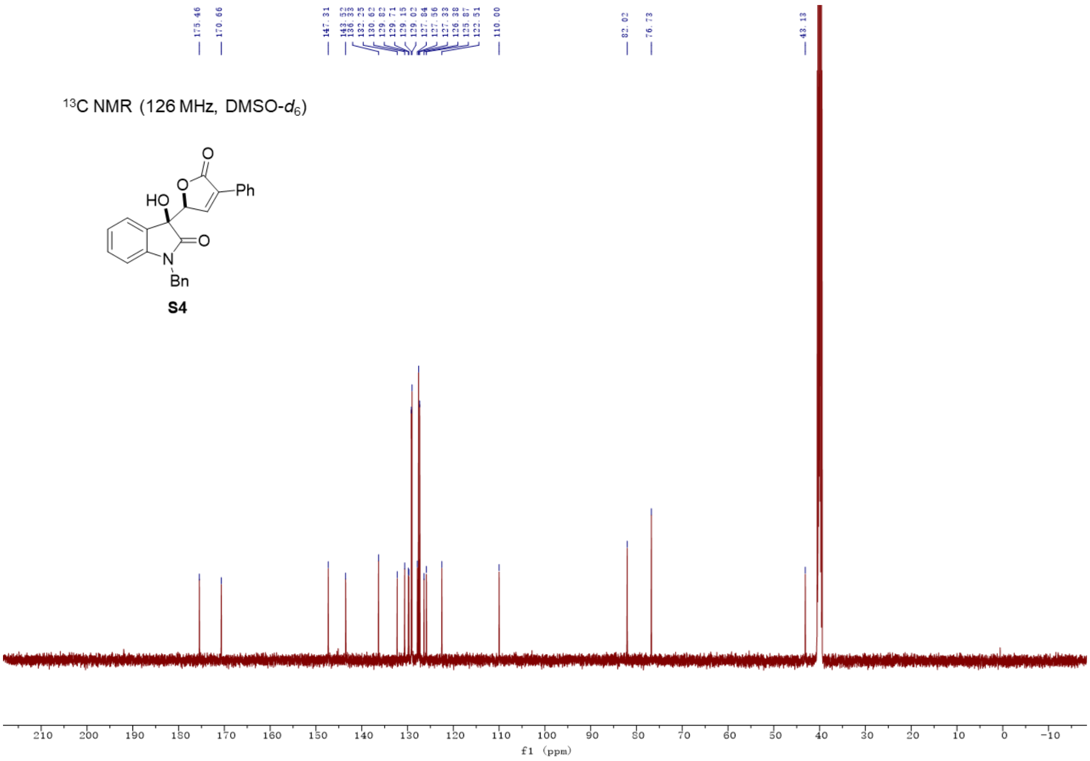
175.46
170.66
147.31
132.25
130.62
129.82
129.71
129.15
129.02
127.84
127.56
127.33
126.38
125.87
122.51
110.00
82.02
76.73
43.13
13C NMR (126 MHz, DMSO-d6)
HO
O
Ph
N
Bn
S4
f1 (ppm)

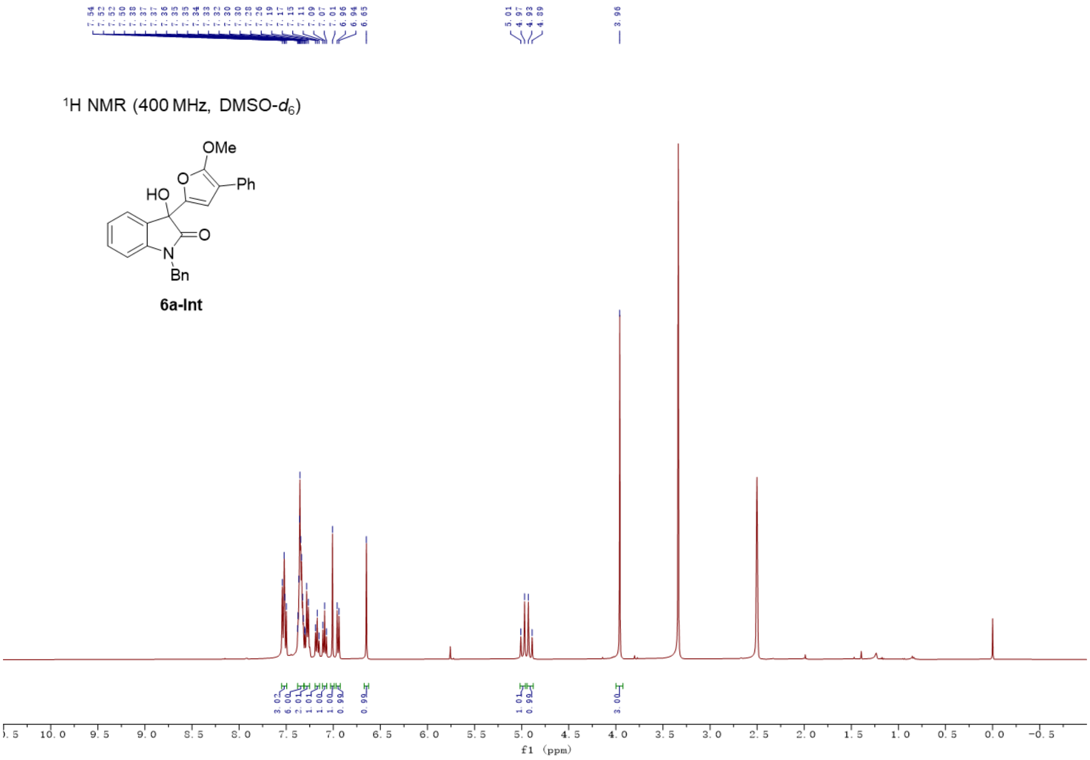
7.54
7.52
7.52
7.38
7.37
7.37
7.36
7.35
7.35
7.34
7.33
7.32
7.30
7.30
7.28
7.26
7.19
7.17
7.15
7.11
7.09
7.07
7.01
6.96
6.94
6.65
5.01
4.97
4.93
4.89
3.98
1H NMR (400 MHz, DMSO-d6)
OMe
O
Ph
HO
O
N
Bn
6a-Int
f1 (ppm)

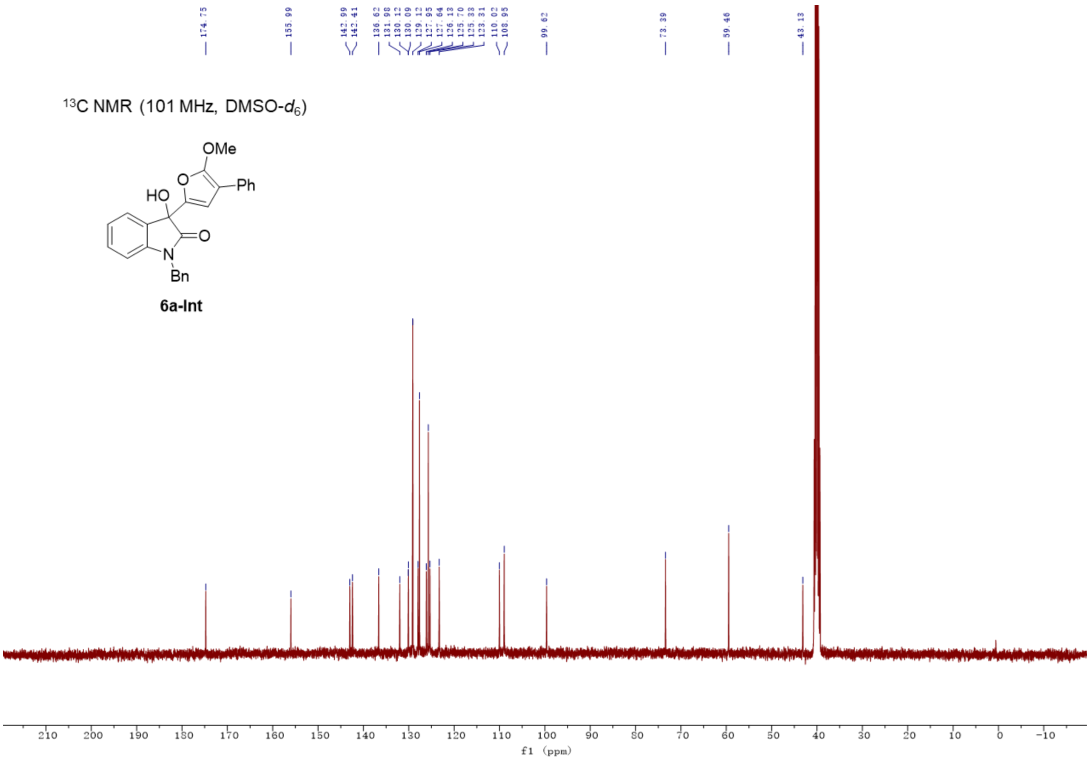
174.75
155.99
142.99
142.41
136.62
131.98
130.12
130.09
129.12
127.95
127.64
126.13
125.70
125.33
123.31
110.02
108.95
99.62
73.39
59.46
43.13
13C NMR (101 MHz, DMSO-d6)
OMe
O
Ph
HO
O
N
Bn
6a-Int
f1 (ppm)

^{1}H NMR (500 MHz, Chloroform-*d*)

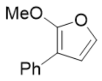

11

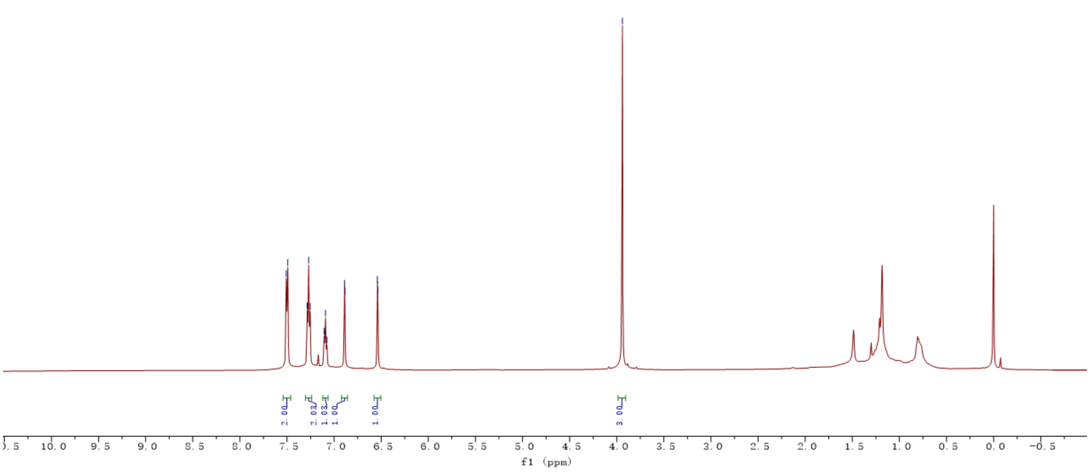

^{13}C NMR (126 MHz, Chloroform-*d*)

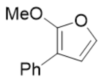

11

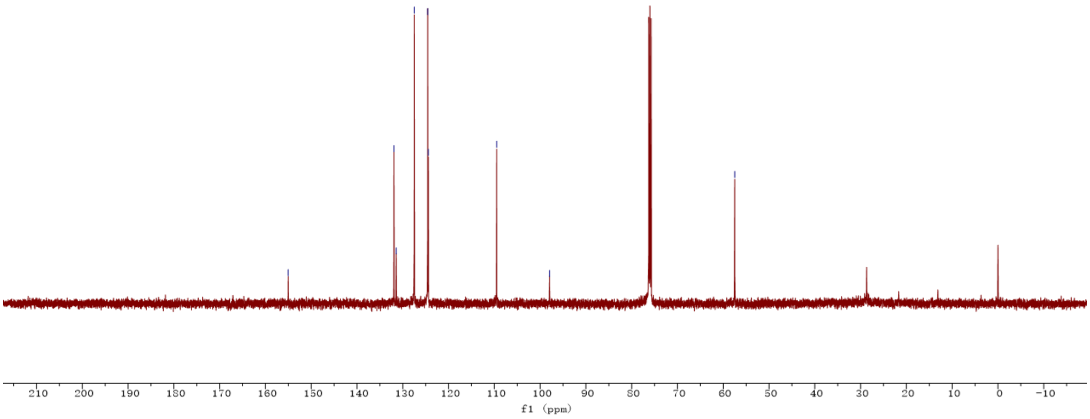

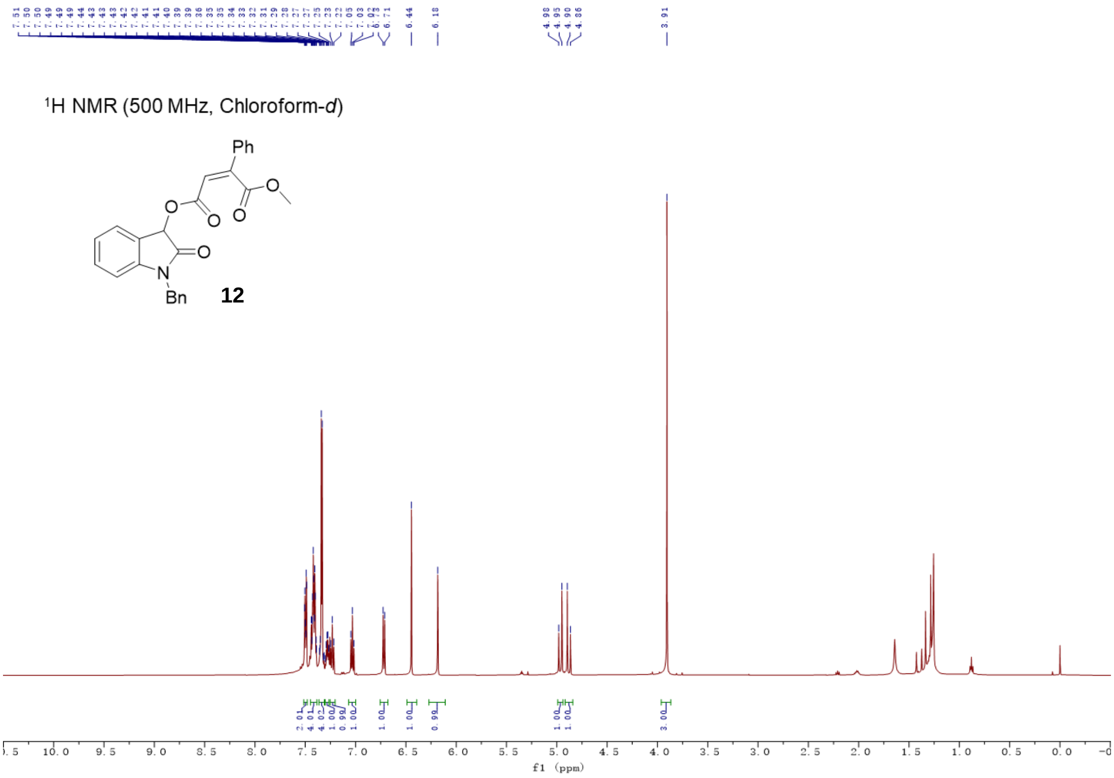
1H NMR (500 MHz, Chloroform-d)
Ph
O
N
Bn
12
6.44
6.18
4.98
4.95
4.90
4.86
3.91
f1 (ppm)

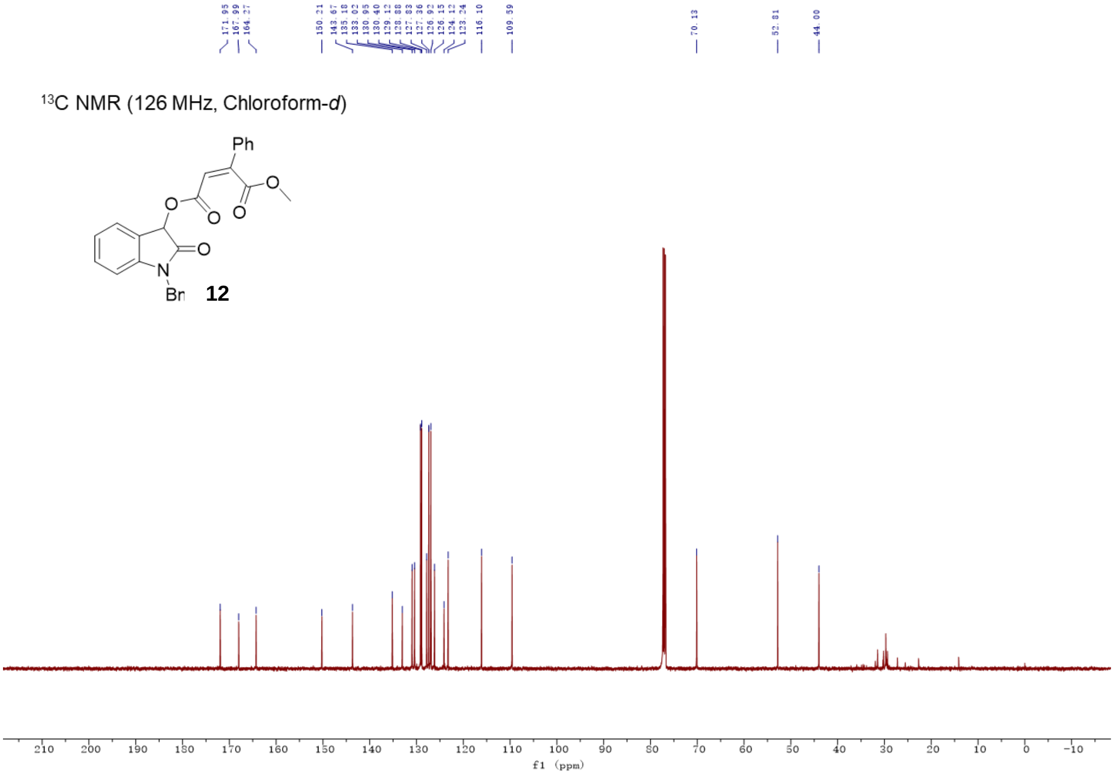
13C NMR (126 MHz, Chloroform-d)
Ph
O
N
Bn
12
171.95
167.99
164.27
150.21
143.67
135.18
133.02
130.95
130.40
129.12
128.88
127.83
127.36
126.92
126.15
124.12
123.24
116.10
109.59
70.13
52.81
44.00
f1 (ppm)

12. Single Crystal X-ray Diffraction Data

Data for 6a

Method for crystal growth: the sample (40 mg) in 25 mL flask was dissolved in 4 mL EtOAc by heating. After complete dissolution, 12 mL hexane was added. The flask was covered by parafilm with several pinhole and placed at bench. The crystal suitable for crystal measurement would grow in the next day.

Ellipsoids are drawn at the 50% probability level.

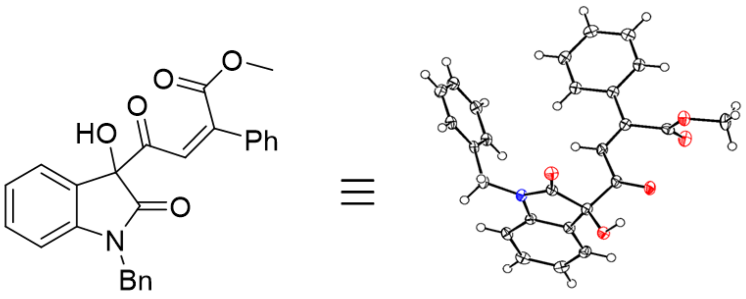

6a **CCDC 2249289**

Bond precision: C-C = 0.0022 A Wavelength=1.54184

Cell:	a=10.0979(2)	b=14.5397(2)	c=27.9690(4)
	alpha=90	beta=94.012(1)	gamma=90
Temperature:	100 K		

	Calculated	Reported
Volume	4096.36(11)	4096.36(11)
Space group	P 21/c	P 1 21/c 1
Hall group	-P 2ybc	-P 2ybc
Moiety formula	C26 H21 N O5	2(C26 H21 N O5)
Sum formula	C26 H21 N O5	C52 H42 N2 O10
Mr	427.44	854.87
Dx,g cm-3	1.386	1.386
Z	8	4
Mu (mm-1)	0.790	0.790
F000	1792.0	1792.0
F000'	1797.74	
h,k,lmax	12,18,35	12,18,35
Nref	8501	8075
Tmin,Tmax		0.319,1.000
Tmin'		

Correction method= # Reported T Limits: Tmin=0.319 Tmax=1.000
AbsCorr = MULTI-SCAN

Data completeness= 0.950 Theta(max)= 75.497

R(reflections)= 0.0614(6912) wR2(reflections)= 0.1734(8075)

S = 1.047 Npar= 581

Data for 10a

Method for crystal growth: the sample (15 mg) in 5 mL flask was dissolved in 2 mL EtOAc by heating. After complete dissolution, 1 mL hexane was added. The flask was covered by parafilm with several pinhole and placed at bench. The crystal suitable for crystal measurement would grow in the next day.

Ellipsoids are drawn at the 50% probability level.

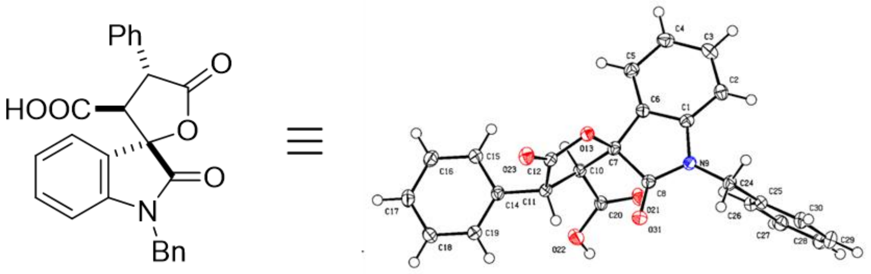

10a **CCDC 2254233**

Bond precision:	C-C = 0.0019 A		Wavelength=1.54184
Cell:	a=13.0226(2)	b=15.7217(2)	c=10.5082(1)
	alpha=90	beta=110.522(1)	gamma=90
Temperature:	100 K		

	Calculated	Reported
Volume	2014.89(5)	2014.89(5)
Space group	P 21/c	P 1 21/c 1
Hall group	-P 2ybc	-P 2ybc
Moiety formula	C25 H19 N O5	C25 H19 N O5
Sum formula	C25 H19 N O5	C25 H19 N O5
Mr	413.41	413.41
Dx,g cm-3	1.363	1.363
Z	4	4
Mu (mm-1)	0.785	0.785
F000	864.0	864.0
F000'	866.80	
h,k,lmax	16,19,13	16,19,13
Nref	4169	3979
Tmin,Tmax	0.828,0.925	0.840,1.000
Tmin'	0.822	

Correction method= # Reported T Limits: Tmin=0.840 Tmax=1.000
AbsCorr = MULTI-SCAN

Data completeness= 0.954 Theta(max)= 75.385

R(reflections)= 0.0345(3693) wR2(reflections)= 0.0855(3979)

S = 1.032 Npar= 282

13. Computational Studies

Method

We performed calculations with Gaussian 09 program[2], We ran the geometry optimization, frequency and TDDFT calculations of structures with m062x[3] or PBE1PBE[4]/6-31G(d)/Lanl2dz (6-31G(d) is applied to nonmetal elements such as C, H, O, N and Lanl2dz is used for rhodium element).[2] We carried out intrinsic reaction coordinate calculations to ensure TS is connect with starting materials and products. We calculated single point energy at m062x/6-311++G(2d,2p)/Lanl2dz. We added Solvent effect with self-consistent reaction field (SCRF) method based on SMD model in ethylethanoate (ε=8.86) or in tetrahydrofuran (ε=7.43) . The energenetics results were processed via Gaussian View 6. The 3D optimized structures were displayed by CYLview visualization program.

Calculated Enthalpy and Gibbs Free Energy

Structures	*H* (Hartree)	*G* (Hartree)	*ΔΔH* (Kcal/mol)	Sigle point energy (Hartree)	Correction to G (Hartree)	*ΔΔG* (Kcal/mol)
**$Rh_2(OAc)_4$**	-1132.266227	-1132.339919	/	-1132.822681	0.162087	/
**$Rh_2(OAc)_4$-EtOAc**	-1747.183599	-1747.297973	/	-1747.681061	0.383087	/
**4**	-575.134377	-575.185115	0	-575.513052	0.145012	0
**Int1**	-1707.392877	-1707.496313	20.2872385	-1708.333942	0.330906	67.207549
**Int2**	-1707.463064	-1707.56284	-163.98873	-1708.402573	0.336761	-97.610839
**TS1**	-1707.398891	-1707.498285	4.4974815	-1708.333906	0.331501	68.8642395
**Int3**	-575.176777	-575.225715	-111.3212	-575.55518	0.149502	-98.818569
**5**	-782.890399	-782.94698	/	-783.373266	0.186472	/
**HOAc**	-228.92248	-228.954898	/	-229.081608	0.035805	/
**TS2**	-1586.988044	-1587.083747	-107.088894	-1588.006726	0.414612	22.3771365
**TS3**	-1358.053332	-1358.134888	-74.973778	-1358.910657	0.360568	12.4579975
**7**	-1358.083753	-1358.171236	-43.5229135	-1358.950389	0.356841	-2.825038
**O2**	-150.198233	-150.220454	/	-150.261407	-0.014846	/
**Int4**	-1508.364757	-1508.4509	-372.159374	-1509.292135	0.367644	-245.232202
**Int5**	-1737.319276	-1737.416719	-456.2777685	-1738.400417	0.426326	-255.2012255
**HOOAc**	-304.017001	-304.052055	/	-304.207021	0.037553	/
**Z-6**	-1433.296752	-1433.3866	-441.777132	-1434.200289	0.35931	350.6539035
**E-6**	-1433.294584	-1433.381668		-1434.196205	0.362297	-332.088993
**TS4**	-1447.833617	-1447.924102	-			
**TS5**	-1523.090334	-1523.178004				

14. Molecular docking

This was done using AutoDock Vina software[5]. Ligands were created with ChemOffice 2014 Chem3D software. Energy-minimized conformations of **6** were output with .pdb format. Then the ligand torsion parameters were set in AutoDockTools to give.pdbqt format. The coordinates for PTP1B (PDB ID: 1T4J, https://www.rcsb.org/structure/1T4J) were downloaded from the Protein Data Bank. The original binding ligand was deleted from the .pdb file, and hydrogen atoms were added. Then flexible and rigid PTP1B files were created in AutoDockTools. The binding model was analyzed in MacPyMOL 2.0. and presented in Supplementary Fig.

15

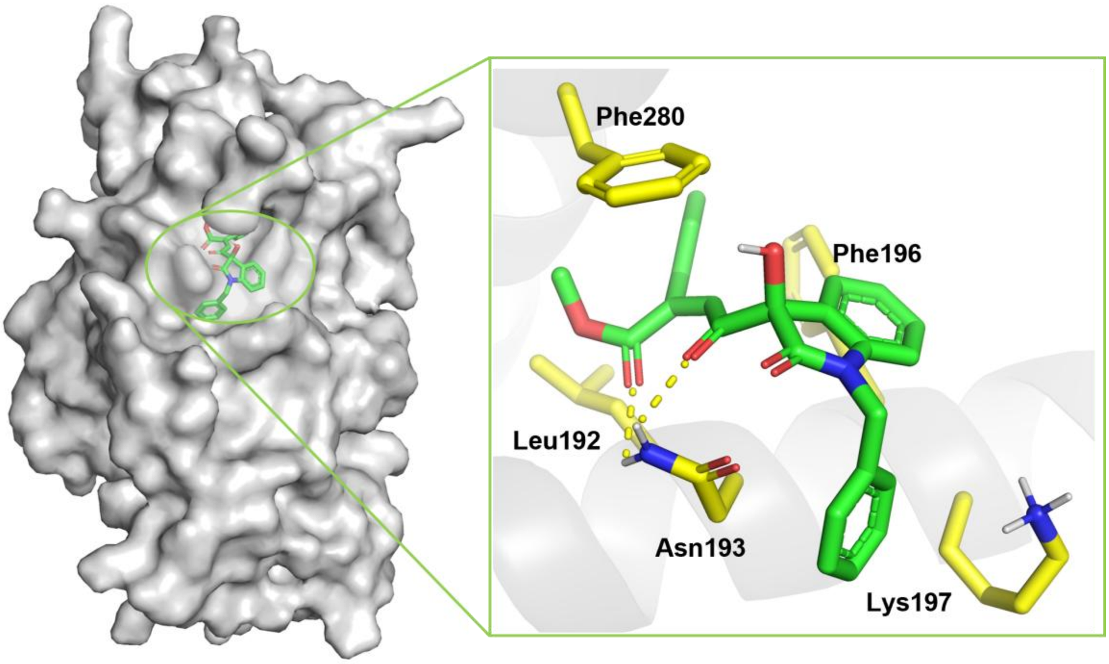

Supplementary Figure 15. Binding mode of compound 6a with an allosteric site in PTP1B (PDB ID: 1T4J, https://www.rcsb.org/structure/1T4J) predicted by molecular docking. The H-bond interactions are indicated with yellow dashed lines.

15. Biological activity evaluation

We screened compounds **6** against PTP1B and TCPTP via 6,8-Difluoro-4-Methylumbelliferyl Phosphate (DiFMUP) assay. Preliminary experiment found that **6** compounds including **6a**, **6g**, **6i**, **6m**, **6n**, and **6r** inhibit PTP1B with 4.63±0.42μM, 0.28±0.04μM, 4.56±0.24 μM, 7.21±0.52 μM, 5.87±0.53 μM and 3.75±0.39 μM IC_{50}, and 2.76, 8.14, 1.26, 0.51, 6.84, and 6.07-fold selectivity against its isoform TCPTP (Supplementary Fig. 16). The results proved compounds **6g**, **6n** and

6r to be promising hits of PTP1B inhibitors.

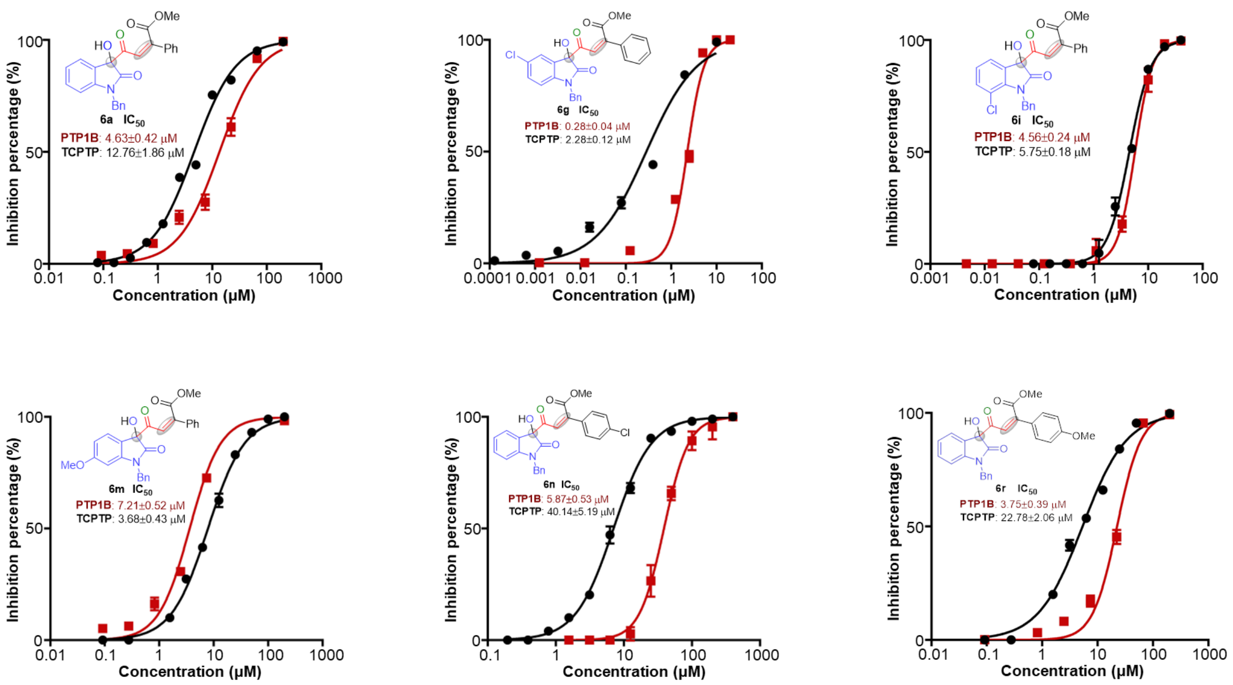

Supplementary Figure 16. Identification of inhibitory activity against PTP1B and TCPTP.

Protein expression and purification: Human PTP1B construct and human TCPTP construct were subcloned into pET28a(+) vector, which contains an N-terminal His6-tag. BL21 (DE3) *E.coli* Cells were transformed as follows: 2 µL of plasmid (100 ng/µL) and 200 µL of DE3 cells were incubated for 30 min on ice, a 45-second heat shock at 37 °C, 2 min on ice, recovery with 800 µL SOC for 1 h at 37 °C followed by plating on a Kanamycin agar plate (50 µg/mL). One colony was picked and used to inoculate 10 mL of LB media supplemented with kanamycin (50 µg/mL). When the culture reached OD_{600} = 0.6, 10 mL were transferred to one liter of LB media supplemented with Kanamycin (50 µg/mL) and grown at 37 °C to an OD_{600} = 0.6. Media is transferred to a 16 °C incubator. Then, the media was supplemented with 500 µM Isopropyl *β*-D-1-thiogalactopyranoside (IPTG) at OD_{600} = 0.8 and incubated for 16 h. Cells were centrifuged (3000 x g, 4 °C, 20 min) and liquid was decanted. Cells were resuspended in lysis buffer (50 mM HEPES, pH 7.4, 150 mM KCl, 5 mM $MgCl_2$, 5% glycerol, 1 mM phenylmethane sulfonyl fluoride (PMSF), 2 mM 2-mercaptoethanol (β-met)). Cells were lysed in a cell crusher (pressure 1000 bar), the mixed products were collected, centrifuged (16,000 x g, 4 °C, 20 min), and the

supernatant was left to discard the precipitate. The nickel column was first equilibrated with equilibration solution, and then the protein supernatant was up-sampled twice to bind the protein. The proteins were then gradually eluted with imidazole (10, 20, 60, 200 mM imidazole). The target protein-containing fractions confirmed by SDS PAGE were placed in 30,000 kDa MWCO protein ultrafiltration tubes and the imidazole was replaced with dialysis buffer (20 mM HEPES, 150 mM KCl, 1 mM $MgCl_2$). After the imidazole was removed cleanly, an equal volume of glycerol was added as a protective solution. When concentration was determined to be greater than or equal to 1 mg /mL, protein was frozen with liquid nitrogen. Protein was then stored in a -80 °C freezer.

In Vitro Phosphatase Activity Assays[6]： Incubate 1 nM purified PTP1B or TCPTP protein with positive control or compound to be tested in reaction buffer (25 mM Bis-Tris propane, pH 7.5, 50 mM NaCl, 2 mM EDTA, 2 mM DTT) for 10 min at 37 °C in a total volume of 90 µL. Add 10 µL of substrate buffer containing DiFMUP (final concentration of 10 µM) to the reaction buffer and incubate for 10 min at 37 °C. The fluorescence intensity at 455 nm was read using a multifunctional reader with 358 nm as the excitation light, and the measured fluorescence value was used to calculate the inhibition rate of the enzyme activity by the sample over the value of the blank wells. The IC_{50} values of the compounds were calculated by Graph pad Prism 7.0, using a non-linear fit of the inhibition rate to the concentration of the inhibitor.

16. References

1. Zhao, Z. Y.; Nie, Y. X.; Tang, R. H.; Yin, G. W.; Cao, J.; Xu, Z.; Cui, Y. M.; Zheng, Z. J.; Xu, L. W. *ACS. Catal*. ***9***, 9110 (2019).

2. Gaussian 09, Revision D.01,

M. J. Frisch, G. W. Trucks, H. B. Schlegel, G. E. Scuseria, M. A. Robb, J. R. Cheeseman, G. Scalmani, V. Barone, B. Mennucci, G. A. Petersson, H. Nakatsuji, M.

Caricato, X. Li, H. P. Hratchian, A. F. Izmaylov, J. Bloino, G. Zheng, J. L. Sonnenberg, M. Hada, M. Ehara, K. Toyota, R. Fukuda, J. Hasegawa, M. Ishida, T. Nakajima, Y. Honda, O. Kitao, H. Nakai, T. Vreven, J. A. Montgomery, Jr., J. E. Peralta, F. Ogliaro, M. Bearpark, J. J. Heyd, E. Brothers, K. N. Kudin, V. N. Staroverov, T. Keith, R. Kobayashi, J. Normand, K. Raghavachari, A. Rendell, J. C. Burant, S. S. Iyengar, J. Tomasi, M. Cossi, N. Rega, J. M. Millam, M. Klene, J. E. Knox, J. B. Cross, V. Bakken, C. Adamo, J. Jaramillo, R. Gomperts, R. E. Stratmann, O. Yazyev, A. J. Austin, R. Cammi, C. Pomelli, J. W. Ochterski, R. L. Martin, K. Morokuma, V. G. Zakrzewski, G. A. Voth, P. Salvador, J. J. Dannenberg, S. Dapprich, A. D. Daniels, O. Farkas, J. B. Foresman, J. V. Ortiz, J. Cioslowski, and D. J. Fox, Gaussian, Inc., Wallingford CT, 2013;

3. E. G. Hohenstein, S. T. Chill, and C. D. Sherrill, *J. Chem. Theory Comput.* **4**, 1996–2000 (2008);

4. Ö. Tamer, F. Gözüaçık, D. Avcı, Y. Atalay *Opt. Spectrosc.*, **116**, 12–32 (2014)

5. G. M. Morris, R. Huey, W. Lindstrom, M. F. Sanner, and A. J. Olson, *J. Comput. Chem*, **16**, 2785-2791 (2009).

6. Kyriakou, E.; Schmidt, S.; Dodd, G. T.; Pfuhlmann, K.; Simonds, S. E.; Lenhart, D.; Geerlof, A.; Schriever, S. C.; De Angelis, M.; Schramm, K.; Plettenburg, O.; Cowley, M. A.; Tiganis, T.; Tschöp, M. H.; Pfluger, P. T.; Sattler, M.; Messias, A. C., *J. Med. Chem*, **61**, 11144-11157 (2018).
