# Supplementary material for: Bifunctionality of dirhodium tetracarboxylates in metallaphotocatalysis
Source: Nat Commun. 2023 Nov 10;14:7269. doi: 10.1038/s41467-023-43050-3 (PMC10638314; doi:10.1038/s41467-023-43050-3)
Supplement: Supplementary file 3 — Reporting Summary [file 41467_2023_43050_MOESM3_ESM.pdf]

nature portfolio

Corresponding author(s): Taoda Shi and Wenhao Hu

Last updated by author(s): Taoda Shi and Tianyuan Zhang

Reporting Summary

Nature Portfolio wishes to improve the reproducibility of the work that we publish. This form provides structure for consistency and transparency in reporting. For further information on Nature Portfolio policies, see our Editorial Policies and the Editorial Policy Checklist.

Statistics

For all statistical analyses, confirm that the following items are present in the figure legend, table legend, main text, or Methods section.

n/a	Confirmed	
☐	☒	The exact sample size (*n*) for each experimental group/condition, given as a discrete number and unit of measurement
☐	☒	A statement on whether measurements were taken from distinct samples or whether the same sample was measured repeatedly
☒	☐	The statistical test(s) used AND whether they are one- or two-sided<br>*Only common tests should be described solely by name; describe more complex techniques in the Methods section.*
☐	☒	A description of all covariates tested
☐	☒	A description of any assumptions or corrections, such as tests of normality and adjustment for multiple comparisons
☐	☒	A full description of the statistical parameters including central tendency (e.g. means) or other basic estimates (e.g. regression coefficient) AND variation (e.g. standard deviation) or associated estimates of uncertainty (e.g. confidence intervals)
☒	☐	For null hypothesis testing, the test statistic (e.g. *F*, *t*, *r*) with confidence intervals, effect sizes, degrees of freedom and *P* value noted<br>*Give P values as exact values whenever suitable.*
☒	☐	For Bayesian analysis, information on the choice of priors and Markov chain Monte Carlo settings
☒	☐	For hierarchical and complex designs, identification of the appropriate level for tests and full reporting of outcomes
☒	☐	Estimates of effect sizes (e.g. Cohen's *d*, Pearson's *r*), indicating how they were calculated

Our web collection on statistics for biologists contains articles on many of the points above.

Software and code

Policy information about availability of computer code

Data collection	The accessible invirual library was created by open-soure tool RDkit, the code is avaliable at website: https://rdkit.org/
Data analysis	1. The NMR data recorded on Brucker Avance 400 MHz or Ascend 500 MHz spectrometers, and were processed by MestRenova 10.0.0<br>2.The crystallography data was analyzed by Bruker-AXS SMART APEX II single crystal X-ray diffractometer.<br>3. The computational study was conducted by Gaussian 09 software, and the results were analyzed by Gaussian View 6 and CYLview20 software.<br>4. The DiFMUP assay data was analyzed by GraphPad Prism 7.0<br>5. Molecular docking was peformed by Autodock 4.2 and the results were analyzed by PyMol 2.0

For manuscripts utilizing custom algorithms or software that are central to the research but not yet described in published literature, software must be made available to editors and reviewers. We strongly encourage code deposition in a community repository (e.g. GitHub). See the Nature Portfolio guidelines for submitting code & software for further information.

Data

Policy information about availability of data

All manuscripts must include a data availability statement. This statement should provide the following information, where applicable:

- Accession codes, unique identifiers, or web links for publicly available datasets
- A description of any restrictions on data availability
- For clinical datasets or third party data, please ensure that the statement adheres to our policy

1. The invirtual library of the synthetic methodology is avaliable at http://www.sysu-sps-compound.com.
2. The crystallography data are avaliable at https://www.ccdc.cam.ac.uk/ CCDC NO.s: compound 6a: 2249289, compound 10a: 2254233.
3. NMR, HMRS, cordinates of optimized structures and energentics data, IC50s and molecular docking model are avaliable in supplementary file.
4. PTP1B co-crystal structure was free-downloaded from https://www.rcsb.org/ (PDBID: 1T4J)

Research involving human participants, their data, or biological material

Policy information about studies with human participants or human data. See also policy information about sex, gender (identity/presentation), and sexual orientation and race, ethnicity and racism.

Reporting on sex and gender	N/A
Reporting on race, ethnicity, or other socially relevant groupings	N/A
Population characteristics	N/A
Recruitment	N/A
Ethics oversight	N/A

Note that full information on the approval of the study protocol must also be provided in the manuscript.

Field-specific reporting

Please select the one below that is the best fit for your research. If you are not sure, read the appropriate sections before making your selection.

☒ Life sciences ☐ Behavioural & social sciences ☐ Ecological, evolutionary & environmental sciences

For a reference copy of the document with all sections, see nature.com/documents/nr-reporting-summary-flat.pdf

Life sciences study design

All studies must disclose on these points even when the disclosure is negative.

Sample size	1. For the synthesis, 23 substrates were evaluated to demonstrate the feasibility of the standard reactions and 11 substrates were tested for the rearrangement/cyclization transformation.<br>2.For the PTP inhibitory activity evaluation, 3 samples were set for each compound according to conventional PTP assays.
Data exclusions	For bioassay data, the IC50 values are not fittable if the following conditions were met:<br>1. Less than 4 points were adopted for the non-linear curve fit<br>2. R2 is less than 0.9<br>3. The inibition rate is >40% for the lowest concentration
Replication	To ensure robust reproducibility: All bioassay data presented in this manuscript were repeated two times. The standard reactions were repeated 3 times.
Randomization	The experiments in this study are at molecular level and the samples and softwares used are more feasible to obtain a good quality-control and to generalize, comparing to animal study. But, we did consider different investigators in the study may influence the results. Therefore, we replicated the key experiments by 3 different investigators, and the results were consistent.
Blinding	The evaluation of bioactivity of 3-hydroxy oxindoles were conducted in blinding. The compounds structure and resources information was unknown to the investigator who conducted bioactivity test, to ensure the objective data collection and analysis.

Behavioural & social sciences study design

All studies must disclose on these points even when the disclosure is negative.

Study description	N/A
Research sample	N/A
Sampling strategy	N/A
Data collection	N/A
Timing	N/A
Data exclusions	N/A
Non-participation	N/A
Randomization	N/A

Ecological, evolutionary & environmental sciences study design

All studies must disclose on these points even when the disclosure is negative.

Study description	N/A
Research sample	N/A
Sampling strategy	N/A
Data collection	*Describe the data collection procedure, including who recorded the data and how.*
Timing and spatial scale	N/A
Data exclusions	N/A
Reproducibility	N/A
Randomization	N/A
Blinding	N/A

Did the study involve field work? ☐ Yes ☒ No

Reporting for specific materials, systems and methods

We require information from authors about some types of materials, experimental systems and methods used in many studies. Here, indicate whether each material, system or method listed is relevant to your study. If you are not sure if a list item applies to your research, read the appropriate section before selecting a response.

Materials & experimental systems

n/a	Involved in the study
☒	☐ Antibodies
☒	☐ Eukaryotic cell lines
☒	☐ Palaeontology and archaeology
☒	☐ Animals and other organisms
☒	☐ Clinical data
☒	☐ Dual use research of concern
☒	☐ Plants

Methods

n/a	Involved in the study
☒	☐ ChIP-seq
☒	☐ Flow cytometry
☒	☐ MRI-based neuroimaging

Plants

Seed stocks	N/A
Novel plant genotypes	N/A
Authentication	N/A
